# Prevalence and severity of neurologic symptoms in Long-COVID and the role of pre-existing conditions, hospitalization, and mental health

**DOI:** 10.3389/fneur.2025.1562084

**Published:** 2025-06-25

**Authors:** Hanalise V. Huff, Henry Roberts, Elizabeth Bartrum, Gina Norato, Nicholas Grayson, Katherine Fleig, Miciah J. Wilkerson, Barbara J. Stussman, Avindra Nath, Brian Walitt

**Affiliations:** National Institutes of Health, National Institute of Neurological Diseases and Stroke, Bethesda, MD, United States

**Keywords:** COVID-19, neurologic symptoms, Long-COVID, nervous system, post-acute sequelae of SARS-CoV-2

## Abstract

**Background:**

Long-COVID refers to ongoing, relapsing, or new symptoms present 30 or more days after Severe Acute Respiratory Syndrome Coronavirus 2 (SARS-CoV-2) infection. This study examined the prevalence and severity of neurologic symptoms at greater than 1 month following acute SARS-CoV-2 infection and the influence of pre-existing neurologic and psychiatric conditions, current depression and anxiety status, and hospitalization on the presence and severity of these symptoms.

**Methods:**

This prospective cohort study recruited primarily self-referred Long-COVID participants with confirmed SARS-CoV-2 infection. Online questionnaires inquiring about pre-existing conditions, neurologic symptoms and their severity pre, during and post COVID-19, and current anxiety and depression screening were completed by 213 participants at a median time of 8 months after infection. Descriptive analyses and prevalence modeling were performed.

**Results:**

The most frequent neurologic symptoms post COVID-19 were fatigue, concentration/memory difficulties, unrefreshed sleep, and dysarthria/word finding difficulties (73.2–86.4%). Neurologic symptoms were highly prevalent with significantly greater odds post COVID-19 compared to pre for all symptoms and higher prevalence at time periods farther from infection, including those implicit in fibromyalgia and myalgic encephalomyelitis/chronic fatigue syndrome. Several severe neurologic symptoms were significantly more prevalent post COVID-19. Moderate to severe anxiety (34%) and depression (27%) were observed post COVID-19. Preexisting neurologic or psychiatric conditions did not demonstrate any significant difference in neurologic symptom prevalence post COVID-19. Those who met criteria for moderate or severe anxiety post COVID-19 had a significant difference in prevalence of fatigue, sensitivity to touch and unrefreshed sleep. Similarly, fatigue, concentration/memory difficulty and unrefreshed sleep were more prevalent in moderate to severe depression. There were no significant differences in neurologic symptom prevalence in a hospitalized group when compared to non- hospitalized.

**Conclusion:**

Long-COVID has a high burden of long lasting and severe neurological sequelae. These sequelae are independent of pre-existing self-reported neurologic and psychiatric conditions, as well as previous hospitalization. Current moderate to severe anxiety and depression status can impact fatigue, cognition, and sleep post COVID-19. Focus on the biological impact of SARS-CoV-2 on the nervous system will be essential in ameliorating the tremendous symptom burden left in the wake of the COVID-19 pandemic.

**Clinical trial registration:**

http://clinicaltrials.gov, identifier: NCT04573062.

## Introduction

Since the early days of the COVID-19 pandemic, it has been evident that a proportion of survivors of Severe Acute Respiratory Syndrome Coronavirus 2 (SARS-CoV-2) infection develop a persistent constellation of symptoms. Long-COVID or Post-acute sequelae of SARS-CoV-2 infection (PASC) has been defined by the National Academies of Science, Engineering and Medicine (NASEM) as an infection-associated chronic condition occurring after SARS-CoV-2 infection, presenting for a duration of 3 months or more as a continuous, relapsing, and remitting, or progressive disease state affecting one of more organ system (1).” The timing of those symptoms in relation to onset of COVID-19 is not specified (2). A WHO-led Delphi process Long-COVID definition included that the condition occurs a minimum of 3 months from COVID-19 and must last a duration of at least 2 months and cannot be explained by an alternate diagnosis (3). The National Institute for Health and Care Excellence (NICE), defines Long COVID as including “ongoing symptomatic COVID-19″ (symptoms from 4 to 12 weeks) and “post-COVID-19 syndrome” (symptoms that continue for more than 12 weeks following COVID-19) (4). The CDC found that 6.4% of US non-institutionalized adults reported having ever experiencing Long-COVID based on 2022 data (5). With so many impacted by SARS-CoV-2 globally, it is important to understand the types of Long-COVID symptoms, their course, and predisposing factors in order to predict who may be most affected and provide the best medical care.

Some cases of Long-COVID are from sequelae of injury to cardiac muscle, lung tissue, or kidneys and tend to follow expected recovery courses (6). Acute SARS-CoV-2 infection also can occasionally result in neurologic injury from stroke, encephalitis, encephalopathy and neuropathy (7). These tissue injuries are more common with severe COVID-19 acuity, yet Long-COVID symptoms are pervasive in survivors regardless of COVID-19 severity (8).

Long-COVID is described as a cluster of symptoms that include fatigue, dyspnea, cognitive difficulties, myalgias, arthralgias, headache, dizziness, and sleep difficulties (9). The term “brain fog” became a common description of perceived issues with concentration, information processing, memory and executive function (10). These neurocognitive and neuropsychiatric symptoms parallel those arising as the sequelae of other viral, bacterial and parasitic infections, and are now recognized as “post acute infection syndromes (11)” or “infection associated chronic illnesses (12).”

The psychological impact of SARS-CoV-2 is an important consideration in the characterization of Long- COVID. After the 2003 SARS-CoV-1 outbreak, survivors had a higher prevalence of psychological symptoms when compared to controls at one year (13). Almost 43% of SARS-CoV-1 survivors experienced psychiatric illness and there was no significant difference in illness severity, comorbidities or complications in survivors with psychiatric symptoms compared to those without. A similar number of survivors (40%) reported chronic fatigue that was not associated with SARS-CoV-1 severity yet was associated with having a current psychiatric disorder (14).

Common Long-COVID symptoms are also the hallmarks of other medical illnesses, including fibromyalgia, myalgic encephalomyelitis/chronic fatigue syndrome (ME/CFS), and chronic inflammatory response syndrome. The predisposing factors, etiology and pathophysiology of these disorders remain poorly understood and there are no established consensus treatments (15). Understanding Long-COVID will likely impact the science and healthcare management of these other illnesses.

Here we aim to characterize the prevalence and severity of neurologic symptoms in patients self-referred to the National Institute of Health (NIH) for Long-COVID. We compared participants’ symptoms before and after their SARS-CoV-2 infections and used the 2020 US population symptom data to guide generalizability. The impact of pre-existing neurologic and psychiatric conditions, current psychiatric status, and hospitalization on neurologic symptom outcomes was also examined.

## Methods

### Participants

This prospective cohort study (NCT04573062) recruited participants into a natural history study on persistent symptoms following COVID-19. Recruitment was conducted primarily via self-referral through the NIH patient recruitment office, NIH Clinical Trial web page,^1^ and clinician referrals. Between July 2020 and July 2023, 872 inquiries were received.

Participants included met these criteria: age 18 years or older, confirmed positive test for SARS-CoV-2 infection, greater than 4 weeks post-acute infection, had not fully recovered following COVID-19, completed all required questionnaires, and provided informed consent. Of the 872 inquiries, 213 participants met inclusion criteria (Supplementary Figure 1). All data collected were related to a participant’s first SARS-CoV-2 infection; none had reported being reinfected during the time period queried.

### Data collection

Participants were emailed a login link to an online database system (CiSTAR) to access 22 internet-based questionnaires (Supplementary Figure 2) which they had 1 month to complete in order to reduce recall bias. When possible, the questionnaires used validated questions drawn from the National Institute of Neurological Disorders and Stroke Common Data Elements^2^ and validated questionnaires which are publicly available (16). This was to ensure consistent participant comprehension of questions and improve reliability of data collected. Each participant’s recollections of the presence and severity of 54 symptoms pre, during infection, and post COVID-19 were collected. Twenty-nine neurological symptoms were the focus of this analysis.

Participants completed questions related to current depression and anxiety using Patient-Reported Outcomes Measurement Information System (PROMIS) item banks. Whether a participant met fibromyalgia criteria was determined using the 2016 fibromyalgia epidemiologic criteria (17). Post- exertional malaise severity was determined using a modified version of the DePaul Symptom Questionnaire (18). Participants entered questionnaire answers directly into CiSTAR. Collected data were exported and managed using Microsoft Excel.

### Demographic and clinical variables

Estimates regarding SARS-CoV-2 variants were based on CDC date ranges (19). Acute SARS-CoV-2 severity and hospitalization were categorized using the WHO Severity Scale (20). Details for how each participant was categorized for the 29 neurologic symptoms in three different time periods (pre, during and post-COVID), their prevalence and severity, pre-existing conditions, and COVID-19 test status are presented in Supplementary Table 1. If a participant chose “unsure” for a symptom or condition, they were considered not to have that symptom or condition. Across all symptoms, the average prevalence of “unsure” was 0.9% pre, 4.3% during, and 2.4% post. Depression and anxiety prevalence and severity were determined using appropriate PROMIS categories. A participant was considered to have a pre-existing neurological or psychiatric condition if they answered “yes” to one or more of the neurologic or psychiatric condition questions, respectively.

### Statistical analysis

#### Demographics

Patient demographics and characteristics were descriptively analyzed. Categorical variables were described by counts and percentages; continuous and ordinal variables by medians and interquartile ranges (IQR). To understand generalizability to the US population, these distributions were compared to similar distributions of 2016–2020 National Health Interview Survey (NHIS) (21) respondents.

#### Prevalence of neurologic symptom analysis

To assess the association between the occurrences of neurologic symptoms pre vs. post COVID-19, unadjusted odds ratio estimates from generalized estimating equations (GEE) were calculated, with pre-COVID being the reference time period.

#### Prevalence accounting for pre-existing conditions

The point prevalences and confidence intervals of pre-existing neurologic conditions were estimated for each neurologic symptom, overall and at each of three time-periods (Pre, During, and Post COVID-19 infection). Pre vs. post COVID-19 infection analysis was performed to test if the prevalence of a neurologic symptom was dependent on the time-period and the presence of pre-existing neurologic and psychiatric conditions. GEE models with an unstructured correlation structure accounting for within- subject correlations were used to model the log-prevalence of neurologic symptoms while adjusting for the effect of (1) the pre vs. post time-period, (2) presence of pre-existing neurologic condition, (3) presence of pre-existing psychiatric condition, (4) and all three of these variables together. Each model was comprised of a dependent variable (neurologic symptom) and 3 independent variables (time-period, pre-existing neurologic condition, and pre-existing psychiatric condition).

#### Symptom severity analysis

For each of the 29 neurologic symptoms, participants were categorized into severe and not severe symptom groups. An analysis of prevalence ratio estimates of symptom severity was conducted to test if symptom severity was more prevalent pre versus post-COVID-19. The effect of pre-existing psychiatric and neurologic conditions on reporting severe depression and anxiety post-COVID-19 was conducted using odds ratio analysis.

#### Time analysis

The impact of continuous time from SARS-CoV-2 infection on neurologic symptom prevalence post- COVID-19 was determined using Wilcoxon Rank-Sum tests, to account for any distributional irregularities of the data. For visualization, the prevalence of each neurologic symptom was described across three- month blocks of elapsed time from the onset of acute COVID-19 symptoms, using a heatmap. The symptoms were sorted by mean prevalence overall, from least to most prevalent. Symptoms that were significantly related to continuous time on the aforementioned tests are below the horizontal line, and non-significant are above the horizontal line. To describe the symptom burden trajectory, the number of individuals in the cohort that experienced symptoms of each severity level pre, during, and post COVID-19 were shown using bar graphs. Symptoms were clustered by category of neurologic symptom.

Statistics and visualizations were performed in R (version 4.2.0) and SAS 9.4 (22). *p*-values <0.05 were classified as significant and all results regardless of significance were considered in terms of clinical significance and in light of confidence interval width.

### Standard protocol approvals, registrations, and patient consents

All research procedures were approved by the NIH Central IRB (protocol 000089) and performed in accordance with *45 CFR 46 Protection of Human Subjects*. Informed consent was obtained on all study participants.

## Results

### Demographics

**Figure 1:**
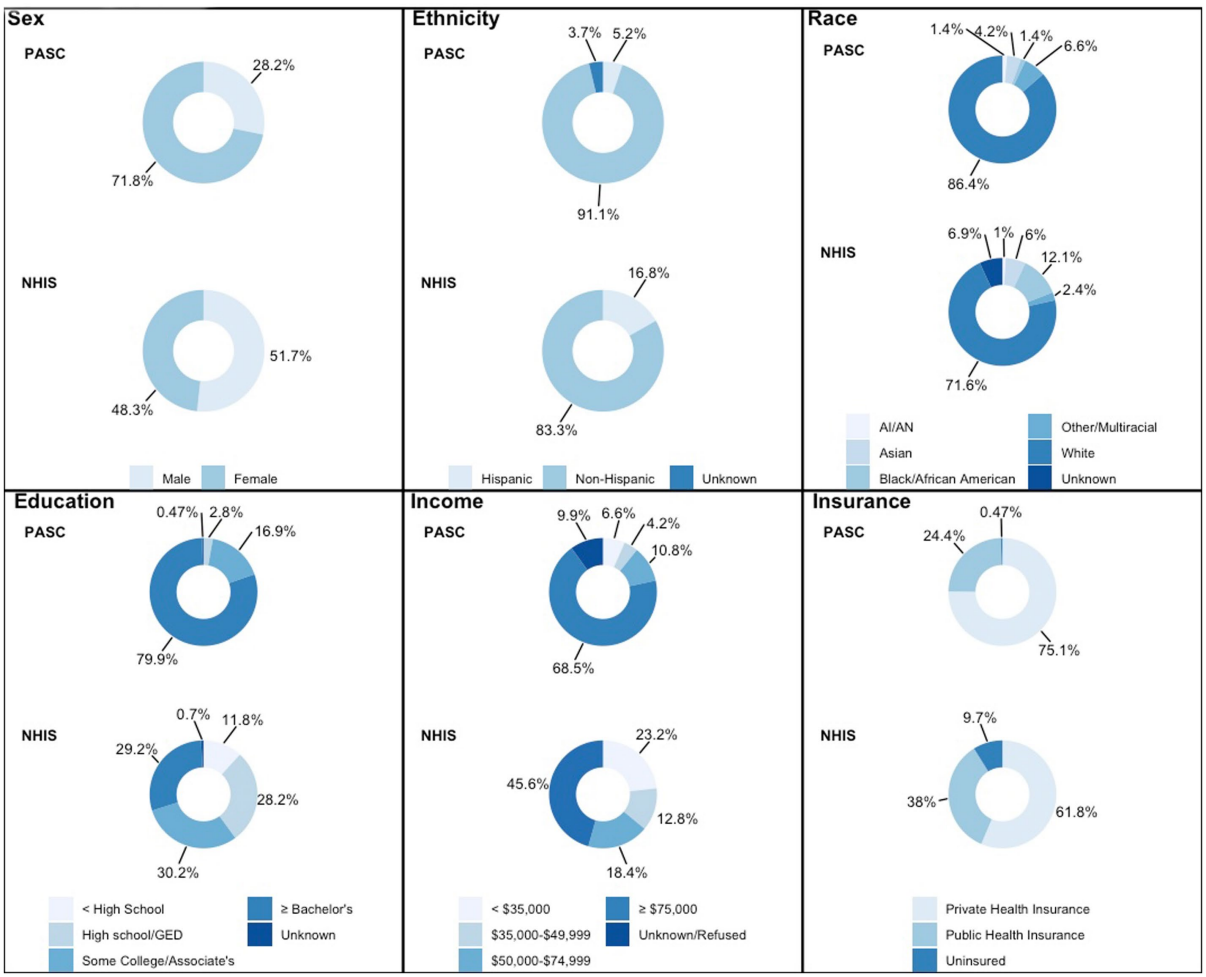
Demographic information. Each of six boxes represent a different demographic category (sex, ethnicity, race, education, income, insurance). Colors on the donut graph indicate the prevalence in a percentage of each sociodemographic variable for each category. The top donut for each category represents this cohort’s demographic breakdown while the bottom donut represents the NHIS data for the general US population.

The study cohort (*n* = 213) ranged in age from 20 to 76 (median 44 years; interquartile range [IQR] 35 to 56). The cohort was primarily comprised of females (71.8%) and persons who identified as non-Hispanic white (86.4%). A bachelor’s degree or higher was held by 170 (79.8%) participants, 146 (68.5%) indicated an annual household income of $75,000 or more and 160 (75.1%) possessed private health insurance (Figure 1). A comparison of the cohort’s demographic characteristics with 2020 National Health Interview Survey (NHIS) US population estimates is displayed in Supplementary Table 2.

### Infection information

The dates of infection ranged from February 14, 2020 to December 18, 2022. The median elapsed time from infection to questionnaire completion was 8 months (IQR 4.98 to 11.9). To estimate cohort vaccination status, we selected March 1^st^, 2021 to be the index date for COVID-19 vaccination availability. Based on this estimation, at least 164 (77%) were infected prior to vaccination. Per CDC estimation of variant dates, 70% were infected with the initial SARS-CoV-2 strains (L/alpha), 11.3% with beta/gamma, 4.2% with Delta and 14.5% with omicron. 33 participants (16%) were hospitalized related to COVID-19 and none were intubated (Supplementary Table 2).

### Pre-existing conditions

Sixty-eight participants (31.9%) had at least one neurologic pre-existing condition. Conditions included: prior migraine or headache diagnosis (59), history of stroke or Transient Ischemic Attack (2), seizure or epilepsy (7), multiple sclerosis (1), prior Guillain Barre Syndrome (1), prior concussion (2), peripheral neuropathy (1), and Postural Orthostatic Tachycardia Syndrome or Exercise Induced Hypotension (2). History of migraines or headaches comprised 27.7% of our cohort compared with 15.5% in the 2020 NHIS population.

One hundred (46.9%) participants had at least one psychiatric pre-existing condition. Compared to the 2020 NHIS population, 69 participants (32.4%) had pre-existing depression vs. 16.5% and 82 (38.5%) reported pre-existing anxiety vs. 15% (21).

### Prevalence of neurologic symptoms

Symptom prevalence pre, during and post SARS-CoV-2 are presented in Table 1. Trouble sleeping was the most common symptom pre COVID-19 (31%), followed by headache (26.8%), arthralgia (22.1%), fatigue (20.7%) and myalgia (19.7%).

**Table 1:** Neurologic symptom pre, during and post-COVID-19 prevalence and odds ratios of symptom onset post-COVID.

Symptom	Pre N (%)	During N (%)	Post N (%)	Pre vs. post unadjusted OR (95% CI)	*p*-value	NHIS symptom time frame	NHIS prevalence (%)
Fever	3 (1.4)	108 (50.7)	6 (2.8)	2 (0.5–8.3)	0.33	–	–
Chills	8 (3.8)	162 (76.1)	44 (20.7)	6.7 (3.3–13.7)	<0.001	–	–
“Flu-like symptoms”	10 (4.7)	167 (78.4)	36 (16.9)	4.1 (2.0–8.7)	<0.001	–	–
Cough	24 (11.3)	158 (74.2)	57 (26.8)	2.9 (1.8–4.5)	<0.001	–	–
Loss of appetite	6 (2.8)	134 (62.9)	60 (28.2)	13.5 (5.8–31.4)	<0.001	–	–
General weakness	8 (3.8)	168 (78.9)	127 (59.6)	37.8 (18.3–78.4)	<0.001	–	–
Fatigue	44 (20.7)	203 (95.3)	184 (86.4)	24.4 (15.3–38.9)	<0.001	Severe fatigue in past 12 months^1^	13.8
Hair loss	12 (5.6)	67 (31.5)	73 (34.3)	8.7 (4.9–15.6)	<0.001	–	–
Nausea/vomiting	10 (4.7)	99 (46.5)	64 (30.0)	8.7 (4.8–15.9)	<0.001	Stomach problem with vomiting or diarrhea in past 2 weeks^2^	4.9
Dizziness/lightheadedness	20 (9.4)	126 (59.2)	130 (61.0)	15.1 (9.1–25.1)	<0.001	Feeling lightheaded, without a sense of motion in past 12 months^1^	13.1
Skin rash	13 (6.1)	51(23.9)	50 (23.5)	4.7 (2.6–8.5)	<0.001	–	–
Trouble sleeping	66 (31.0)	126 (59.2)	140 (65.7)	4.3 (3.1–5.9)	<0.001	Trouble falling asleep most days or every day in past 30 days^3^ Trouble staying asleep most days or every day in past 30 days^3^	52.0 50.0
Unrefreshed sleep	60 (28.2)	144 (67.6)	161 (75.6)	12.2 (6.5–22.8)	<0.001	–	–
Difficulty staying awake	11 (5.2)	114(53.5)	85 (39.9)	7.9 (5.4–11.5)	<0.001	–	–
Concentration/ memory difficulties	20 (9.4)	162 (76.1)	183 (85.9)	58.9 (33.1–104.7)	<0.001	At least some difficulty remembering or concentrating currently^3^	17.5
Dysarthria/word finding difficulty	9(4.2)	128 (60.1)	156 (73.2)	62 (29.5–130.3)	<0.001	–	–
Focal weakness	7 (3.3)	42 (19.7)	110 (51.6)	31.4 (14.2–69.6)	<0.001	–	–
Balance/coordination difficulty	11 (5.2)	86 (40.4)	94 (44.1)	14.5 (7.8–26.9)	<0.001	Dizziness or balance problem in past 12 months^1^	17.1
Numbness/tingling	18 (8.5)	53 (24.9)	125 (58.7)	15.4 (9.1–26.0)	<0.001	–	–
Touch sensitivity	6 (2.8)	22(10.3)	33 (15.5)	6.3 (2.9–13.8)	<0.001	–	–
Noise sensitivity	25 (11.7)	73 (34.3)	92 (43.2)	5.7 (3.7–8.9)	<0.001	–	–
Light sensitivity	24 (11.3)	69 (32.4)	80 (37.6)	4.7 (3.0–7.4)	<0.001	–	–
See specks or flashes of light	22 (10.3)	59 (27.7)	71 (33.3)	5.7 (3.7–8.9)	<0.001	–	–
Blurred vision	11 (5.2)	80(37.6)	95 (44.6)	14.8 (8.0–27.2)	<0.001	–	–
Myalgia	42 (19.7)	151(70.9)	121 (56.8)	5.4 (3.7–7.8)	<0.001	–	–
Arthralgia	47 (22.1)	120(56.3)	101 (47.4)	3.2 (2.3–4.4)	<0.001	Chronic joint symptoms in past 3 months^2^	34.3
Headaches	57 (26.8)	176 (82.6)	126 (59.2)	4 (2.7–5.9)	<0.001	Migraine/severe headache in past 3 months^2^	15.5
Delirium	2 (0.9)	65 (30.5)	47 (22.1)	29.9 (7.0–126.8)	<0.001	–	–
Hallucinations	1 (0.5)	19(8.9)	14 (6.6)	14.9 (1.9–115.6)	0.01	–	–

NHIS pulled from years: ^1^2016, ^2^2018, ^3^2020.

Over 50% reported fever, and over 75% reported “flu-like symptoms,” cough, and chills during COVID-19. Less than 5% reported these symptoms pre and less than 27% post. During COVID-19, fatigue was the most prevalent symptom (95.3%). Other common symptoms during COVID-19 included: headache (82.6%), concentration difficulty/memory difficulties (76.1%), myalgia (70.9%), arthralgia (56.3%), dysarthria/word finding difficulty (60.1%), trouble sleeping (59.2%), difficulty staying awake (53.5%), and dizziness/lightheadedness (59.2%).

The most prevalent symptom post-COVID-19 was also fatigue (86.4%). However, concentration/memory difficulties (85.9%), dysarthria/word finding difficulty (73.2%), and trouble sleeping (65.7%) continued to increase in prevalence post-COVID. Other symptoms with high prevalence post-COVID-19 included dizziness/lightheadedness (61%), headache (59.2%), general weakness (59.6%), numbness/tingling (58.7%), myalgia (56.8), and focal weakness (51.6%).

#### Relative comparisons of neurologic symptoms pre vs. post COVID-19

The odds for all symptoms except fever increased significantly after SARS-CoV-2 infection as shown in Table 1. The symptoms with highest likelihood to be attributable to COVID-19 were dysarthria/word finding difficulty [OR 62, 95% CI 29.5–130.3] and concentration/memory issues [OR 58.9, 95% CI 33.1–104.7]. General weakness [OR 37.8, 95% CI 18.3–78.4], focal weakness [OR 31.4, 95% CI 14.2–69.6], delirium [OR 29.9, 95%CI 7–126.8] and fatigue [OR 24.4, 95% CI 15.3–38.9] were also found to be highly associated with COVID-19.

### Neurologic symptoms and pre-existing conditions

#### Neurologic symptoms in participants with pre-existing neurologic conditions

There were no substantial differences in prevalence of neurologic symptoms post COVID-19 between participants with pre-existing neurologic condition compared with those without (Table 2). Complaint of “chills” (Prevalence ratio (PR) 1.8, 95% CI 1.1–3.0) was the largest difference; all other symptoms ranged between PR 0.8 to 1.3. Many of the statistically significant differences in PR pre-COVID-19 between the groups disappeared post-COVID-19.

**Table 2:** Prevalence ratios of neurologic symptoms in those with and without pre-existing neurologic conditions.

Symptom	Neurologic preexisting condition	Prevalence estimates		Prevalence ratios		
		Pre-COVID	Post-COVID	Comparison	PR (95% CL)	*p*-value
Fever	Neurologic	4.4%	0.0%	PreNeuro vs. PreNonNeuro	N/A—Model did not Converge	
	Non-Neurologic	0.0%	4.1%	PostNeuro vs. PostNonNeuro		
Chills	Neurologic	7.4%	29.4%	PreNeuro vs. PreNonNeuro	3.6 (0.9–14.4)	0.08
	Non-Neurologic	2.1%	16.6%	PostNeuro vs. PostNonNeuro	1.8 (1.1–3.0)	0.03
Flu-like symptoms	Neurologic	10.3%	14.7%	PreNeuro vs. PreNonNeuro	5.0 (1.3–18.7)	0.02
	Non-Neurologic	2.1%	17.9%	PostNeuro vs. PostNonNeuro	0.8 (0.4–1.6)	0.56
Cough	Neurologic	16.2%	30.9%	PreNeuro vs. PreNonNeuro	1.8 (0.9–3.8)	0.12
	Non-Neurologic	9.0%	24.8%	PostNeuro vs. PostNonNeuro	1.2 (0.8–2.0)	0.35
Loss of appetite	Neurologic	7.4%	33.8%	PreNeuro vs. PreNonNeuro	10.7 (1.3–89.5)	0.03
	Non-Neurologic	0.7%	25.5%	PostNeuro vs. PostNonNeuro	1.3 (0.9–2.0)	0.2
General weakness	Neurologic	8.8%	55.9%	PreNeuro vs. PreNonNeuro	6.4 (1.3–30.9)	0.02
	Non-Neurologic	1.4%	61.4%	PostNeuro vs. PostNonNeuro	0.9 (0.7–1.2)	0.54
Fatigue	Neurologic	32.4%	88.2%	PreNeuro vs. PreNonNeuro	2.1 (1.3–3.6)	0.004
	Non-Neurologic	15.2%	86.2%	PostNeuro vs. PostNonNeuro	1.0 (0.9–1.1)	0.67
Hair loss	Neurologic	10.3%	32.4%	PreNeuro vs. PreNonNeuro	3.0 (1.0–9.1)	0.05
	Non-Neurologic	3.5%	35.2%	PostNeuro vs. PostNonNeuro	0.9 (0.6–1.4)	0.69
Nausea/vomiting	Neurologic	7.4%	35.3%	PreNeuro vs. PreNonNeuro	2.1 (0.6–7.1)	0.22
	Non-Neurologic	3.5%	27.6%	PostNeuro vs. PostNonNeuro	1.3 (0.8–1.9)	0.25
Dizziness/lightheadedness	Neurologic	16.2%	58.8%	PreNeuro vs. PreNonNeuro	2.6 (1.1–6.0)	0.02
	Non-Neurologic	6.2%	62.1%	PostNeuro vs. PostNonNeuro	0.9 (0.7–1.2)	0.66
Skin rash	Neurologic	4.4%	27.9%	PreNeuro vs. PreNonNeuro	0.6 (0.2–2.2)	0.49
	Non-Neurologic	6.9%	21.4%	PostNeuro vs. PostNonNeuro	1.3 (0.8–2.1)	0.29
Trouble sleeping	Neurologic	41.2%	70.6%	PreNeuro vs. PreNonNeuro	1.6 (1.1–2.3)	0.02
	Non-Neurologic	26.2%	63.5%	PostNeuro vs. PostNonNeuro	1.1 (0.9–1.4)	0.29
Difficulty staying awake	Neurologic	5.9%	42.7%	PreNeuro vs. PreNonNeuro	1.2 (0.4–4.0)	0.75
	Non-Neurologic	4.8%	38.6%	PostNeuro vs. PostNonNeuro	1.1 (0.8–1.6)	0.57
Unrefreshed sleep	Neurologic	35.3%	82.4%	PreNeuro vs. PreNonNeuro	1.4 (0.9–2.2)	0.11
	Non-Neurologic	24.8%	72.4%	PostNeuro vs. PostNonNeuro	1.1 (1.0–1.3)	0.09
Concentration/memory difficulties	Neurologic	14.7%	86.8%	PreNeuro vs. PreNonNeuro	2.1 (0.9–4.9)	0.07
	Non-Neurologic	6.9%	85.5%	PostNeuro vs. PostNonNeuro	1.0 (0.9–1.1)	0.8
Dysarthria/word finding difficulty	Neurologic	8.8%	69.1%	PreNeuro vs. PreNonNeuro	4.3 (1.1–16.5)	0.04
	Non-Neurologic	2.1%	75.2%	PostNeuro vs. PostNonNeuro	0.9 (0.8–1.1)	0.37
Focal weakness	Neurologic	4.4%	48.5%	PreNeuro vs. PreNonNeuro	1.6 (0.4–6.9)	0.53
	Non-Neurologic	2.8%	53.1%	PostNeuro vs. PostNonNeuro	0.9 (0.7–1.2)	0.54
Balance/coordination difficulty	Neurologic	11.8%	47.1%	PreNeuro vs. PreNonNeuro	5.7 (1.6–20.8)	0.009
	Non-Neurologic	2.1%	42.8%	PostNeuro vs. PostNonNeuro	1.1 (0.8–1.5)	0.55
Numbness/tingling	Neurologic	17.7%	57.4%	PreNeuro vs. PreNonNeuro	4.3 (1.7–10.9)	0.002
	Non-Neurologic	4.1%	59.3%	PostNeuro vs. PostNonNeuro	1.0 (0.8–1.2)	0.79
Touch sensitivity	Neurologic	4.4%	17.7%	PreNeuro vs. PreNonNeuro	2.1 (0.4–10.3)	0.35
	Non-Neurologic	2.1%	14.5%	PostNeuro vs. PostNonNeuro	1.2 (0.6–2.3)	0.55
Noise sensitivity	Neurologic	25.0%	44.1%	PreNeuro vs. PreNonNeuro	4.5 (2.1–10.0)	<0.001
	Non-Neurologic	5.5%	42.8%	PostNeuro vs. PostNonNeuro	1.0 (0.7–1.4)	0.85
Light sensitivity	Neurologic	17.7%	39.7%	PreNeuro vs. PreNonNeuro	2.1 (1.0–4.5)	0.05
	Non-Neurologic	8.3%	36.6%	PostNeuro vs. PostNonNeuro	1.1 (0.8–1.6)	0.65
See specks or flashes of light	Neurologic	13.2%	33.8%	PreNeuro vs. PreNonNeuro	1.5 (0.7–3.3)	0.34
	Non-Neurologic	9.0%	33.1%	PostNeuro vs. PostNonNeuro	1.0 (0.7–1.5)	0.92
Blurred vision	Neurologic	8.8%	41.2%	PreNeuro vs. PreNonNeuro	2.6 (0.8–8.1)	0.11
	Non-Neurologic	3.5%	46.2%	PostNeuro vs. PostNonNeuro	0.9 (0.6–1.2)	0.5
Myalgia	Neurologic	35.3%	58.8%	PreNeuro vs. PreNonNeuro	2.8 (1.7–4.9)	<0.001
	Non-Neurologic	12.4%	55.9%	PostNeuro vs. PostNonNeuro	1.1 (0.8–1.3)	0.68
Arthralgia	Neurologic	25.0%	47.1%	PreNeuro vs. PreNonNeuro	1.2 (0.7–2.0)	0.48
	Non-Neurologic	20.7%	47.6%	PostNeuro vs. PostNonNeuro	1.0 (0.7–1.3)	0.94
Headache	Neurologic	83.8%	63.2%	PreNeuro vs. PreNonNeuro	N/A - Model did not Converge	
	Non-Neurologic	0.0%	57.2%	PostNeuro vs. PostNonNeuro		
Delirium	Neurologic	2.9%	25.0%	PreNeuro vs. PreNonNeuro	N/A - Model did not Converge	
	Non-Neurologic	0.0%	20.7%	PostNeuro vs. PostNonNeuro		
Hallucinations	Neurologic	1.5%	2.9%	PreNeuro vs. PreNonNeuro	N/A - Model did not Converge	
	Non-Neurologic	0.0%	8.3%	PostNeuro vs. PostNonNeuro		

#### Neurologic symptoms in participants with pre-existing psychiatric conditions

Pre-existing psychiatric conditions did not appear to have a substantial impact on post-COVID symptoms, as shown in Table 3. Several symptoms were found to have a significant difference between those with and without pre-existing psychiatric conditions but only in the period pre-COVID, including fatigue, trouble sleeping, unrefreshed sleep, concentration/memory difficulties, numbness/tingling, noise sensitivity, and light sensitivity. Nausea/vomiting and myalgia were found to have a significant difference between those with and without pre-existing conditions in both pre- and post-COVID. Arthralgia was the only symptom found to be significant between groups in the period post-COVID.

**Table 3:** Prevalence ratios of neurologic symptoms in those with and without pre-existing psychiatric conditions.

Symptom	Psychiatric status	Prevalence estimates		Prevalence ratios		
		Pre-COVID	Post-COVID	Comparison	PR (95% CL)	*p*-value
Fever	Psychiatric	2.0%	3.0%	PrePsych vs. PreNonPsych	2.3 (0.2–24.5)	0.5
	Non-Psychiatric	0.9%	2.7%	PostPsych vs. PostNonPsych	1.1 (0.2–5.5)	0.88
Chills	Psychiatric	5.0%	25.0%	PrePsych vs. PreNonPsych	1.9 (0.5–7.7)	0.38
	Non-Psychiatric	2.7%	16.8%	PostPsych vs. PostNonPsych	1.5 (0.9–2.5)	0.14
Flu-like symptoms	Psychiatric	6.0%	19.0%	PrePsych vs. PreNonPsych	1.7 (0.5–5.8)	0.4
	Non-Psychiatric	3.5%	15.0%	PostPsych vs. PostNonPsych	1.3 (0.7–2.3)	0.44
Cough	Psychiatric	8.0%	31.0%	PrePsych vs. PreNonPsych	0.6 (0.3–1.3)	0.16
	Non-Psychiatric	14.2%	23.0%	PostPsych vs. PostNonPsych	1.3 (0.9–2.1)	0.19
Loss of appetite	Psychiatric	4.0%	33.0%	PrePsych vs. PreNonPsych	2.3 (0.4–12.1)	0.34
	Non-Psychiatric	1.8%	23.9%	PostPsych vs. PostNonPsych	1.4 (0.9–2.1)	0.14
General weakness	Psychiatric	4.0%	62.0%	PrePsych vs. PreNonPsych	1.1 (0.3–4.4)	0.86
	Non-Psychiatric	3.5%	57.5%	PostPsych vs. PostNonPsych	1.1 (0.9–1.3)	0.51
Fatigue	Psychiatric	29.0%	89.0%	PrePsych vs. PreNonPsych	2.2 (1.2–3.8)	0.006
	Non-Psychiatric	13.3%	85.0%	PostPsych vs. PostNonPsych	1.0 (0.9–1.2)	0.38
Hair loss	Psychiatric	4.0%	38.0%	PrePsych vs. PreNonPsych	0.6 (0.2–1.8)	0.34
	Non-Psychiatric	7.1%	31.0%	PostPsych vs. PostNonPsych	1.3 (0.8–1.8)	0.28
Nausea/vomiting	Psychiatric	8.0%	37.0%	PrePsych vs. PreNonPsych	4.5 (1.0–20.8)	0.05
	Non-Psychiatric	1.8%	23.9%	PostPsych vs. PostNonPsych	1.5 (1.0–2.3)	0.04
Dizziness/lightheadedness	Psychiatric	11.0%	64.0%	PrePsych vs. PreNonPsych	1.4 (0.6–3.2)	0.45
	Non-Psychiatric	8.0%	58.4%	PostPsych vs. PostNonPsych	1.1 (0.9–1.4)	0.4
Skin rash	Psychiatric	9.0%	29.0%	PrePsych vs. PreNonPsych	2.5 (0.8–8.0)	0.11
	Non-Psychiatric	3.5%	18.6%	PostPsych vs. PostNonPsych	1.6 (1.0–2.6)	0.08
Trouble sleeping	Psychiatric	38.0%	66.0%	PrePsych vs. PreNonPsych	1.5 (1.0–2.3)	0.04
	Non-Psychiatric	24.8%	65.5%	PostPsych vs. PostNonPsych	1.0 (0.8–1.2)	0.94
Difficulty staying awake	Psychiatric	6.0%	44.0%	PrePsych vs. PreNonPsych	1.4 (0.4–4.3)	0.61
	Non-Psychiatric	4.4%	36.3%	PostPsych vs. PostNonPsych	1.2 (0.9–1.7)	0.25
Unrefreshed sleep	Psychiatric	36.0%	78.0%	PrePsych vs. PreNonPsych	1.7 (1.1–2.6)	0.02
	Non-Psychiatric	21.2%	73.5%	PostPsych vs. PostNonPsych	1.1 (0.9–1.2)	0.44
Concentration/memory difficulties	Psychiatric	14.0%	88.0%	PrePsych vs. PreNonPsych	2.6 (1.1–6.6)	0.04
	Non-Psychiatric	5.3%	84.1%	PostPsych vs. PostNonPsych	1.0 (0.9–1.2)	0.41
Dysarthria/word finding difficulty	Psychiatric	4.0%	79.0%	PrePsych vs. PreNonPsych	0.9 (0.2–3.3)	0.88
	Non-Psychiatric	4.4%	68.1%	PostPsych vs. PostNonPsych	1.2 (1.0–1.4)	0.07
Focal weakness	Psychiatric	3.0%	55.0%	PrePsych vs. PreNonPsych	0.9 (0.2–3.7)	0.83
	Non-Psychiatric	3.5%	48.7%	PostPsych vs. PostNonPsych	1.1 (0.9–1.5)	0.36
Balance/coordination difficulty	Psychiatric	5.0%	43.0%	PrePsych vs. PreNonPsych	0.9 (0.3–3.0)	0.92
	Non-Psychiatric	5.3%	45.1%	PostPsych vs. PostNonPsych	1.0 (0.7–1.3)	0.75
Numbness/tingling	Psychiatric	13.0%	59.0%	PrePsych vs. PreNonPsych	2.9 (1.1–8.0)	0.03
	Non-Psychiatric	4.4%	58.4%	PostPsych vs. PostNonPsych	1.0 (0.8–1.3)	0.93
Touch sensitivity	Psychiatric	4.0%	19.0%	PrePsych vs. PreNonPsych	2.3 (0.4–12.1)	0.34
	Non-Psychiatric	1.8%	12.4%	PostPsych vs. PostNonPsych	1.5 (0.8–2.9)	0.19
Noise sensitivity	Psychiatric	17.0%	50.0%	PrePsych vs. PreNonPsych	2.4 (1.1–5.3)	0.03
	Non-Psychiatric	7.1%	37.2%	PostPsych vs. PostNonPsych	1.3 (1.0–1.8)	0.06
Light sensitivity	Psychiatric	16.0%	41.0%	PrePsych vs. PreNonPsych	2.3 (1.0–5.1)	0.05
	Non-Psychiatric	7.1%	34.5%	PostPsych vs. PostNonPsych	1.2 (0.8–1.7)	0.33
See specks or flashes of light	Psychiatric	12.0%	39.0%	PrePsych vs. PreNonPsych	1.4 (0.6–3.0)	0.45
	Non-Psychiatric	8.9%	28.3%	PostPsych vs. PostNonPsych	1.4 (0.9–2.0)	0.1
Blurred vision	Psychiatric	5.0%	48.0%	PrePsych vs. PreNonPsych	0.9 (0.3–3.0)	0.92
	Non-Psychiatric	5.3%	41.6%	PostPsych vs. PostNonPsych	1.2 (0.9–1.6)	0.35
Myalgia	Psychiatric	26.0%	69.0%	PrePsych vs. PreNonPsych	1.8 (1.1–3.2)	0.03
	Non-Psychiatric	14.2%	46.0%	PostPsych vs. PostNonPsych	1.5 (1.2–1.9)	<0.001
Arthralgia	Psychiatric	24.0%	54.0%	PrePsych vs. PreNonPsych	1.2 (0.7–2.0)	0.52
	Non-Psychiatric	20.4%	41.6%	PostPsych vs. PostNonPsych	1.3 (1.0–1.7)	0.07
Headache	Psychiatric	32.0%	58.0%	PrePsych vs. PreNonPsych	1.4 (0.9–2.3)	0.11
	Non-Psychiatric	22.1%	60.2%	PostPsych vs. PostNonPsych	1.0 (0.8–1.2)	0.75
Delirium	Psychiatric	1.0%	24.0%	PrePsych vs. PreNonPsych	1.1 (0.7–17.8)	0.93
	Non-Psychiatric	0.9%	20.4%	PostPsych vs. PostNonPsych	1.2 (0.7–2.0)	0.52
Hallucinations	Psychiatric	0.0%	8.0%	PrePsych vs. PreNonPsych	0.0 (*)	*
	Non-Psychiatric	0.9%	5.3%	PostPsych vs. PostNonPsych	1.5 (0.5–4.2)	0.43

*Mantel–Haenszel 95%CL not available.

#### Prevalence of neurologic symptoms adjusted for pre-existing conditions

The relation of neurologic symptoms with pre-existing neurologic and psychiatric conditions is presented in Table 4. Prevalence ratios were similar when performed unadjusted and adjusted for time, pre-existing neurologic, and pre-existing psychiatric conditions. Having a pre-existing condition did not alter the odds of any neurologic symptom except headache. Pre-existing neurologic participants reported having more headaches pre-COVID (83.6%) than post-COVID (63.24%). This is in contradistinction to participants without pre-existing neurologic conditions, where 0% reported headaches pre-COVID and 57.2% reported headaches post-COVID. In summary, pre-existing neurologic or psychiatric conditions did not have a negative impact on prevalence of any of the 29 symptoms.

**Table 4:** Adjusted prevalence ratios of neurologic symptoms and risk of severe neurologic symptoms post-COVID.

Symptoms	Prevalence estimates		Adjusted prevalence ratios*			Severity %			Risk of severe symptoms post-COVID		
	Time period	%	ARR	95% CL	*p*-value	Severe	Not Severe	No Sx	ARR	95% CL	*p*-value
Fever	Pre-COVID	1.4	2.0	0.5–8.0	0.3	0.0%	1.4%	98.6%	N/A	N/A	N/A
	Post-COVID	2.8				0.5%	2.3%	97.2%			
Chills	Pre-COVID	3.8	5.5	2.8–10.8	<0.001	0.5%	3.3%	96.2%	N/A	N/A	N/A
	Post-COVID	20.7				0.0%	20.7%	79.3%			
Flu-like symptoms	Pre-COVID	4.7	3.6	1.8–7.1	<0.001	0.5%	4.2%	95.3%	7.0	1.1–43.0	0.04
	Post-COVID	16.9				3.3%	13.6%	83.1%			
Cough	Pre-COVID	11.3	2.4	1.6–3.5	<0.001	0.5%	10.8%	88.7%	3.0	0.3–28.9	0.3
	Post-COVID	26.8				1.4%	23.9%	74.7%			
Loss of appetite	Pre-COVID	2.8	10.0	9.9 (4.4–22.1)	<0.001	0.5%	2.3%	97.2%	11.9	1.8–78.1	0.01
	Post-COVID	28.2				5.6%	22.5%	71.8%			
General weakness	Pre-COVID	3.8	15.9	8.1–31.3	<0.001	0.9%	2.8%	96.2%	15.5	3.9–62.0	<0.001
	Post-COVID	59.6				14.6%	45.1%	40.4%			
Fatigue	Pre-COVID	20.7	4.2	3.2–5.4	<0.001	0.5%	20.2%	79.3%	81.2	11.3–582.2	<0.001
	Post-COVID	86.9				38.0%	48.8%	13.1%			
Hair loss	Pre-COVID	5.6	6.1	3.6–10.4	<0.001	0.0%	5.6%	94.4%	N/A	N/A	N/A
	Post-COVID	34.3				7.9%	26.8%	65.3%			
Nausea/ vomiting	Pre-COVID	4.7	6.4	3.6–11.3	<0.001	0.0%	4.7%	95.3%	N/A	N/A	N/A
	Post-COVID	30.0				4.7%	25.4%	70.0%			
Dizziness/ lightheadedness	Pre-COVID	9.4	6.5	4.3–9.9	<0.001	0.0%	9.4%	90.6%	N/A	N/A	N/A
	Post-COVID	61.0				7.5%	53.5%	39.0%			
Skin rash	Pre-COVID	6.1	3.8	2.2–6.6	<0.001	0.0%	5.6%	94.4%	N/A	N/A	N/A
	Post-COVID	23.5				3.3%	20.2%	76.5%			
Trouble sleeping	Pre-COVID	31.0	2.1	1.8–2.5	<0.001	0.9%	30.0%	69.0%	20.0	5.2–77.2	<0.001
	Post-COVID	65.7				18.8%	46.9%	34.3%			
Difficulty staying awake	Pre-COVID	5.2	7.7	4.4–13.7	<0.001	0.0%	5.2%	94.8%	N/A	N/A	N/A
	Post-COVID	39.9				8.5%	31.5%	60.1%			
Unrefreshed sleep	Pre-COVID	28.2	2.7	2.2–3.3	<0.001	2.3%	25.8%	71.8%	10.8	4.5–25.7	<0.001
	Post-COVID	75.6				25.4%	50.2%	24.4%			
Concentration/ Memory difficulties	Pre-COVID	9.4	9.1	6.0–13.9	<0.001	0.9%	8.5%	90.6%	35.0	8.8–139.9	<0.001
	Post-COVID	85.9				32.9%	53.1%	14.1%			
Dysarthria/word finding difficulty	Pre-COVID	4.2	17.4	9.1–33.2	<0.001	0.0%	4.2%	95.8%	N/A	N/A	N/A
	Post-COVID	73.2				16.9%	56.3%	26.8%			
Focal weakness	Pre-COVID	3.3	15.7	7.5–32.9	<0.001	0.0%	3.3%	96.7%	N/A	N/A	N/A
	Post-COVID	51.6				11.3%	40.4%	48.4%			
Balance/coordination difficulty	Pre-COVID	5.2	8.5	4.8–15.0	<0.001	0.0%	5.2%	94.8%	N/A	N/A	N/A
	Post-COVID	44.1				6.6%	37.6%	55.9%			
Numbness/tingling	Pre-COVID	8.5	6.9	4.4–10.8	<0.001	0.0%	8.5%	91.5%	N/A	N/A	N/A
	Post-COVID	58.7				11.3%	47.4%	41.3%			
Touch sensitivity	Pre-COVID	2.8	5.5	2.6–11.6	<0.001	0.0%	2.8%	97.2%	N/A	N/A	N/A
	Post-COVID	15.5				2.3%	13.1%	84.5%			
Noise sensitivity	Pre-COVID	11.7	3.6	2.5–5.2	<0.001	0.9%	10.8%	88.3%	11.5	2.9–45.9	<0.001
	Post-COVID	43.2				10.8%	32.9%	56.3%			
Light sensitivity	Pre-COVID	11.3	3.3	2.3–4.8	<0.001	0.0%	11.3%	88.7%	N/A	N/A	N/A
	Post-COVID	37.6				7.5%	30.1%	62.4%			
See specks or flashes of light	Pre-COVID	10.3	3.2	2.2–4.7	<0.001	0.0%	10.3%	89.7%	N/A	N/A	N/A
	Post-COVID	33.3				3.3%	30.0%	66.7%			
Blurred vision	Pre-COVID	5.2	8.7	4.9–15.2	<0.001	0.0%	5.2%	94.8%	N/A	N/A	N/A
	Post-COVID	44.6				7.0%	37.1%	55.9%			
Myalgia	Pre-COVID	19.7	2.8	2.2–3.7	<0.001	0.0%	19.7%	80.3%	N/A	N/A	N/A
	Post-COVID	56.8				15.5%	41.3%	43.2%			
Arthralgia	Pre-COVID	22.1	2.2	1.7–2.7	<0.001	0.0%	22.1%	77.9%	N/A	N/A	N/A
	Post-COVID	47.4				12.7%	34.7%	52.6%			
Headache	Pre-COVID	26.8	1.4	1.2–1.8	0.001	24.9%	73.2%	1.9%	10.8	4.1–28.7	<0.001
	Post-COVID	59.2				19.7%	39.4%	40.8%			
Delirium	Pre-COVID	0.9	23.4	5.7–96.6	<0.001	0.0%	0.9%	99.1%	N/A	N/A	N/A
	Post-COVID	22.1				6.1%	16.0%	77.9%			
Hallucinations	Pre-COVID	0.5	14.1	1.9–106.4	0.01	0.0%	0.5%	99.5%	N/A	N/A	N/A
	Post-COVID	6.6				0.5%	5.6%	93.9%			

*Presented here are prevalence ratios adjusted for neurologic comorbidity status and psychiatric comorbidity status. The unadjusted values were the same if not marginally different from the adjusted and therefore not shown.

### Risk of having severe neurologic symptoms post-COVID

Several symptoms were more frequently severe post-COVID when compared to pre-COVID (Table 4). These include fatigue (PR 81.2 [95% CI 11.3–582.2]), concentration/memory issues (PR 35 [95% CI 8.8–139.9]), unrefreshed sleep (PR 10.8 [95% CI 4.5–25.7]) and trouble sleeping (PR 20 [95% CI 5.2–77.2]).

**Figure 2:**
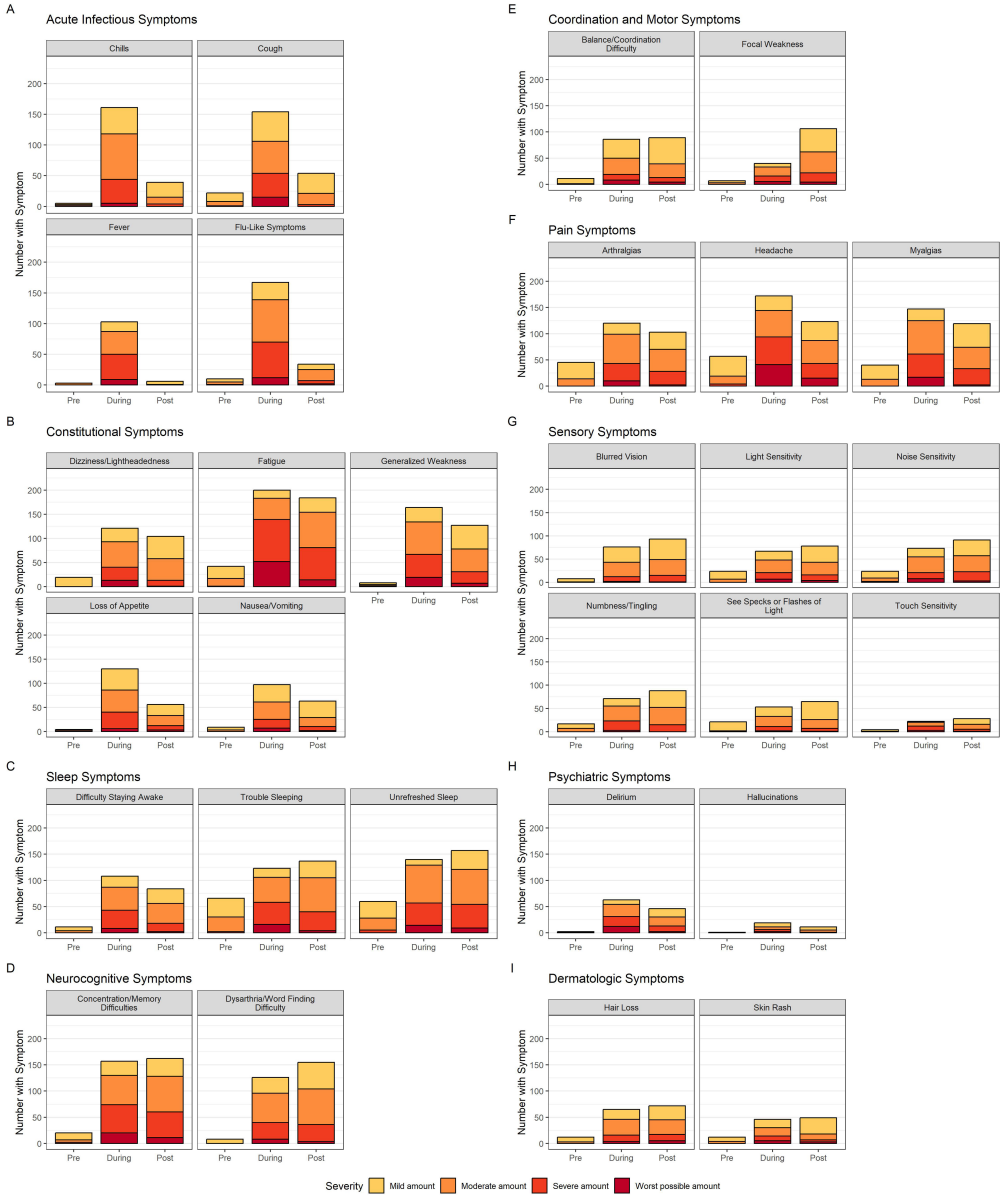
Neurologic symptom prevalence and severity bar graphs. The 29 neurologic symptoms are divided into 9 categories (acute infectious, constitutional, sleep, neurocognitive, coordination and motor, pain, sensory, psychiatric and dermatologic). Each symptom’s bar graph has three bars representing time of symptom in regards to acute COVID-19 in chronological order (pre, during, post). The colors within each bar represent the breakdown of participant-rated severity (yellow-mild, orange-moderate, light red-severe, dark red-worst possible). Color legend is at the bottom of the figure.

The distribution of symptom severity during each time period is displayed in Figure 2. As expected, acute infectious symptoms uniformly show low prevalence and mild severity pre-COVID, a dramatic increase in both prevalence and severity during, and finally a precipitous drop off post-COVID. In contrast, constitutional and pain symptoms have a low pre-COVID prevalence and severity, a dramatic increase in prevalence and severity during infection, and a modest decrease in prevalence and severity post-COVID. Sleep, neurocognitive, coordination and motor, sensory, and dermatologic symptoms also have low pre-COVID prevalence, a marked rise in prevalence and severity during, but continue to increase in prevalence post-COVID. With these persisting symptoms, severity during infection does not change substantially post-COVID.

### Effect of elapsed time from infection on post-COVID symptom prevalence

**Figure 3:**
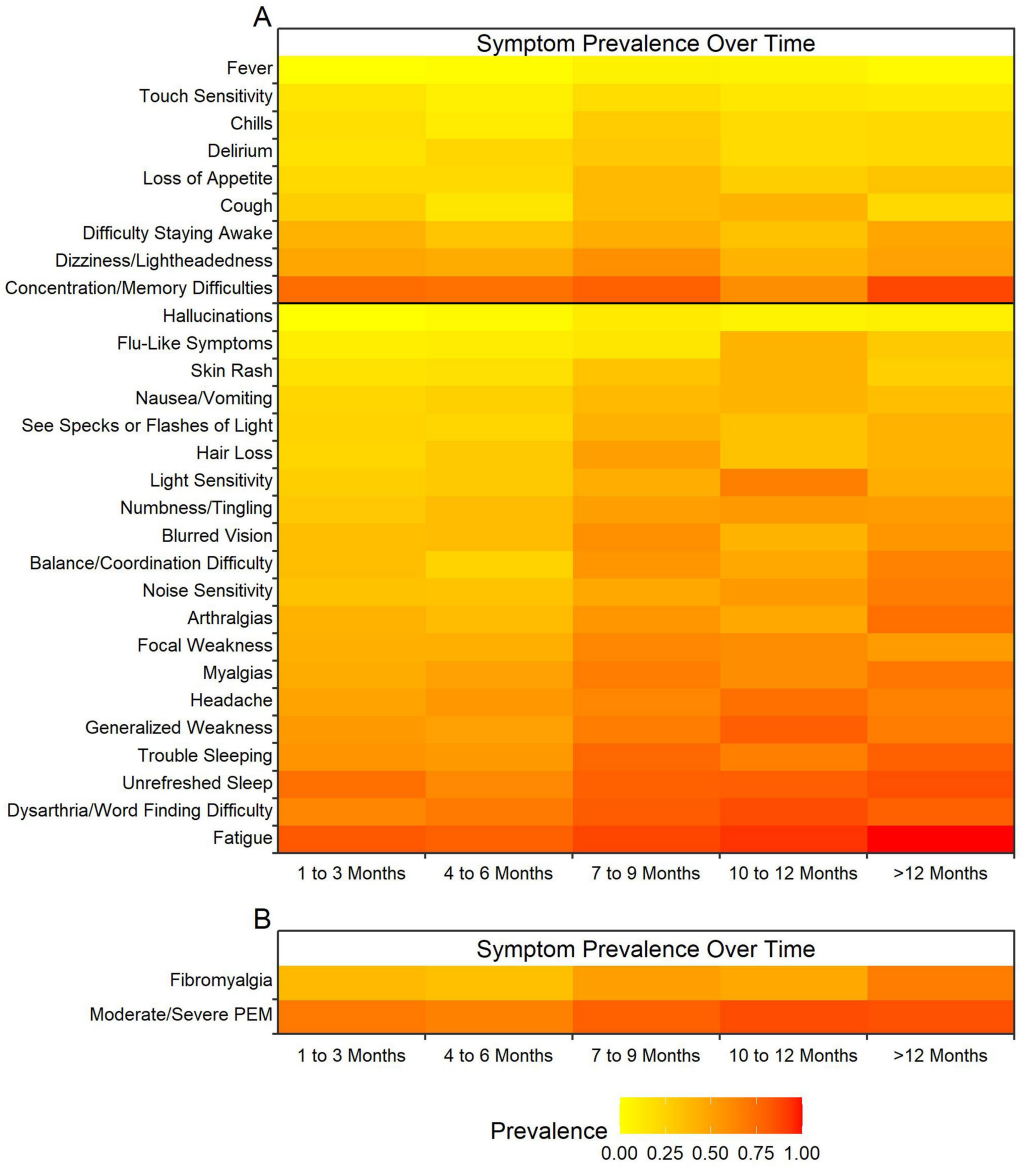
Heatmap of effect of elapsed time from infection on post-COVID symptom prevalence. Colors represent prevalence with yellow representing least prevalent up to dark red representing most prevalent at almost 100% of the cohort (see legend at bottom of figure). The X-axis represents time in three-month intervals when the post-COVID-19 symptoms were reported between 1 month and 12 months. **(A)** Elapsed time between SARS-CoV-2 infection and time when the 29 neurologic symptoms (Y-axis) were reported post COVID-19. Black bar indicates significance with 20 symptoms below the bar being statistically significant and the 9 symptoms above being insignificant. **(B)** Elapsed time between SARS-CoV-2 infection and time for symptoms meeting criteria for fibromyalgia using fibromyalgia outcome score and for symptoms meeting criteria for moderate to severe post-exertional malaise (PEM) using PEM outcome score. Both variables showed elapsed time between infection and measurement to have a significant impact on the outcome prevalences.

The elapsed time between SARS-CoV-2 infection and the participant’s enrollment has a significant impact on the reported prevalence of 20 of the 29 symptoms queried (Figure 3A). Fatigue, word finding difficulties, unrefreshed sleep, general weakness, and pain issues were the most impacted by elapsed time, with greater prevalence at time periods farther from infection. Not all symptoms were impacted by elapsed time, including concentration/memory difficulties, dizziness, and difficulty staying awake.

Meeting the 2016 fibromyalgia criteria (20) and having moderate or severe post-exertional malaise were also impacted by elapsed time, with more participants meeting severity criteria over time (Figure 3B). It is notable that severe post-exertional malaise was infrequent across all timepoints.

### Anxiety and depression

#### Prevalence of anxiety and depression

Using PROMIS scales, nearly half of the cohort met criteria for anxiety and/or depression after COVID-19. However, for many participants these symptoms were mild. Moderate to severe anxiety was observed in 34% and moderate to severe depression was observed in 27% of the cohort (Supplementary Table 3).

#### Anxiety and depression status in participants with pre-existing neurologic and psychiatric conditions

Pre-existing neurologic conditions did not increase the odds of meeting criteria post-COVID for anxiety alone, depression alone, or both anxiety and depression together (Supplementary Table 3). Having a pre- existing psychiatric condition increased the odds of meeting criteria for having moderate to severe anxiety [OR 2.6 (1.1–6.1), *p* = 0.02], depression [OR 2.3 (0.7–7.0), *p* = 0.15], or both [OR 3.5 (1.7–7.6), *p* = 0.001] post-COVID (Supplementary Table 3).

#### Neurologic symptoms and anxiety and depression status

The impact of post-COVID moderate to severe depression and anxiety on neurologic symptoms is displayed in Table 5. Participants with moderate or severe depression at the time of the survey were more likely to report several neurologic symptoms including: fatigue (OR 5.6; 1.3–24.4), general weakness (OR 2.1; 1.1–1.4), focal weakness (OR 1.9; 1.0–3.6), loss of appetite (OR 2.1; 1.1–4.1), concentration/ memory difficulties (OR 3.2; 0.9–11), dizziness/lightheadedness (OR 2.3; 1.1–4.5), noise sensitivity (OR 2.0; 1.1–3.8), touch sensitivity (OR 2.5; 1.2–5.5), see specks/flashes of light (OR 2.6; 1.4–4.9), myalgia (OR 2.7; 1.4–5.5), and unrefreshed sleep (OR 2.8; 1.2–6.6). Those with moderate or severe anxiety at time of survey were more likely to report fatigue (OR 3.5;1.2–10.4), loss of appetite (OR 2.7; 1.4–5.1), general weakness (OR 2.3; 1.2–4.2), focal weakness (OR 1.9; 1.1–3.5), nausea/vomiting (2.0; 1.1–3.8), balance/coordination difficulty (OR 2.1; 1.1–4.0), numbness/tingling (OR 2.5; 1.3–4.6), touch sensitivity (OR 3.1; 1.4–6.7), blurred vision (OR 1.9; 1.1–3.4), myalgia (OR 2.1; 1.2–4.0), delirium (OR 2.1, 1.1–4.1), and unrefreshed sleep (OR 3.0;1.3–6.5).

**Table 5:** Odds ratios of neurologic symptoms for those with and without depression and anxiety.

Symptoms n (%)	Prevalence in people with depression Score ≥ 60* (*N* = 54)	Prevalence in people without depression Score < 60 (*N* = 147)	Odds ratio (95% CI)	*p*-value	Prevalence in people with anxiety score ≥ 60 (*N* = 68)	Prevalence in people without anxiety score < 60 (*N* = 135)	Odds ratio (95% CI)	*p*-value
Fever	0 (0)	5 (3)	–	–	2 (3)	4 (3)	1.0 (0.2–5.6)	0.99
Chills	14 (26)	28 (19)	1.5 (0.7–3.1)	0.29	19 (28)	24 (18)	1.8 (0.9–3.6)	0.1
“Flu-like symptoms”	10 (19)	26 (18)	1.1 (0.5–2.4)	0.89	17 (25)	19 (14)	2.0 (1.0–4.2)	0.06
Cough	14 (26)	40 (27)	0.9 (0.5–1.9)	0.86	25 (37)	30 (22)	2.0 (1.1–3.9)	0.03
Loss of appetite	21 (39)	34 (23)	2.1 (1.1–4.1)	0.03	28 (41)	28 (21)	2.7 (1.4–5.1)	0.003
General weakness	39 (72)	82 (56)	2.1 (1.1–4.1)	0.04	49 (72)	72 (53)	2.3 (1.2–4.2)	0.01
Fatigue	52 (96)	121 (82)	5.6 (1.3–24.4)	0.02	64 (94)	111 (82)	3.5 (1.2–10.4)	0.03
Hair loss	19 (35)	49 (33)	1.1 (0.6–2.1)	0.81	26 (38)	44 (33)	1.3 (0.7–2.3)	0.43
Nausea/vomiting	21 (39)	38 (26)	1.8 (0.9–3.5)	0.07	27 (40)	33 (24)	2.0 (1.1–3.8)	0.03
Dizziness/lightheadedness	40 (74)	82 (56)	2.3 (1.1–4.5)	0.02	45 (66)	77 (57)	1.5 (0.8–2.7)	0.21
Skin rash	13 (24)	34 (23)	1.1 (0.5–2.2)	0.89	21 (31)	26 (19)	1.9 (1.0–3.7)	0.07
Trouble sleeping	40 (74)	92 (63)	1.7 (0.9–3.4)	0.13	50 (74)	84 (62)	1.7 (0.9–3.2)	0.11
Unrefreshed sleep	47 (87)	104 (71)	2.8 (1.2–6.6)	0.02	59 (87)	93 (69)	3.0 (1.3–6.5)	0.007
Difficulty staying awake	25 (46)	57 (39)	1.4 (0.7–2.6)	0.34	32 (47)	50 (37)	1.5 (0.8–2.7)	0.17
Concentration/ memory difficulties	51 (94)	124 (84)	3.2 (0.9–11.0)	0.07	62 (91)	115 (85)	1.8 (0.7–4.7)	0.23
Dysarthria/word finding difficulty	45 (83)	103 (70)	2.1 (1.0–4.7)	0.06	54 (79)	96 (71)	1.6 (0.8–3.1)	0.21
Focal weakness	34 (63)	69 (47)	1.9 (1.0–3.6)	0.05	42 (62)	62 (46)	1.9 (1.1–3.5)	0.03
Balance/coordination difficulty	31 (57)	57 (39)	2.1 (1.1–4.0)	0.02	36 (53)	53 (39)	1.7 (1.0–3.1)	0.06
Numbness/tingling	37 (69)	80 (54)	1.8 (0.9–3.5)	0.07	49 (72)	69 (51)	2.5 (1.3–4.6)	0.005
Touch sensitivity	14 (26)	18 (12)	2.5 (1.2–5.5)	0.02	18 (26)	14 (10)	3.1 (1.4–6.7)	0.004
Noise sensitivity	30 (56)	56 (38)	2.0 (1.1–3.8)	0.03	35 (51)	52 (39)	1.7 (0.9–3.1)	0.08
Light sensitivity	21 (39)	53 (36)	1.1 (0.6–2.2)	0.33	28 (41)	47 (35)	1.3 (0.7–2.4)	0.38
See specks or flashes of light	27 (50)	41 (28)	2.6 (1.4–4.9)	0.004	29 (43)	39 (29)	1.8 (1.0–3.4)	0.05
Blurred vision	26 (48)	64 (44)	1.2 (0.6–2.3)	0.56	38 (56)	54 (40)	1.9 (1.1–3.4)	0.03
Myalgia	40 (74)	75 (51)	2.7 (1.4–5.5)	0.004	47 (69)	69 (51)	2.1 (1.2–4.0)	0.02
Arthralgia	30 (56)	64 (44)	1.6 (0.9–3.0)	0.13	38 (56)	57 (42)	1.7 (1.0–3.1)	0.07
Headaches	35 (65)	81 (55)	1.5 (0.8–2.9)	0.22	42 (62)	75 (56)	1.3 (0.7–2.3)	0.4
Delirium	15 (28)	30 (20)	1.5 (0.7–3.1)	0.27	21 (31)	24 (18)	2.1 (1.1–4.1)	0.04
Hallucinations	3 (6)	10 (7)	0.8 (0.2–3.1)	0.75	6 (9)	7 (5)	1.8 (0.6–5.5)	0.32

### Impact of hospitalization on post-COVID symptoms

Hospitalization did not have much impact on post-COVID symptoms (Supplementary Table 4). Thirty-three participants were hospitalized due to acute SARS-CoV-2 infection, with none being intubated. There were no statistically significant differences in symptoms between hospitalized and unhospitalized participants except for noise sensitivity (OR 0.3; 0.1–0.7).

## Discussion

With over 700 million reported cases and over 7 million deaths attributable to COVID-19 (21, 23), the impact of SARS-CoV-2 infections have been immense. Many survivors report a constellation of neurologic symptoms that persist after infectious convalescence. This report provides estimates of the burden of neurologic symptoms in Long-COVID. As expected, the new onset of symptoms, including fatigue, dyscognition, pain, and poor sleep, were highly prevalent and quite severe in this Long-COVID cohort. Pre-existing neurological disease was not uncommon and was most frequently related to headaches.

However, having pre-existing neurological disease had negligible impact on the development of new neurological symptoms after SARS-CoV-2 infection. Pre-existing depression and anxiety were reported by about half of the cohort, in keeping with prior studies demonstrating that psychiatric distress is a risk factor for Long-COVID (24). However, pre-existing psychiatric distress appears to have little impact on the type or severity of Long-COVID neurological symptoms themselves. Having current depression and anxiety as part of Long-COVID was associated with increases in fatigue, dyscognition, and sleep problems, among other symptoms. While few participants (15%) were hospitalized for their SARS-CoV-2 infection, no notable differences in Long-COVID symptoms were observed when compared with those not hospitalized. The prevalence and severity of Long-COVID neurological symptoms appear to be independent of pre-existing neurologic and psychiatric conditions.

While the population studied was selected for reporting Long-COVID symptoms, the neurologic symptomatic experiences prior to getting ill resemble what is expected in the general population except for an over-representation of those reporting pre-existing depression and/or anxiety. Common symptoms experienced prior to COVID-19, such as fatigue, trouble sleeping, and arthralgia resemble what was noted in the 2020 NHIS survey of the US population. Our cohort was over-represented with pre-existing headaches as well as depression and anxiety, which suggests these problems may increase Long-COVID risk. Demographically, the cohort was skewed, with an over-representation of middle-aged white women with high levels of education and resources. However, the 3:1 female to male ratio reflects the well- documented predisposition for women to develop syndromic disorders, as observed in fibromyalgia (25), myalgic encephalomyelitis/ chronic fatigue syndrome (ME/CFS) (26), and in other Long-COVID cohorts (27).

The dramatic increase in neurologic symptoms found in this Long-COVID cohort echo what has been found in other studies. A large study of healthcare databases of the US Department of Veteran Affairs found that 71 per 1,000 persons had neurologic symptoms at 12 months following COVID-19 (28). A meta-analysis found that one in five patients with COVID-19 have cognitive impairment 12 weeks or more after the acute infection (29). What our data adds is observations of the temporal aspects of symptom onset. Some neurological symptoms, including fatigue, general weakness, balance difficulty, and pain are reported during the active SARS-CoV-2 infection and are also noted post-COVID with modest improvement others, such as sleep, word finding, focal weakness, and sensory complaints appear to become more prevalent after SARS-CoV-2 convalescence. For both patterns of symptom onset, the severity of symptoms does not change substantially over time. The complaints are clinically relevant, with moderate and severe complaints being both frequent and persistent. Across the cohort, the more time that had elapsed since the infection, the more prevalent neurological symptoms were observed to be. The symptoms most impacted by elapsed time included fatigue, word finding difficulty, sleep issues, pain, and post-exertional malaise, which are the core symptoms of fibromyalgia and ME/CFS. In general, longitudinal studies have noted that Long-COVID symptom prevalence and severity decreases over time. The RECOVER initiative observed that only 10% of their cohort continued to have Long-COVID symptoms at 6 months (30). A study of participants with pre- existing neurologic disease noted that one-third of the cohort had full Long-COVID symptom resolution at 6 months (31). Pathologic fatigue persisted in only 33% at 1 year follow up (32) and a meta-analysis found that neuropsychiatric symptoms substantially increased between mid and long term follow up at greater than 6 months (33). This discrepancy likely results in part from our interpolation of symptom time frame based on single time period data collected at 1 month or greater following COVID-19 and the possibility of selection bias for those with more symptoms and more severity presenting later in their course. Additionally, it is also difficult to attribute symptoms to COVID-19 especially at time points the farthest from COVID-19. We find that those participating farther from acute disease were more likely to meet clinical criteria for fibromyalgia (17) and ME/CFS. This suggests that Long-COVID cases may increasingly resemble fibromyalgia and ME/CFS when symptoms are present farther out and have not recovered spontaneously or have been misattributed.

The main goal of these analyses was to better understand the impact of pre-existing neurological and psychological disease on the development of Long-COVID neurological symptoms. The idea that prior issues with the nervous system would predispose to the development of new persistent symptoms is intuitive. However, these data do not support the idea of neurological vulnerability. Neither self-reported pre-existing neurological nor psychiatric conditions have a statistically significant impact on the development of neurological symptoms after SARS-CoV-2.

The prevalence of depression and anxiety in this Long-COVID cohort is in keeping with other studies (34). One study found that at six and 12 months, about 45% of COVID-19 survivors self-rated in the clinical range for depression, anxiety, PTSD and/or fatigue and that post-COVID fatigue was highly correlated with psychopathology ratings (32). Similarly, in a 1 year follow up study on survivors of the SARS outbreak of 2003, almost 43% experienced psychiatric symptoms including depression, PTSD and panic disorder following infection, however only 3% had a pre-existing psychiatric history. Both those with and without pre-existing psychiatric conditions experienced chronic fatigue problems suggesting that the psychiatric issues were not associated with the experience of fatigue (14).

In our cohort, having a pre-existing psychiatric condition did increase the odds of meeting the criteria for moderate or greater severity anxiety alone or with depression. Having moderate to severe anxiety had a significant impact on unrefreshed sleep, touch sensitivity, and loss of appetite, which are common symptoms of anxiety (35). Similarly, having moderate to severe depression after SARS-CoV-2 infection did have a significant impact on fatigue, unrefreshed sleep, and concentration/ memory difficulties. These symptoms are also typical of depression (35). These findings correspond to other reports demonstrating significant associations between self-reported symptoms and patient reported outcome measures of depression and anxiety post-COVID (36). One study found that concomitant depressive symptoms and sleep problems were strongly associated with fatigue scores post-COVID (37). Another study found that neuropsychiatric parameters associated strongly with overall burden of self-reported Long-COVID symptoms though similarly acknowledge the complex entanglement between psychiatric burden and overall Long-COVID symptom burden (34). The overlap of symptoms across medical and psychiatric disorders suggests that trying to use symptoms for assigning diagnoses in the absence of objective markers of disease is easily confounded.

Hospitalization had a negligible impact on the development of neurological symptoms. This comports with other studies that have observed that symptoms such as fatigue, sleep disturbance and disordered cognition are equally reported in hospitalized and non-hospitalized groups even though objective measures may differ (38).

This study had various limitations. Recruitment was performed without advertising or targeted outreach. Participants were often self-referred based on word of mouth and were willing to participate without any compensation for their time. Participants were likely motivated to participate to share their symptomatic experiences, the possibility of speaking with NIH experts about their problems, and the possibility of enrolling in other in-person NIH research studies, particularly treatment trials. This method of selection has the potential to over-recruit those with more severe cases thereby leading to an overrepresentation of symptom prevalence and severity than in the broader Long-COVID population. The recruitment bias and sampling bias issues limit the generalizability of the results.

At the time of study start (July 2020), there was no consensus definition of Long-COVID and little was known about symptom time course, therefore the inclusion criteria were left broad. Any participant with self-reported persistent symptoms at 1 month to approximately 1 year were included in the study and data on estimated time of neurologic symptom onset was limited if the symptom began after recovery from acute COVID-19. As a result, it is difficult to determine whether some of the participants fit within the NASEM definition of symptom onset occurring 3 months or more after acute COVID-19 or the WHO definition that symptoms must last at least 2 months in duration (3). As such, the broad inclusion criteria may have limited its specificity to Long-COVID as we know it now and accurate identification of symptoms directly attributable to COVID-19. Additionally, there is no scientific consensus on the maximum time interval that still allows for a Long-COVID diagnosis (39). Recall bias is also unavoidable given the survey-based nature and reliance on participant self-reporting and it may have a greater impact for those participants farther out from infection. Recall bias may particularly impact results regarding pre-COVID symptoms which may affect the accuracy of comparison and identification of pre- to post- changes in symptom prevalence and severity. The percent of participants that answered “unsure” for symptoms was low and did not vary greatly between the before and after symptom categories.

Further limiting this study, there was no unbiased control group leading to limited generalizability. Participants’ experience post-COVID was compared to their own self-reported recall of experience pre-COVID. As demonstrated in the comparison of our study population with the general population as represented through NCIS data, our population has an over-representation of individuals with pre-existing depression and anxiety. Given the overlap between many of the physical manifestations of depression and anxiety and those of Long-COVID, the interplay between this pre-existing anxiety and depression and the Long-COVID symptoms experienced in the absence of an unbiased control group is difficult to ascertain. One two-year retrospective cohort study found increased incidence of mood and anxiety disorders post-COVID was transient and had no overall excess of these diagnoses compared with other respiratory infections (40) thereby demonstrating the important information an unbiased control group adds.

We also are dependent on the accuracy of participant self-report of pre-existing diagnostic history. It is possible some participants who reported no pre-existing depression or anxiety had undiagnosed symptoms. No physical evaluation by a physician to rule out alternative diagnoses in this patient population was conducted in parallel with the questionnaires to rule out confounding diagnoses like fibromyalgia or Sjogren’s Syndrome. Lastly, while the sample size was adequate for determining the group differences reported, it was not large enough for making precise estimates, leading to wide confidence intervals for many symptoms.

In summary, this study demonstrates the symptomatic primacy of the nervous system, both during acute SARS-CoV-2 infection and afterwards. Given the global pervasiveness of COVID-19, understanding the predisposing factors, phenotype, and natural history of Long-COVID is of utmost importance. Pre-existing headache disorders, depression, and anxiety were over-represented in our cohort. Counterintuitively, both pre-existing neurologic and psychiatric conditions did not impact the prevalence of neurologic symptoms though having moderate or severe depression or anxiety post-COVID did. With increasing elapsed time from infection, the neurological symptoms resemble those of fibromyalgia and ME/CFS, suggesting that early intervention with disease-modifying therapies could have a lasting impact on neurologic Long COVID outcomes. Focus on the biological impact of SARS-CoV-2 on the nervous system will be essential in ameliorating the tremendous symptom burden left in the wake of the COVID-19 pandemic.

## Data Availability

The raw data supporting the conclusions of this article will be made available by the authors, without undue reservation.

## Footnotes

^1^ www.clinicaltrials.Gov

^2^ http://www.commondataelements.ninds.nih.gov/

## Supplementary material

Supplementary Figure 1 Prisma diagram for participant selection including reasoning for exclusions.

Supplementary Figure 2 Questionnaires used to provide data for this study.

## References

[1] National Academies of Sciences E, Medicine. A Long COVID definition: A chronic, systemic disease state with profound consequences. Washington, DC: The National Academies Press (2024). 39110819

[2] National Academies of Sciences E, Medicine, Health In: Goldowitz I Worku T Brown L Fineberg HV, editors. A Long COVID definition: A chronic, systemic disease state with profound consequences. Washington (DC): National Academies Press (US) (2024) 39110819

[3] Soriano JB Murthy S Marshall JC Relan P Diaz JV. A clinical case definition of post-COVID-19 condition by a Delphi consensus. Lancet Infect Dis. (2022) 22:e102–7. doi: 10.1016/S1473-3099(21)00703-9, PMID: 34951953 PMC8691845

[4] Excellence NIfHaC. COVID-19 rapid guidelines: Managing the long-term effects of COVID-19. (2020). Available online at: https://www.nice.org.uk/guidance/ng188 (Accessed April 15, 2025).

[5] Ford NDAA Dalton AF Singleton J Perrine CG Saydah S. Notes from the field: Long COVID prevalence among adults — United States, 2022. MMWR Morb Mortal Wkly Rep. (2024) 73:135–6. doi: 10.15585/mmwr.mm7306a4, PMID: 38359012 PMC10899083

[6] Walitt B Bartrum E. A clinical primer for the expected and potential post-COVID-19 syndromes. Pain Rep. (2021) 6:e887. doi: 10.1097/PR9.0000000000000887, PMID: 33615088 PMC7889402

[7] Koralnik IJ Tyler KL. COVID-19: a global threat to the nervous system. Ann Neurol. (2020) 88:1–11. doi: 10.1002/ana.25807, PMID: 32506549 PMC7300753

[8] Petersen MS Kristiansen MF Hanusson KD Danielsen ME á Steig B Gaini S. Long COVID in the Faroe Islands: a longitudinal study among nonhospitalized patients. Clin Infect Dis. (2021) 73:e4058–63. doi: 10.1093/cid/ciaa1792, PMID: 33252665 PMC7799340

[9] Nalbandian A Sehgal K Gupta A Madhavan MV McGroder C Stevens JS. Post-acute COVID-19 syndrome. Nat Med. (2021) 27:601–15. doi: 10.1038/s41591-021-01283-z, PMID: 33753937 PMC8893149

[10] Monje M Iwasaki A. The neurobiology of long COVID. Neuron. (2022) 110:3484–96. doi: 10.1016/j.neuron.2022.10.006, PMID: 36288726 PMC9537254

[11] Choutka J Jansari V Hornig M Iwasaki A. Unexplained post-acute infection syndromes. Nat Med. (2022) 28:911–23. doi: 10.1038/s41591-022-01810-6, PMID: 35585196

[12] National Academies of Sciences E, Medicine. Toward a common research agenda in infection-associated chronic illnesses: Proceedings of a workshop. Washington, DC: The National Academies Press (2024). 38648305

[13] Lee AM Wong JG McAlonan GM. Stress and psychological distress among SARS survivors 1 year after the outbreak. Can J Psychiatr. (2007) 52:233–40. doi: 10.1177/070674370705200405, PMID: 17500304

[14] Lam MH Wing YK Yu MW Leung CM Ma RC Kong AP. Mental morbidities and chronic fatigue in severe acute respiratory syndrome survivors: long-term follow-up. Arch Intern Med. (2009) 169:2142–7. doi: 10.1001/archinternmed.2009.384, PMID: 20008700

[15] Walitt B Singh K LaMunion SR Hallett M Jacobson S Chen K. Deep phenotyping of post-infectious myalgic encephalomyelitis/chronic fatigue syndrome. Nat Commun. (2024) 15:907. doi: 10.1038/s41467-024-45107-3, PMID: 38383456 PMC10881493

[16] Grinnon ST Miller K Marler JR Lu Y Stout A Odenkirchen J. National Institute of Neurological Disorders and Stroke common data element project - approach and methods. Clin Trials. (2012) 9:322–9. doi: 10.1177/1740774512438980, PMID: 22371630 PMC3513359

[17] Wolfe F Clauw DJ Fitzcharles MA Goldenberg DL Häuser W Katz RL. 2016 revisions to the 2010/2011 fibromyalgia diagnostic criteria. Semin Arthritis Rheum. (2016) 46:319–29. doi: 10.1016/j.semarthrit.2016.08.012, PMID: 27916278

[18] Cotler J Holtzman C Dudun C Jason LA. A Brief Questionnaire to Assess Post-Exertional Malaise. Diagnostics (Basel). (2018) 8:66. doi: 10.3390/diagnostics8030066, PMID: 30208578 PMC6165517

[19] CDC. Museum COVID-19 Timeline 2023. (2024). Available online at: https://www.cdc.gov/museum/timeline/covid19.html (accessed July 2024).

[20] Characterisation WHOWGotC, Management of C-i. A minimal common outcome measure set for COVID-19 clinical research. Lancet Infect Dis. (2020) 20:e192–7. doi: 10.1016/S1473-3099(20)30483-7 32539990 PMC7292605

[21] Statistics NCfH. National Health Interview Survey. 2021st ed. National Center for Health Statistics (NCHS) (2020).

[22] Inc SI. SAS. NC: Cary (2016).

[23] WHO. COVID-19 dashboard. (2024). Available online at: https://data.who.int/dashboards/covid19/cases (accessed on September 5, 2024 2024).

[24] Wang S Quan L Chavarro JE Slopen N Kubzansky LD Koenen KC. Associations of depression, anxiety, worry, perceived stress, and loneliness prior to infection with risk of post–COVID-19 conditions. JAMA Psychiatry. (2022) 79:1081–91. doi: 10.1001/jamapsychiatry.2022.2640, PMID: 36069885 PMC9453634

[25] Walitt B Nahin RL Katz RS Bergman MJ Wolfe F. The prevalence and characteristics of fibromyalgia in the 2012 National Health Interview Survey. PLoS One. (2015) 10:e0138024. doi: 10.1371/journal.pone.0138024, PMID: 26379048 PMC4575027

[26] Lim EJ Ahn YC Jang ES Lee SW Lee SH Son CG. Systematic review and meta-analysis of the prevalence of chronic fatigue syndrome/myalgic encephalomyelitis (CFS/ME). J Transl Med. (2020) 18:100. doi: 10.1186/s12967-020-02269-0, PMID: 32093722 PMC7038594

[27] Bai F Tomasoni D Falcinella C Barbanotti D Castoldi R Mulè G. Female gender is associated with long COVID syndrome: a prospective cohort study. Clin Microbiol Infect. (2022) 28:611.e9–e16. doi: 10.1016/j.cmi.2021.11.002, PMID: 34763058 PMC8575536

[28] Xu E Xie Y Al-Aly Z. Long-term neurologic outcomes of COVID-19. Nat Med. (2022) 28:2406–15. doi: 10.1038/s41591-022-02001-z, PMID: 36138154 PMC9671811

[29] Ceban F Ling S Lui LMW Lee Y Gill H Teopiz KM. Fatigue and cognitive impairment in post-COVID-19 syndrome: a systematic review and meta-analysis. Brain Behav Immun. (2022) 101:93–135. doi: 10.1016/j.bbi.2021.12.020, PMID: 34973396 PMC8715665

[30] Thaweethai T Jolley SE Karlson EW Levitan EB Levy B McComsey GA. Development of a definition of Postacute sequelae of SARS-CoV-2 infection. JAMA. (2023) 329:1934–46. doi: 10.1001/jama.2023.8823, PMID: 37278994 PMC10214179

[31] Shanley JE Valenciano AF Timmons G Miner AE Kakarla V Rempe T. Longitudinal evaluation of neurologic-post acute sequelae SARS-CoV-2 infection symptoms. Ann Clin Transl Neurol. (2022) 9:995–1010. doi: 10.1002/acn3.51578, PMID: 35702954 PMC9268882

[32] Mazza MG Palladini M De Lorenzo R. One-year mental health outcomes in a cohort of COVID-19 survivors. J Psychiatr Res. (2021) 145:118–24. doi: 10.1016/j.jpsychires.2021.11.031, PMID: 34894521 PMC8607816

[33] Premraj L Kannapadi NV Briggs J Seal SM Battaglini D Fanning J. Mid and long-term neurological and neuropsychiatric manifestations of post-COVID-19 syndrome: a meta-analysis. J Neurol Sci. (2022) 434:120162. doi: 10.1016/j.jns.2022.120162, PMID: 35121209 PMC8798975

[34] Masserini F Pomati S Cucumo V Nicotra A Maestri G Cerioli M. Assessment of cognitive and psychiatric disturbances in people with post-COVID-19 condition: a cross-sectional observational study. CNS Spectr. (2024) 29:640–51. doi: 10.1017/S1092852924002153, PMID: 39582177

[35] Association AP. Diagnostic and statistical manual of mental disorders. 5th Edn ed. Washington, DC: (2013).

[36] Chen AK Wang X McCluskey LP. Neuropsychiatric sequelae of long COVID-19: pilot results from the COVID-19 neurological and molecular prospective cohort study in Georgia, USA. Brain Behav Immun Health. (2022) 24:100491. doi: 10.1016/j.bbih.2022.100491, PMID: 35873350 PMC9290328

[37] Hartung TJ Neumann C Bahmer T Chaplinskaya-Sobol I Endres M Geritz J. Fatigue and cognitive impairment after COVID-19: a prospective multicentre study. EClinicalMedicine. (2022) 53:101651. doi: 10.1016/j.eclinm.2022.101651, PMID: 36133318 PMC9482331

[38] Perez Giraldo GS Ali ST Kang AK Patel TR Budhiraja S Gaelen JI. Neurologic manifestations of Long COVID differ based on acute COVID-19 severity. Ann Neurol. (2023) 94:146–59. doi: 10.1002/ana.26649, PMID: 36966460 PMC10724021

[39] Stefanou MI Palaiodimou L Bakola E Smyrnis N Papadopoulou M Paraskevas GP. Neurological manifestations of long-COVID syndrome: a narrative review. Ther Adv Chronic Dis. (2022) 13:20406223221076890. doi: 10.1177/20406223221076890, PMID: 35198136 PMC8859684

[40] Taquet M Sillett R Zhu L Mendel J Camplisson I Dercon Q. Neurological and psychiatric risk trajectories after SARS-CoV-2 infection: an analysis of 2-year retrospective cohort studies including 1 284 437 patients. Lancet Psychiatry. (2022) 9:815–27. doi: 10.1016/S2215-0366(22)00260-7, PMID: 35987197 PMC9385200

