# Supplementary material for: Prevalence and severity of neurologic symptoms in Long-COVID and the role of pre-existing conditions, hospitalization, and mental health
Source: Front Neurol. 2025 Jun 25;16:1562084. doi: 10.3389/fneur.2025.1562084 (PMC12237652; doi:10.3389/fneur.2025.1562084)
Supplement: Supplementary Figure 1 — Prisma diagram for participant selection including reasoning for exclusions. [file Supplementary_file_1.docx]

Supplemental Figure 1: PRISMA Digram for Participant Selection and Exclusion

Participants removed *before screening* for no response, not eligible, or no longer interested:

(n = 422)

Participants identified from Inquiries:

(n = 872)

**Identification**

Participants excluded for not completing required questionnaires:

(n = 150)

Participants screened

(n = 426)

Participants excluded for not having a documented positive COVID test:

(n = 38)

Participants screened

(n = 276)

**Screening**

Participants excluded for full recovery:

(n = 23)

Participants screened

(n = 238)

Participants excluded for being within 30 days of infection:

(n = 2)

Participants screened

(n = 215)

Participants included in study

(n = 213)

**Included**
