# Supplementary material for: Prevalence and severity of neurologic symptoms in Long-COVID and the role of pre-existing conditions, hospitalization, and mental health
Source: Front Neurol. 2025 Jun 25;16:1562084. doi: 10.3389/fneur.2025.1562084 (PMC12237652; doi:10.3389/fneur.2025.1562084)
Supplement: Supplementary Figure 2 — Questionnaires used to provide data for this study. [file Supplementary_file_2.pdf]

Demographics Module

Demographics

This set of questions is to identify basic information about you and your household. We are asking this information to identify trends in who is being infected with COVID-19, as well as to make sure we include people from all backgrounds and ethnicities when we do this research study. This will help us make sure we study all of the American experiences of recovery from COVID-19 infections.

Please answer these questions to the best of your ability. You may choose not to answer a sensitive question.

Sex Identified at Birth:	○ Male<br>○ Female<br>○ Other	If other, state sex identified at birth:	
Gender	○ Male<br>○ Female<br>○ Different Identity	If different identity, please describe:	
Race (Select all that apply):	☐ White<br>☐ Black/African American<br>☐ Asian American<br>☐ American Indian or Alaska Native<br>☐ Native Hawaiian or other Pacific Islander<br>☐ Race not represented<br>☐ Unknown Race<br>☐ Other	If other race, please describe:	
Ethnicity:		○ Hispanic<br>○ Non-Hispanic	
Please choose the highest education completed:		○ Never attended school<br>○ Grades 1- 11<br>○ 12th grade, no diploma<br>○ GED or equivalent<br>○ High school graduate<br>○ Some college, no degree<br>○ Associate degree: Occupational, Technical or Vocational program<br>○ Associate degree: Academic<br>○ Bachelor's degree<br>○ Master's degree<br>○ Professional school (MD, DMD)<br>○ Doctoral degree	

The next questions are about information on your health insurance. Your insurance status does not affect your ability to participate, and you will not be billed to participate.

Please let us know about all of your insurance coverage, including health insurance obtained through employment, purchased directly, as well as government programs like Medicare, Medicaid, and the Children's Health Insurance Program that provide medical care or help pay medical bills.

What kinds of health insurance or health care coverage do you have? Select all options which apply:	☐ Private Health Insurance<br>☐ Medicare<br>☐ Medigap<br>☐ Medicaid<br>☐ Children's Health Insurance Program (CHIP)<br>☐ Military related health care: (TRICARE (CHAMPUS)/VA Health/CHAMP VA)<br>☐ Indian Health Service<br>☐ State sponsored health plan<br>☐ Other government program<br>☐ No coverage of any kind

What was your legal marital status before the COVID-19 pandemic?	○ Single<br>○ Living with Partner but unmarried<br>○ Married<br>○ Separated<br>○ Divorced<br>○ Widowed
Did your marital status change during the course of the COVID-19 pandemic?	○ Yes<br>○ No
What is your current legal marital status?	○ Single<br>○ Living with Partner but unmarried<br>○ Married<br>○ Separated<br>○ Divorced<br>○ Widowed
What was your employment status before the COVID-19 pandemic? (Select all that apply): ☐ Full time (40 hr/week)<br>☐ Part-time (less than 40hr/week)<br>☐ Self-employed<br>☐ Full time student<br>☐ Part time student<br>☐ Unemployed<br>☐ Disabled<br>☐ Stay at home parent<br>☐ Retired<br>☐ Other<br>☐ Unsure	If other, then please state employment status before the pandemic:
If you were not working before the COVID-19 pandemic, was it because you were ill?	○ Yes<br>○ No
When was the last date that you were able to work full time?<br><br>*Click on the calendar, and click on the date. Use the slide to indicate the time. If you don't know the time, then leave it blank.*	Format: YYYY-MM-DD HH:MM
Which of these best describes your situation before the COVID-19 pandemic?	○ Cannot perform well in any job, but able to care for myself<br>○ Largely homebound or shut in, but able to care for myself at home<br>○ Largely home bound or shut in<br>○ Occasional attendant care needed for self care<br>○ Largely bed bound, require attendant care
Were you receiving disability benefits before the COVID-19 pandemic?	○ Yes<br>○ No
If you are receiving disability benefits are you on: ○ Social Security Disability Insurance<br>○ Supplemental Security Income from Social Security<br>○ Private disability insurance<br>○ Other	If other, specify disability benefits:
On average, approximately how many hours/week did you work/go to school prior to the pandemic?	

The following list includes groups of job categories. Please choose the job Category/Categories that most closely aligned with your job **prior to the COVID-19 pandemic**. For example, if you worked as a teacher or were a student, you would choose Education, Training and Library. If you worked as a manager at a restaurant, you would choose Management and Food Preparation and Serving Related. If you are not currently working, then choose other and specify "Not working" in the box below (Select all that apply):	☐ Management<br>☐ Business and Financial Operations<br>☐ Computer and Mathematical<br>☐ Architecture and Engineering<br>☐ Life, Physical, and Social Science<br>☐ Community and Social Service<br>☐ Legal Occupations<br>☐ Education, Training, and Library<br>☐ Arts/Design/Entertainment/Sports/Media<br>☐ Healthcare Practitioners and Technical<br>☐ Healthcare Support Occupations<br>☐ Protective Service Occupations<br>☐ Food Preparation and Serving Related<br>☐ Building/Grounds Cleaning & Maintenance<br>☐ Personal Care and Service Occupations<br>☐ Sales and Related Occupations<br>☐ Office and Administrative Support<br>☐ Farming, Fishing, and Forestry<br>☐ Construction and Extraction<br>☐ Installation, Maintenance, and Repair<br>☐ Production Occupations<br>☐ Transportation and Material Moving<br>☐ Military Specific Occupations<br>☐ Other
If other, please specify:	
Did anything about your job change because of the COVID-19 pandemic? This could be the type of job, the number of hours, or your level of responsibility in the job.	○ Yes<br>○ No

If Yes, choose the option that best applies to your current job status:	○ I am no longer working at a job I had prior to the COVID-19 pandemic<br>○ I am not working in any job<br>○ I was a student, but am no longer attending classes due to financial and/orother hardship<br>○ I am no longer working in the job I was in prior to the pandemic, but have a new job<br>○ I am now attending classes as a student but was not attending classes prior to the pandemic<br>○ Other	If other, please describe:	
Current employment status (Select all that apply):	☐ Full time (40 hr/week)<br>☐ Part-time (less than 40hr/week)<br>☐ Self-employed<br>☐ Full time student<br>☐ Part time student<br>☐ Unemployed<br>☐ Disabled<br>☐ Retired<br>☐ Stay at home parent<br>☐ Other	If other, please describe:	
How many hours/week do you currently work?			

The following list includes groups of job categories. Please choose the job Category/Categories that most closely aligned with your job **since the COVID-19 pandemic started**. For example, if you worked as a teacher or were a student, you would choose Education, Training and Library. If you worked as a manager at a restaurant, you would choose Management and Food Preparation and Serving Related. If you are not currently working, then choose other and specify "Not working" in the box below (Select all that apply):	☐ Management<br>☐ Business and Financial Operations<br>☐ Computer and Mathematical<br>☐ Architecture and Engineering<br>☐ Life, Physical, and Social Science<br>☐ Community and Social Service<br>☐ Legal Occupations<br>☐ Education, Training, and Library<br>☐ Arts/Design/Entertainment/Sports/Media<br>☐ Healthcare Practitioners and Technical<br>☐ Healthcare Support Occupations<br>☐ Protective Service Occupations<br>☐ Food Preparation and Serving Related<br>☐ Building/Grounds Cleaning & Maintenance<br>☐ Personal Care and Service Occupations<br>☐ Sales and Related Occupations<br>☐ Office and Administrative Support<br>☐ Farming, Fishing, and Forestry<br>☐ Construction and Extraction<br>☐ Installation, Maintenance, and Repair<br>☐ Production Occupations<br>☐ Transportation and Material Moving<br>☐ Military Specific Occupations<br>☐ Other
If other, please specify:	
Please identify your current living arrangement.	○ I live with family<br>○ I live alone<br>○ I live with a roommate/friend<br>○ I am homeless<br>○ I live in a shelter or treatment facility<br>○ I live within the justice system (prison, jail, court ordered facility)<br>○ Other<br>○ Unknown

We would like to gather information on your household. A household is considered all individuals whom live in one house most days of the week. If the youngest person in the household is a baby under the age of 1, then enter "1" as the age of the youngest person in the household.

Number of individuals living in household:	
Age of oldest individual in household:	
Age of youngest individual in household:	

The following question is optional. By answering this question, it will provide additional information to researchers about whether income level is associated with other measures of COVID-19 infection, such as severity of disease.

What was your household income last year (in 2019) from all people in the house, from all sources before taxes? This includes all income from both formal and informal employment. Answers show both monthly and yearly incomes. The monthly and yearly numbers add up to be the same. (Choose one)	○ Monthly income: $0 to $833 (Yearly income: $0 to $9,999)<br>○ Monthly income: $834 to $1,250 (Yearly income: $10,000 to $14,499)<br>○ Monthly income: $1,251 to $2,082 (Yearly income: $15,000 to $24,999)<br>○ Monthly income: $2,083 to $2,916 (Yearly income: $25,000 to $34,999)<br>○ Monthly income: $2,917 to $4,167 (Yearly income: $35,000 to $49,999)<br>○ Monthly income: $4,168 to $6,249 (Yearly income: $50,000 to $74,999)<br>○ Monthly income: $6,250 or more (Yearly income: $75,000 or more)<br>○ Don't Know<br>○ Refuse to Answer

Before COVID-19 Infection

BEFORE COVID-19 INFECTION

The next set of questions are to help us better understand how you were feeling before your recent COVID-19 infection. Some questions will ask about specific medical issues BEFORE you became ill with COVID -19. If you are not sure what a question means or it does not make sense to you, please mark "Unsure".

Past Medical History

Before your recent COVID-19 infection, have you ever been told by a doctor or other health professional that you have:

*Human Immunodeficiency Virus (HIV) or Acquired Immunodeficiency Syndrome (AIDS):	○ Yes<br>○ No<br>○ Unsure		
*Hepatitis B:	○ Yes<br>○ No<br>○ Unsure		
*Hepatitis C:	○ Yes<br>○ No<br>○ Unsure		
*Tuberculosis:	○ Yes<br>○ No<br>○ Unsure		
*Herpes/Shingles:	○ Yes<br>○ No<br>○ Unsure		
*Other chronic infections that are not listed above?	○ Yes<br>○ No<br>○ Unsure	If yes or unsure, describe the infection and when you had it:	
Has a doctor or other health professional told you that you had pneumonia or a serious lung infection before your recent COVID-19 infection?	○ Yes<br>○ No<br>○ Unsure	If yes or unsure, describe the infection and when you had it:	
Have you ever had an infection that led to a prolonged or very long recovery before your recent COVID-19 infection?	○ Yes<br>○ No<br>○ Unsure	If yes or unsure, please explain:	
*Coronary heart disease (Examples include angina, chest pain, heart attack, myocardial infarction)?	○ Yes<br>○ No<br>○ Unsure		
*Congestive heart failure?	○ Yes<br>○ No<br>○ Unsure		
*An irregular heartbeat (heart arrhythmia, atrial fibrillation)?	○ Yes<br>○ No<br>○ Unsure		
*High blood pressure (hypertension)?	○ Yes<br>○ No<br>○ Unsure		
*High cholesterol?	○ Yes<br>○ No<br>○ Unsure		

*Chronic kidney disease?	Yes<br>No<br>Unsure	If yes, do you need dialysis?	Yes<br>No<br>Unsure
*Seasonal or environmental allergies?		Yes<br>No<br>Unsure	
*Any allergies that would cause you to have difficulty breathing?	Yes<br>No<br>Unsure	If yes, describe:	
*Asthma?		Yes<br>No<br>Unsure	
*Chronic bronchitis, emphysema, or Chronic Obstructive Pulmonary Disease (COPD)?		Yes<br>No<br>Unsure	
*Pulmonary fibrosis?		Yes<br>No<br>Unsure	
*Stroke or a transient ischemic event?		Yes<br>No<br>Unsure	
*Seizure or epilepsy?		Yes<br>No<br>Unsure	
*Multiple sclerosis?		Yes<br>No<br>Unsure	
*Parkinson's disease?		Yes<br>No<br>Unsure	
*Guillain-Barre syndrome?		Yes<br>No<br>Unsure	
*Myasthenia Gravis?		Yes<br>No<br>Unsure	
*Dementia, Vascular dementia or Alzheimer's Disease?		Yes<br>No<br>Unsure	
*Rheumatoid arthritis?		Yes<br>No<br>Unsure	
*Arthritis or Osteoarthritis?		Yes<br>No<br>Unsure	
*Gout?		Yes<br>No<br>Unsure	
*Systemic lupus erythematosus (lupus)?		Yes<br>No<br>Unsure	

*Thyroid Disease (Examples include hyperthyroidism, hypothyroidism, autoimmune thyroid disease, Graves' disease, Hashimoto's thyroiditis)?	○ Yes<br>○ No<br>○ Unsure
*Psoriasis or psoriatic arthritis?	○ Yes<br>○ No<br>○ Unsure
*Vasculitis?	○ Yes<br>○ No<br>○ Unsure
*Fibromyalgia?	○ Yes<br>○ No<br>○ Unsure
*Ehlers-Danlos Syndrome or Benign Hypermobility?	○ Yes<br>○ No<br>○ Unsure
*Myalgic Encephalomyelitis/Chronic Fatigue Syndrome?	○ Yes<br>○ No<br>○ Unsure
*Gastrointestinal disease (Examples include stomach ulcers or Gastroesophageal Reflux Disease (GERD))?	○ Yes<br>○ No<br>○ Unsure
*Liver problems (Examples include hepatitis, fatty liver, or cirrhosis)? ○ Yes ○ No ○ Unsure	If yes, what kind?
*Pancreas problems (An example includes pancreatitis)? ○ Yes ○ No ○ Unsure	If yes, what kind?
*Inflammatory bowel disease (Examples include Crohn's Disease, Ulcerative Colitis)? ○ Yes ○ No ○ Unsure	If yes, what kind?
*Irritable bowel syndrome?	○ Yes<br>○ No<br>○ Unsure
*Rashes, eczema, or dermatitis?	○ Yes<br>○ No<br>○ Unsure
*Eye redness, itching, or pain?	○ Yes<br>○ No<br>○ Unsure
*Prediabetes, borderline diabetes, or gestational diabetes?	○ Yes<br>○ No<br>○ Unsure
*Type 1 Diabetes?	○ Yes<br>○ No<br>○ Unsure
*Type 2 Diabetes?	○ Yes<br>○ No<br>○ Unsure
*Blood clot, deep venous thrombosis, or pulmonary embolism?	○ Yes<br>○ No<br>○ Unsure

*Cancer or a malignancy?	Yes<br>No<br>Unsure	If yes, please enter what kind(s) and a brief note about how each kind was treated (Treatments could include surgery, chemotherapy, radiation, biologic therapy, immunotherapy, or other).	
*Anxiety (Examples of anxiety disorders include generalized anxiety disorder, social anxiety disorder, panic disorder, post-traumatic stress disorder, obsessive-compulsive disorder, and phobias)?		Yes<br>No<br>Unsure	
*Depression (Examples include major depression, major depressive disorder, bipolar depression, dysthymia, post-partum depression, and seasonal affective disorder)?		Yes<br>No<br>Unsure	
*Psychotic disorder (Examples include bipolar illness with psychosis, schizophrenia)?		Yes<br>No<br>Unsure	
*Before you became ill with COVID-19, did you have any other disease, illness, or condition(s) not mentioned in the list above that was being treated by healthcare professionals?	Yes<br>No<br>Unsure	If yes or unsure, please list and describe other health issues. Also, if you stated unsure to any questions above, please explain.	

Pre-COVID-19 Review of Systems

In the three months before your recent COVID-19 infection, did you have any of the following health problems or symptoms.

Symptom	Did you have this symptom during the three months before your recent COVID-19 infection?	How much did it bother you in the three months before your recent COVID-19 infection?
Fever of >100.4	Yes<br>No<br>Unsure	Not at all<br>Mild amount<br>Moderate amount<br>Severe amount<br>Worst possible amount
Subjective fever (felt feverish but did not measure a temperature)	Yes<br>No<br>Unsure	Not at all<br>Mild amount<br>Moderate amount<br>Severe amount<br>Worst possible amount
Chills	Yes<br>No<br>Unsure	Not at all<br>Mild amount<br>Moderate amount<br>Severe amount<br>Worst possible amount
Repeated shaking with chills	Yes<br>No<br>Unsure	Not at all<br>Mild amount<br>Moderate amount<br>Severe amount<br>Worst possible amount
Runny nose	Yes<br>No<br>Unsure	Not at all<br>Mild amount<br>Moderate amount<br>Severe amount<br>Worst possible amount
Sore throat	Yes<br>No<br>Unsure	Not at all<br>Mild amount<br>Moderate amount

		Severe amount<br>Worst possible amount
"Flu-like" symptoms	Yes<br>No<br>Unsure	Not at all<br>Mild amount<br>Moderate amount<br>Severe amount<br>Worst possible amount
**Symptom**	**Did you have this symptom during the three months before your recent COVID-19 infection?**	**How much did it bother you in the three months before your recent COVID-19 infection?**
Cold hands and feet	Yes<br>No<br>Unsure	Not at all<br>Mild amount<br>Moderate amount<br>Severe amount<br>Worst possible amount
Swollen or tender glands (lymph nodes)	Yes<br>No<br>Unsure	Not at all<br>Mild amount<br>Moderate amount<br>Severe amount<br>Worst possible amount
Cough	Yes<br>No<br>Unsure	Not at all<br>Mild amount<br>Moderate amount<br>Severe amount<br>Worst possible amount
Shortness of breath at rest	Yes<br>No<br>Unsure	Not at all<br>Mild amount<br>Moderate amount<br>Severe amount<br>Worst possible amount
Shortness of breath when moving	Yes<br>No<br>Unsure	Not at all<br>Mild amount<br>Moderate amount<br>Severe amount<br>Worst possible amount
Wheezing (makes noise when breathing out)	Yes<br>No<br>Unsure	Not at all<br>Mild amount<br>Moderate amount<br>Severe amount<br>Worst possible amount
**Symptom**	**Did you have this symptom during the three months before your recent COVID-19 infection?**	**How much did it bother you in the three months before your recent COVID-19 infection?**
Chest pain when taking a deep breath	Yes<br>No<br>Unsure	Not at all<br>Mild amount<br>Moderate amount<br>Severe amount<br>Worst possible amount
Discomfort, pain, tightness, or pressure in the chest	Yes<br>No<br>Unsure	Not at all<br>Mild amount<br>Moderate amount<br>Severe amount<br>Worst possible amount
Increased heart rate, fluttering in the chest, or palpitations	Yes<br>No	Not at all<br>Mild amount

	Unsure	Moderate amount<br>Severe amount<br>Worst possible amount
Pain in your legs (cramps) after walking a distance	Yes<br>No<br>Unsure	Not at all<br>Mild amount<br>Moderate amount<br>Severe amount<br>Worst possible amount
Ankles swelling	Yes<br>No<br>Unsure	Not at all<br>Mild amount<br>Moderate amount<br>Severe amount<br>Worst possible amount
Fatigue	Yes<br>No<br>Unsure	Not at all<br>Mild amount<br>Moderate amount<br>Severe amount<br>Worst possible amount
**Symptom**	**Did you have this symptom during the three months before your recent COVID-19 infection?**	**How much did it bother you in the three months before your recent COVID-19 infection?**
General lack of energy or malaise	Yes<br>No<br>Unsure	Not at all<br>Mild amount<br>Moderate amount<br>Severe amount<br>Worst possible amount
Difficulty tolerating being very hot or very cold	Yes<br>No<br>Unsure	Not at all<br>Mild amount<br>Moderate amount<br>Severe amount<br>Worst possible amount
Hot flushes, sweating episodes	Yes<br>No<br>Unsure	Not at all<br>Mild amount<br>Moderate amount<br>Severe amount<br>Worst possible amount
Feeling like it was difficult to stay awake	Yes<br>No<br>Unsure	Not at all<br>Mild amount<br>Moderate amount<br>Severe amount<br>Worst possible amount
Trouble sleeping	Yes<br>No<br>Unsure	Not at all<br>Mild amount<br>Moderate amount<br>Severe amount<br>Worst possible amount
Unrefreshed sleep or not feeling well rested after sleeping	Yes<br>No<br>Unsure	Not at all<br>Mild amount<br>Moderate amount<br>Severe amount<br>Worst possible amount
**Symptom**	**Did you have this symptom during the three months before your recent COVID-19 infection?**	**How much did it bother you in the three months before your recent COVID-19 infection?**

Need for several pillows or to sit in a chair to sleep comfortably	Yes<br>No<br>Unsure	Not at all<br>Mild amount<br>Moderate amount<br>Severe amount<br>Worst possible amount
Nausea or vomiting	Yes<br>No<br>Unsure	Not at all<br>Mild amount<br>Moderate amount<br>Severe amount<br>Worst possible amount
Loss of appetite	Yes<br>No<br>Unsure	Not at all<br>Mild amount<br>Moderate amount<br>Severe amount<br>Worst possible amount
Stomach or abdominal pain	Yes<br>No<br>Unsure	Not at all<br>Mild amount<br>Moderate amount<br>Severe amount<br>Worst possible amount
Heartburn	Yes<br>No<br>Unsure	Not at all<br>Mild amount<br>Moderate amount<br>Severe amount<br>Worst possible amount
Constipation	Yes<br>No<br>Unsure	Not at all<br>Mild amount<br>Moderate amount<br>Severe amount<br>Worst possible amount
**Symptom**	**Did you have this symptom during the three months before your recent COVID-19 infection?**	**How much did it bother you in the three months before your recent COVID-19 infection?**
Diarrhea (3 or more looser than normal stools in a 24-hour period)	Yes<br>No<br>Unsure	Not at all<br>Mild amount<br>Moderate amount<br>Severe amount<br>Worst possible amount
Headache	Yes<br>No<br>Unsure	Not at all<br>Mild amount<br>Moderate amount<br>Severe amount<br>Worst possible amount
Muscle aches or pains	Yes<br>No<br>Unsure	Not at all<br>Mild amount<br>Moderate amount<br>Severe amount<br>Worst possible amount
Joint aches or pains	Yes<br>No<br>Unsure	Not at all<br>Mild amount<br>Moderate amount<br>Severe amount<br>Worst possible amount

Numbness or tingling in your face, arm, or leg	Yes<br>No<br>Unsure	Not at all<br>Mild amount<br>Moderate amount<br>Severe amount<br>Worst possible amount
Weakness in your face, arm, or leg	Yes<br>No<br>Unsure	Not at all<br>Mild amount<br>Moderate amount<br>Severe amount<br>Worst possible amount
**Symptom**	**Did you have this symptom during the three months before your recent COVID-19 infection?**	**How much did it bother you in the three months before your recent COVID-19 infection?**
Feeling generally weak	Yes<br>No<br>Unsure	Not at all<br>Mild amount<br>Moderate amount<br>Severe amount<br>Worst possible amount
Felt delirious (not in your right mind)	Yes<br>No<br>Unsure	Not at all<br>Mild amount<br>Moderate amount<br>Severe amount<br>Worst possible amount
Had hallucinations (saw or heard things that were not there)	Yes<br>No<br>Unsure	Not at all<br>Mild amount<br>Moderate amount<br>Severe amount<br>Worst possible amount
Difficulty concentrating or remembering things	Yes<br>No<br>Unsure	Not at all<br>Mild amount<br>Moderate amount<br>Severe amount<br>Worst possible amount
Dizziness or lightheadedness	Yes<br>No<br>Unsure	Not at all<br>Mild amount<br>Moderate amount<br>Severe amount<br>Worst possible amount
Difficulty with speaking or finding words	Yes<br>No<br>Unsure	Not at all<br>Mild amount<br>Moderate amount<br>Severe amount<br>Worst possible amount
**Symptom**	**Did you have this symptom during the three months before your recent COVID-19 infection?**	**How much did it bother you in the three months before your recent COVID-19 infection?**
Difficulty with balance or coordination	Yes<br>No<br>Unsure	Not at all<br>Mild amount<br>Moderate amount<br>Severe amount<br>Worst possible amount
Difficulty understanding words	Yes<br>No<br>Unsure	Not at all<br>Mild amount<br>Moderate amount

		Severe amount<br>Worst possible amount
Sensitive to food	Yes<br>No<br>Unsure	Not at all<br>Mild amount<br>Moderate amount<br>Severe amount<br>Worst possible amount
Sensitive to chemicals	Yes<br>No<br>Unsure	Not at all<br>Mild amount<br>Moderate amount<br>Severe amount<br>Worst possible amount
Sensitive to light	Yes<br>No<br>Unsure	Not at all<br>Mild amount<br>Moderate amount<br>Severe amount<br>Worst possible amount
Sensitive to noise	Yes<br>No<br>Unsure	Not at all<br>Mild amount<br>Moderate amount<br>Severe amount<br>Worst possible amount
**Symptom**	**Did you have this symptom during the three months <u>before</u> your recent COVID-19 infection?**	**How much did it bother you in the three months <u>before</u> your recent COVID-19 infection?**
Sensitive to touch	Yes<br>No<br>Unsure	Not at all<br>Mild amount<br>Moderate amount<br>Severe amount<br>Worst possible amount
Painful toes with change in color	Yes<br>No<br>Unsure	Not at all<br>Mild amount<br>Moderate amount<br>Severe amount<br>Worst possible amount
Sores or ulcers in your mouth	Yes<br>No<br>Unsure	Not at all<br>Mild amount<br>Moderate amount<br>Severe amount<br>Worst possible amount
Skin rash	Yes<br>No<br>Unsure	Not at all<br>Mild amount<br>Moderate amount<br>Severe amount<br>Worst possible amount
Hair loss	Yes<br>No<br>Unsure	Not at all<br>Mild amount<br>Moderate amount<br>Severe amount<br>Worst possible amount
Eye discharge	Yes<br>No<br>Unsure	Not at all<br>Mild amount<br>Moderate amount

		Severe amount<br>Worst possible amount
**Symptom**	**Did you have this symptom during the three months before your recent COVID-19 infection?**	**How much did it bother you in the three months before your recent COVID-19 infection?**
Eye redness	Yes<br>No<br>Unsure	Not at all<br>Mild amount<br>Moderate amount<br>Severe amount<br>Worst possible amount
Feeling of something in your eye	Yes<br>No<br>Unsure	Not at all<br>Mild amount<br>Moderate amount<br>Severe amount<br>Worst possible amount
Itching of the eyes	Yes<br>No<br>Unsure	Not at all<br>Mild amount<br>Moderate amount<br>Severe amount<br>Worst possible amount
Eye pain	Yes<br>No<br>Unsure	Not at all<br>Mild amount<br>Moderate amount<br>Severe amount<br>Worst possible amount
Blurred vision	Yes<br>No<br>Unsure	Not at all<br>Mild amount<br>Moderate amount<br>Severe amount<br>Worst possible amount
See specks or flashes of light	Yes<br>No<br>Unsure	Not at all<br>Mild amount<br>Moderate amount<br>Severe amount<br>Worst possible amount
Dry mouth	Yes<br>No<br>Unsure	Not at all<br>Mild amount<br>Moderate amount<br>Severe amount<br>Worst possible amount

The next set of questions are to understand if you have any difficulties with walking.

Did you have difficulty walking or climbing steps?

- No difficulty
- Some difficulty
- A lot of difficulty
- Cannot do at all
- Unsure

Did you have difficulty walking 100 yards on level ground (a football field or one city block)?

- No difficulty
- Some difficulty
- A lot of difficulty
- Cannot do at all
- Unsure

Did you have difficulty walking a third of a mile on level ground (five football fields or five city blocks)?

- No difficulty
- Some difficulty
- A lot of difficulty
- Cannot do at all
- Unsure

Did you have difficulty walking up or down 12 steps?

- No difficulty
- Some difficulty
- A lot of difficulty
- Cannot do at all
- Unsure

Smell and Taste

The next section is focused on your experience of smell, taste, and food flavor before your recent COVID 19 infection.

*The following questions relate to your **sense of smell** (for example, sniffing flowers or soap, or smelling garbage) but **not the flavor of food** in your mouth.*

Rate your ability to smell BEFORE your recent COVID-19 infection by sliding the bar to the appropriate point:

{0 / 50}

No sense of smell — Excellent sense of smell

Rate how blocked your nose was BEFORE your recent COVID-19 infection by sliding the bar to the appropriate point:

{0 / 50}

Not at all blocked — Completely blocked

*The following question relates to your **sense of taste** (for example, sweetness, sourness, saltiness, bitterness) experienced in your mouth.*

Rate your ability to taste BEFORE your recent COVID-19 infection by sliding the bar to the appropriate point:

{0 / 50}

No sense of taste — Excellent sense of taste

*The following question is related to other sensations in your mouth, like **burning, cooling, or tingling**. For example, chili peppers, mint gum or candy, or carbonation.*

Rate your ability to feel these other sensations like burning, cooling, and tingling BEFORE your recent COVID-19 infection by sliding the bar to the appropriate point:

{0 / 50}

Not sensitive at all — Very sensitive

General health status:

Did you get a flu shot between 9/2019 - 3/2020?	Yes<br>No<br>Unsure	Did you get a flu shot between 9/2020-3/2021?	Yes<br>No<br>Unsure

	◯ NA/Not yet
Before your recent COVID-19 infection, how would you say your health was in general?	◯ Excellent<br>◯ Very Good<br>◯ Good<br>◯ Fair<br>◯ Poor<br>◯ Unsure
What was your height without shoes three months before your COVID-19 infection?	
Feet	Inches
What was your weight in pounds three months before your COVID-19 infection?	
Pounds	Was this an estimate or did you weigh using a scale? ◯ Estimated weight ◯ Scale weight

During COVID-19 Infection

Your Recent Infection Symptoms

The following questions are to help us better understand your symptoms at the time of your recent COVID-19 infection. The questions are about your symptoms, when you had them, how much they bothered you, and how they did or did not improve. If you do not understand a question, you can mark "Unsure".

What was the date you first had a symptom and suspected you may be infected with COVID-19?

Click on the calendar below, and click on the date. Use the slide to indicate the time. If you don't know the time, then leave it blank.

Format: YYYY-MM-DD HH:MM

Symptom	Did you have this symptom when you were recently infected?	When did this symptom begin?	How many days after your infection started did the symptom start?	When the symptom was at its worst, how much did it bother you?	How did this symptom end?
**Fever of >100.4**	Yes<br>No<br>Unsure	I had this symptom before my infection started<br>This was one of the initial symptoms I had<br>The symptom started after my infection started<br>Unsure		Not at all<br>Mild amount<br>Moderate amount<br>Severe amount<br>Worst possible amount	It went away when my infection went away<br>It went away but the symptom lingered on for a while after my other symptoms went away. I do not have the symptom now<br>It went away for a while and then it returned. I do not have the symptom now<br>It went away for a while and then it returned. I have the symptom now<br>It did not go away and I still have the symptom now<br>I had it before my infection started. It has returned to normal<br>I had it before my infection started. It is worse than before I was infected
**Subjective fever (felt feverish but did not measure a temperature)**	Yes<br>No<br>Unsure	I had this symptom before my infection started<br>This was one of the initial symptoms I had<br>The symptom started after my infection started<br>Unsure		Not at all<br>Mild amount<br>Moderate amount<br>Severe amount<br>Worst possible amount	It went away when my infection went away<br>It went away but the symptom lingered on for a while after my other symptoms went away. I do not have the symptom now<br>It went away for a while and then it returned. I do not have the symptom now<br>It went away for a while and then it returned. I have the symptom now<br>It did not go away and I still have the symptom now<br>I had it before my infection started. It has returned to normal<br>I had it before my infection started. It is worse than before I was infected
**Chills**	Yes<br>No<br>Unsure	I had this symptom before my infection started<br>This was one of the initial symptoms I had<br>The symptom started after my infection started<br>[illegible]		Not at all<br>Mild amount<br>Moderate amount<br>Severe amount<br>Worst possible amount	It went away when my infection went away<br>It went away but the symptom lingered on for a while after my other symptoms went away. I do not have the symptom now

		Unsure			It went away for a while and then it returned. I do not have the symptom now<br>It went away for a while and then it returned. I have the symptom now<br>It did not go away and I still have the symptom now<br>I had it before my infection started. It has returned to normal<br>I had it before my infection started. It is worse than before I was infected
**Symptom**	**Did you have this symptom when you were recently infected?**	**When did this symptom begin?**	**How many days after your infection started did the symptom start?**	**When the symptom was at its worst, how much did it bother you?**	**How did this symptom end?**
**Repeated shaking with chills**	Yes<br>No<br>Unsure	I had this symptom before my infection started<br>This was one of the initial symptoms I had<br>The symptom started after my infection started<br>Unsure		Not at all<br>Mild amount<br>Moderate amount<br>Severe amount<br>Worst possible amount	It went away when my infection went away<br>It went away but the symptom lingered on for a while after my other symptoms went away. I do not have the symptom now<br>It went away for a while and then it returned. I do not have the symptom now<br>It went away for a while and then it returned. I have the symptom now<br>It did not go away and I still have the symptom now<br>I had it before my infection started. It has returned to normal<br>I had it before my infection started. It is worse than before I was infected
**Runny nose**	Yes<br>No<br>Unsure	I had this symptom before my infection started<br>This was one of the initial symptoms I had<br>The symptom started after my infection started<br>Unsure		Not at all<br>Mild amount<br>Moderate amount<br>Severe amount<br>Worst possible amount	It went away when my infection went away<br>It went away but the symptom lingered on for a while after my other symptoms went away. I do not have the symptom now<br>It went away for a while and then it returned. I do not have the symptom now<br>It went away for a while and then it returned. I have the symptom now<br>It did not go away and I still have the symptom now<br>I had it before my infection started. It has returned to normal<br>I had it before my infection started. It is worse than before I was infected
**Sore throat**	Yes<br>No<br>Unsure	I had this symptom before my infection started<br>This was one of the initial symptoms I had<br>The symptom started after my infection started<br>[illegible]		Not at all<br>Mild amount<br>Moderate amount<br>Severe amount<br>Worst possible amount	It went away when my infection went away<br>It went away but the symptom lingered on for a while after my other symptoms went away. I do not have the symptom now

		Unsure			It went away for a while and then it returned. I do not have the symptom now<br>It went away for a while and then it returned. I have the symptom now<br>It did not go away and I still have the symptom now<br>I had it before my infection started. It has returned to normal<br>I had it before my infection started. It is worse than before I was infected
**Symptom**	**Did you have this symptom when you were recently infected?**	**When did this symptom begin?**	**How many days after your infection started did the symptom start?**	**When the symptom was at its worst, how much did it bother you?**	**How did this symptom end?**
**"Flu-like" symptoms**	Yes<br>No<br>Unsure	I had this symptom before my infection started<br>This was one of the initial symptoms I had<br>The symptom started after my infection started<br>Unsure		Not at all<br>Mild amount<br>Moderate amount<br>Severe amount<br>Worst possible amount	It went away when my infection went away<br>It went away but the symptom lingered on for a while after my other symptoms went away. I do not have the symptom now Less<br>It went away for a while and then it returned. I do not have the symptom now<br>It went away for a while and then it returned. I have the symptom now<br>It did not go away and I still have the symptom now<br>I had it before my infection started. It has returned to normal<br>I had it before my infection started. It is worse than before I was infected
**Cold hands and feet**	Yes<br>No<br>Unsure	I had this symptom before my infection started<br>This was one of the initial symptoms I had<br>The symptom started after my infection started<br>Unsure		Not at all<br>Mild amount<br>Moderate amount<br>Severe amount<br>Worst possible amount	It went away when my infection went away<br>It went away but the symptom lingered on for a while after my other symptoms went away. I do not have the symptom now<br>It went away for a while and then it returned. I do not have the symptom now<br>It went away for a while and then it returned. I have the symptom now<br>It did not go away and I still have the symptom now<br>I had it before my infection started. It has returned to normal<br>I had it before my infection started. It is worse than before I was infected
**Swollen or tender glands (lymph nodes)**	Yes<br>No<br>Unsure	I had this symptom before my infection started<br>This was one of the initial symptoms I had<br>The symptom started after my infection started<br>Unsure		Not at all<br>Mild amount<br>Moderate amount<br>Severe amount<br>Worst possible amount	It went away when my infection went away<br>It went away but the symptom lingered on for a while after my other symptoms went away. I do not have the symptom now

		Unsure			It went away for a while and then it returned. I do not have the symptom now<br>It went away for a while and then it returned. I have the symptom now<br>It did not go away and I still have the symptom now<br>I had it before my infection started. It has returned to normal<br>I had it before my infection started. It is worse than before I was infected
**Symptom**	**Did you have this symptom when you were recently infected?**	**When did this symptom begin?**	**How many days after your infection started did the symptom start?**	**When the symptom was at its worst, how much did it bother you?**	**How did this symptom end?**
**Cough**	Yes<br>No<br>Unsure	I had this symptom before my infection started<br>This was one of the initial symptoms I had<br>The symptom started after my infection started<br>Unsure		Not at all<br>Mild amount<br>Moderate amount<br>Severe amount<br>Worst possible amount	It went away when my infection went away<br>It went away but the symptom lingered on for a while after my other symptoms went away. I do not have the symptom now<br>It went away for a while and then it returned. I do not have the symptom now<br>It went away for a while and then it returned. I have the symptom now<br>It did not go away and I still have the symptom now<br>I had it before my infection started. It has returned to normal<br>I had it before my infection started. It is worse than before I was infected
**Shortness of breath at rest**	Yes<br>No<br>Unsure	I had this symptom before my infection started<br>This was one of the initial symptoms I had<br>The symptom started after my infection started<br>Unsure		Not at all<br>Mild amount<br>Moderate amount<br>Severe amount<br>Worst possible amount	It went away when my infection went away<br>It went away but the symptom lingered on for a while after my other symptoms went away. I do not have the symptom now<br>It went away for a while and then it returned. I do not have the symptom now<br>It went away for a while and then it returned. I have the symptom now<br>It did not go away and I still have the symptom now<br>I had it before my infection started. It has returned to normal<br>I had it before my infection started. It is worse than before I was infected
**Shortness of breath when moving**	Yes<br>No<br>Unsure	I had this symptom before my infection started<br>This was one of the initial symptoms I had<br>The symptom started after my infection started<br>Unsure		Not at all<br>Mild amount<br>Moderate amount<br>Severe amount<br>Worst possible amount	It went away when my infection went away<br>It went away but the symptom lingered on for a while after my other symptoms went away. I do not have the symptom now

		Unsure			It went away for a while and then it returned. I do not have the symptom now<br>It went away for a while and then it returned. I have the symptom now<br>It did not go away and I still have the symptom now<br>I had it before my infection started. It has returned to normal<br>I had it before my infection started. It is worse than before I was infected
**Symptom**	**Did you have this symptom when you were recently infected?**	**When did this symptom begin?**	**How many days after your infection started did the symptom start?**	**When the symptom was at its worst, how much did it bother you?**	**How did this symptom end?**
**Wheezing (makes noise when breathing out)**	Yes<br>No<br>Unsure	I had this symptom before my infection started<br>This was one of the initial symptoms I had<br>The symptom started after my infection started<br>Unsure		Not at all<br>Mild amount<br>Moderate amount<br>Severe amount<br>Worst possible amount	It went away when my infection went away<br>It went away but the symptom lingered on for a while after my other symptoms went away. I do not have the symptom now<br>It went away for a while and then it returned. I do not have the symptom now<br>It went away for a while and then it returned. I have the symptom now<br>It did not go away and I still have the symptom now<br>I had it before my infection started. It has returned to normal<br>I had it before my infection started. It is worse than before I was infected
**Chest pain when taking a deep breath**	Yes<br>No<br>Unsure	I had this symptom before my infection started<br>This was one of the initial symptoms I had<br>The symptom started after my infection started<br>Unsure		Not at all<br>Mild amount<br>Moderate amount<br>Severe amount<br>Worst possible amount	It went away when my infection went away<br>It went away but the symptom lingered on for a while after my other symptoms went away. I do not have the symptom now<br>It went away for a while and then it returned. I do not have the symptom now<br>It went away for a while and then it returned. I have the symptom now<br>It did not go away and I still have the symptom now<br>I had it before my infection started. It has returned to normal<br>I had it before my infection started. It is worse than before I was infected
**Discomfort, pain, tightness, or pressure in the chest**	Yes<br>No<br>Unsure	I had this symptom before my infection started<br>This was one of the initial symptoms I had<br>The symptom started after my infection started<br>[illegible]		Not at all<br>Mild amount<br>Moderate amount<br>Severe amount<br>Worst possible amount	It went away when my infection went away<br>It went away but the symptom lingered on for a while after my other symptoms went away. I do not have the symptom now

		Unsure			It went away for a while and then it returned. I do not have the symptom now<br>It went away for a while and then it returned. I have the symptom now<br>It did not go away and I still have the symptom now<br>I had it before my infection started. It has returned to normal<br>I had it before my infection started. It is worse than before I was infected
**Symptom**	**Did you have this symptom when you were recently infected?**	**When did this symptom begin?**	**How many days after your infection started did the symptom start?**	**When the symptom was at its worst, how much did it bother you?**	**How did this symptom end?**
**Increased heart rate, fluttering in the chest, or palpitations**	Yes<br>No<br>Unsure	I had this symptom before my infection started<br>This was one of the initial symptoms I had<br>The symptom started after my infection started<br>Unsure		Not at all<br>Mild amount<br>Moderate amount<br>Severe amount<br>Worst possible amount	It went away when my infection went away<br>It went away but the symptom lingered on for a while after my other symptoms went away. I do not have the symptom now<br>It went away for a while and then it returned. I do not have the symptom now<br>It went away for a while and then it returned. I have the symptom now<br>It did not go away and I still have the symptom now<br>I had it before my infection started. It has returned to normal<br>I had it before my infection started. It is worse than before I was infected
**Pain in your legs (cramps) after walking a distance**	Yes<br>No<br>Unsure	I had this symptom before my infection started<br>This was one of the initial symptoms I had<br>The symptom started after my infection started<br>Unsure		Not at all<br>Mild amount<br>Moderate amount<br>Severe amount<br>Worst possible amount	It went away when my infection went away<br>It went away but the symptom lingered on for a while after my other symptoms went away. I do not have the symptom now<br>It went away for a while and then it returned. I do not have the symptom now<br>It went away for a while and then it returned. I have the symptom now<br>It did not go away and I still have the symptom now<br>I had it before my infection started. It has returned to normal<br>I had it before my infection started. It is worse than before I was infected
**Ankles swelling**	Yes<br>No<br>Unsure	I had this symptom before my infection started<br>This was one of the initial symptoms I had<br>The symptom started after my infection started<br>[illegible]		Not at all<br>Mild amount<br>Moderate amount<br>Severe amount<br>Worst possible amount	It went away when my infection went away<br>It went away but the symptom lingered on for a while after my other symptoms went away. I do not have the symptom now

		Unsure			It went away for a while and then it returned. I do not have the symptom now<br>It went away for a while and then it returned. I have the symptom now<br>It did not go away and I still have the symptom now<br>I had it before my infection started. It has returned to normal<br>I had it before my infection started. It is worse than before I was infected
**Symptom**	**Did you have this symptom when you were recently infected?**	**When did this symptom begin?**	**How many days after your infection started did the symptom start?**	**When the symptom was at its worst, how much did it bother you?**	**How did this symptom end?**
**Fatigue**	Yes<br>No<br>Unsure	I had this symptom before my infection started<br>This was one of the initial symptoms I had<br>The symptom started after my infection started<br>Unsure		Not at all<br>Mild amount<br>Moderate amount<br>Severe amount<br>Worst possible amount	It went away when my infection went away<br>It went away but the symptom lingered on for a while after my other symptoms went away. I do not have the symptom now<br>It went away for a while and then it returned. I do not have the symptom now<br>It went away for a while and then it returned. I have the symptom now<br>It did not go away and I still have the symptom now<br>I had it before my infection started. It has returned to normal<br>I had it before my infection started. It is worse than before I was infected
**General lack of energy or malaise**	Yes<br>No<br>Unsure	I had this symptom before my infection started<br>This was one of the initial symptoms I had<br>The symptom started after my infection started<br>Unsure		Not at all<br>Mild amount<br>Moderate amount<br>Severe amount<br>Worst possible amount	It went away when my infection went away<br>It went away but the symptom lingered on for a while after my other symptoms went away. I do not have the symptom now<br>It went away for a while and then it returned. I do not have the symptom now<br>It went away for a while and then it returned. I have the symptom now<br>It did not go away and I still have the symptom now<br>I had it before my infection started. It has returned to normal<br>I had it before my infection started. It is worse than before I was infected
**Difficulty tolerating being very hot or very cold**	Yes<br>No<br>Unsure	I had this symptom before my infection started<br>This was one of the initial symptoms I had<br>The symptom started after my infection started<br>[illegible]		Not at all<br>Mild amount<br>Moderate amount<br>Severe amount<br>Worst possible amount	It went away when my infection went away<br>It went away but the symptom lingered on for a while after my other symptoms went away. I do not have the symptom now

		Unsure			It went away for a while and then it returned. I do not have the symptom now<br>It went away for a while and then it returned. I have the symptom now<br>It did not go away and I still have the symptom now<br>I had it before my infection started. It has returned to normal<br>I had it before my infection started. It is worse than before I was infected
**Symptom**	**Did you have this symptom when you were recently infected?**	**When did this symptom begin?**	**How many days after your infection started did the symptom start?**	**When the symptom was at its worst, how much did it bother you?**	**How did this symptom end?**
**Hot flushes, sweating episodes**	Yes<br>No<br>Unsure	I had this symptom before my infection started<br>This was one of the initial symptoms I had<br>The symptom started after my infection started<br>Unsure		Not at all<br>Mild amount<br>Moderate amount<br>Severe amount<br>Worst possible amount	It went away when my infection went away<br>It went away but the symptom lingered on for a while after my other symptoms went away. I do not have the symptom now<br>It went away for a while and then it returned. I do not have the symptom now<br>It went away for a while and then it returned. I have the symptom now<br>It did not go away and I still have the symptom now<br>I had it before my infection started. It has returned to normal<br>I had it before my infection started. It is worse than before I was infected
**Feeling like it was difficult to stay awake**	Yes<br>No<br>Unsure	I had this symptom before my infection started<br>This was one of the initial symptoms I had<br>The symptom started after my infection started<br>Unsure		Not at all<br>Mild amount<br>Moderate amount<br>Severe amount<br>Worst possible amount	It went away when my infection went away<br>It went away but the symptom lingered on for a while after my other symptoms went away. I do not have the symptom now<br>It went away for a while and then it returned. I do not have the symptom now<br>It went away for a while and then it returned. I have the symptom now<br>It did not go away and I still have the symptom now<br>I had it before my infection started. It has returned to normal<br>I had it before my infection started. It is worse than before I was infected
**Trouble sleeping**	Yes<br>No<br>Unsure	I had this symptom before my infection started<br>This was one of the initial symptoms I had<br>The symptom started after my infection started<br>Unsure		Not at all<br>Mild amount<br>Moderate amount<br>Severe amount<br>Worst possible amount	It went away when my infection went away<br>It went away but the symptom lingered on for a while after my other symptoms went away. I do not have the symptom now

		Unsure			It went away for a while and then it returned. I do not have the symptom now<br>It went away for a while and then it returned. I have the symptom now<br>It did not go away and I still have the symptom now<br>I had it before my infection started. It has returned to normal<br>I had it before my infection started. It is worse than before I was infected
**Symptom**	**Did you have this symptom when you were recently infected?**	**When did this symptom begin?**	**How many days after your infection started did the symptom start?**	**When the symptom was at its worst, how much did it bother you?**	**How did this symptom end?**
**Unrefreshed sleep or not feeling well rested after sleeping**	Yes<br>No<br>Unsure	I had this symptom before my infection started<br>This was one of the initial symptoms I had<br>The symptom started after my infection started<br>Unsure		Not at all<br>Mild amount<br>Moderate amount<br>Severe amount<br>Worst possible amount	It went away when my infection went away<br>It went away but the symptom lingered on for a while after my other symptoms went away. I do not have the symptom now<br>It went away for a while and then it returned. I do not have the symptom now<br>It went away for a while and then it returned. I have the symptom now<br>It did not go away and I still have the symptom now<br>I had it before my infection started. It has returned to normal<br>I had it before my infection started. It is worse than before I was infected
**Need for several pillows or to sit in a chair to sleep comfortably**	Yes<br>No<br>Unsure	I had this symptom before my infection started<br>This was one of the initial symptoms I had<br>The symptom started after my infection started<br>Unsure		Not at all<br>Mild amount<br>Moderate amount<br>Severe amount<br>Worst possible amount	It went away when my infection went away<br>It went away but the symptom lingered on for a while after my other symptoms went away. I do not have the symptom now<br>It went away for a while and then it returned. I do not have the symptom now<br>It went away for a while and then it returned. I have the symptom now<br>It did not go away and I still have the symptom now<br>I had it before my infection started. It has returned to normal<br>I had it before my infection started. It is worse than before I was infected
**Nausea or vomiting**	Yes<br>No<br>Unsure	I had this symptom before my infection started<br>This was one of the initial symptoms I had<br>The symptom started after my infection started<br>[illegible]		Not at all<br>Mild amount<br>Moderate amount<br>Severe amount<br>Worst possible amount	It went away when my infection went away<br>It went away but the symptom lingered on for a while after my other symptoms went away. I do not have the symptom now

		Unsure			It went away for a while and then it returned. I do not have the symptom now<br>It went away for a while and then it returned. I have the symptom now<br>It did not go away and I still have the symptom now<br>I had it before my infection started. It has returned to normal<br>I had it before my infection started. It is worse than before I was infected
**Symptom**	**Did you have this symptom when you were recently infected?**	**When did this symptom begin?**	**How many days after your infection started did the symptom start?**	**When the symptom was at its worst, how much did it bother you?**	**How did this symptom end?**
**Loss of appetite**	Yes<br>No<br>Unsure	I had this symptom before my infection started<br>This was one of the initial symptoms I had<br>The symptom started after my infection started<br>Unsure		Not at all<br>Mild amount<br>Moderate amount<br>Severe amount<br>Worst possible amount	It went away when my infection went away<br>It went away but the symptom lingered on for a while after my other symptoms went away. I do not have the symptom now<br>It went away for a while and then it returned. I do not have the symptom now<br>It went away for a while and then it returned. I have the symptom now<br>It did not go away and I still have the symptom now<br>I had it before my infection started. It has returned to normal<br>I had it before my infection started. It is worse than before I was infected
**Stomach or abdominal pain**	Yes<br>No<br>Unsure	I had this symptom before my infection started<br>This was one of the initial symptoms I had<br>The symptom started after my infection started<br>Unsure		Not at all<br>Mild amount<br>Moderate amount<br>Severe amount<br>Worst possible amount	It went away when my infection went away<br>It went away but the symptom lingered on for a while after my other symptoms went away. I do not have the symptom now<br>It went away for a while and then it returned. I do not have the symptom now<br>It went away for a while and then it returned. I have the symptom now<br>It did not go away and I still have the symptom now<br>I had it before my infection started. It has returned to normal<br>I had it before my infection started. It is worse than before I was infected
**Heartburn**	Yes<br>No<br>Unsure	I had this symptom before my infection started<br>This was one of the initial symptoms I had<br>The symptom started after my infection started<br>[illegible]		Not at all<br>Mild amount<br>Moderate amount<br>Severe amount<br>Worst possible amount	It went away when my infection went away<br>It went away but the symptom lingered on for a while after my other symptoms went away. I do not have the symptom now

		Unsure			It went away for a while and then it returned. I do not have the symptom now<br>It went away for a while and then it returned. I have the symptom now<br>It did not go away and I still have the symptom now<br>I had it before my infection started. It has returned to normal<br>I had it before my infection started. It is worse than before I was infected
**Symptom**	**Did you have this symptom when you were recently infected?**	**When did this symptom begin?**	**How many days after your infection started did the symptom start?**	**When the symptom was at its worst, how much did it bother you?**	**How did this symptom end?**
**Constipation**	Yes<br>No<br>Unsure	I had this symptom before my infection started<br>This was one of the initial symptoms I had<br>The symptom started after my infection started<br>Unsure		Not at all<br>Mild amount<br>Moderate amount<br>Severe amount<br>Worst possible amount	It went away when my infection went away<br>It went away but the symptom lingered on for a while after my other symptoms went away. I do not have the symptom now<br>It went away for a while and then it returned. I do not have the symptom now<br>It went away for a while and then it returned. I have the symptom now<br>It did not go away and I still have the symptom now<br>I had it before my infection started. It has returned to normal<br>I had it before my infection started. It is worse than before I was infected
**Diarrhea (3 or more looser than normal stools in a 24-hour period)**	Yes<br>No<br>Unsure	I had this symptom before my infection started<br>This was one of the initial symptoms I had<br>The symptom started after my infection started<br>Unsure		Not at all<br>Mild amount<br>Moderate amount<br>Severe amount<br>Worst possible amount	It went away when my infection went away<br>It went away but the symptom lingered on for a while after my other symptoms went away. I do not have the symptom now<br>It went away for a while and then it returned. I do not have the symptom now<br>It went away for a while and then it returned. I have the symptom now<br>It did not go away and I still have the symptom now<br>I had it before my infection started. It has returned to normal<br>I had it before my infection started. It is worse than before I was infected
**Headache**	Yes<br>No<br>Unsure	I had this symptom before my infection started<br>This was one of the initial symptoms I had<br>The symptom started after my infection started<br>[illegible]		Not at all<br>Mild amount<br>Moderate amount<br>Severe amount<br>Worst possible amount	It went away when my infection went away<br>It went away but the symptom lingered on for a while after my other symptoms went away. I do not have the symptom now

		Unsure			It went away for a while and then it returned. I do not have the symptom now<br>It went away for a while and then it returned. I have the symptom now<br>It did not go away and I still have the symptom now<br>I had it before my infection started. It has returned to normal<br>I had it before my infection started. It is worse than before I was infected
**Symptom**	**Did you have this symptom when you were recently infected?**	**When did this symptom begin?**	**How many days after your infection started did the symptom start?**	**When the symptom was at its worst, how much did it bother you?**	**How did this symptom end?**
**Muscle aches or pains**	Yes<br>No<br>Unsure	I had this symptom before my infection started<br>This was one of the initial symptoms I had<br>The symptom started after my infection started<br>Unsure		Not at all<br>Mild amount<br>Moderate amount<br>Severe amount<br>Worst possible amount	It went away when my infection went away<br>It went away but the symptom lingered on for a while after my other symptoms went away. I do not have the symptom now<br>It went away for a while and then it returned. I do not have the symptom now<br>It went away for a while and then it returned. I have the symptom now<br>It did not go away and I still have the symptom now<br>I had it before my infection started. It has returned to normal<br>I had it before my infection started. It is worse than before I was infected
**Joint aches or pains**	Yes<br>No<br>Unsure	I had this symptom before my infection started<br>This was one of the initial symptoms I had<br>The symptom started after my infection started<br>Unsure		Not at all<br>Mild amount<br>Moderate amount<br>Severe amount<br>Worst possible amount	It went away when my infection went away<br>It went away but the symptom lingered on for a while after my other symptoms went away. I do not have the symptom now<br>It went away for a while and then it returned. I do not have the symptom now<br>It went away for a while and then it returned. I have the symptom now<br>It did not go away and I still have the symptom now<br>I had it before my infection started. It has returned to normal<br>I had it before my infection started. It is worse than before I was infected
**Sudden numbness or tingling in your face, arm, or leg**	Yes<br>No<br>Unsure	I had this symptom before my infection started<br>This was one of the initial symptoms I had<br>The symptom started after my infection started<br>[illegible]		Not at all<br>Mild amount<br>Moderate amount<br>Severe amount<br>Worst possible amount	It went away when my infection went away<br>It went away but the symptom lingered on for a while after my other symptoms went away. I do not have the symptom now

		Unsure			It went away for a while and then it returned. I do not have the symptom now<br>It went away for a while and then it returned. I have the symptom now<br>It did not go away and I still have the symptom now<br>I had it before my infection started. It has returned to normal<br>I had it before my infection started. It is worse than before I was infected
**Symptom**	**Did you have this symptom when you were recently infected?**	**When did this symptom begin?**	**How many days after your infection started did the symptom start?**	**When the symptom was at its worst, how much did it bother you?**	**How did this symptom end?**
**Numbness or tingling that was not sudden**	Yes<br>No<br>Unsure	I had this symptom before my infection started<br>This was one of the initial symptoms I had<br>The symptom started after my infection started<br>Unsure		Not at all<br>Mild amount<br>Moderate amount<br>Severe amount<br>Worst possible amount	It went away when my infection went away<br>It went away but the symptom lingered on for a while after my other symptoms went away. I do not have the symptom now<br>It went away for a while and then it returned. I do not have the symptom now<br>It went away for a while and then it returned. I have the symptom now<br>It did not go away and I still have the symptom now<br>I had it before my infection started. It has returned to normal<br>I had it before my infection started. It is worse than before I was infected
**Sudden weakness in your face, arm, or leg**	Yes<br>No<br>Unsure	I had this symptom before my infection started<br>This was one of the initial symptoms I had<br>The symptom started after my infection started<br>Unsure		Not at all<br>Mild amount<br>Moderate amount<br>Severe amount<br>Worst possible amount	It went away when my infection went away<br>It went away but the symptom lingered on for a while after my other symptoms went away. I do not have the symptom now<br>It went away for a while and then it returned. I do not have the symptom now<br>It went away for a while and then it returned. I have the symptom now<br>It did not go away and I still have the symptom now<br>I had it before my infection started. It has returned to normal<br>I had it before my infection started. It is worse than before I was infected
**Felt generally weak**	Yes<br>No<br>Unsure	I had this symptom before my infection started<br>This was one of the initial symptoms I had<br>The symptom started after my infection started<br>[illegible]		Not at all<br>Mild amount<br>Moderate amount<br>Severe amount<br>Worst possible amount	It went away when my infection went away<br>It went away but the symptom lingered on for a while after my other symptoms went away. I do not have the symptom now

		Unsure			It went away for a while and then it returned. I do not have the symptom now<br>It went away for a while and then it returned. I have the symptom now<br>It did not go away and I still have the symptom now<br>I had it before my infection started. It has returned to normal<br>I had it before my infection started. It is worse than before I was infected

Symptom	Did you have this symptom when you were recently infected?	When did this symptom begin?	How many days after your infection started did the symptom start?	When the symptom was at its worst, how much did it bother you?	How did this symptom end?
Felt delirious (not in your right mind)	Yes<br>No<br>Unsure	I had this symptom before my infection started<br>This was one of the initial symptoms I had<br>The symptom started after my infection started<br>Unsure		Not at all<br>Mild amount<br>Moderate amount<br>Severe amount<br>Worst possible amount	It went away when my infection went away<br>It went away but the symptom lingered on for a while after my other symptoms went away. I do not have the symptom now<br>It went away for a while and then it returned. I do not have the symptom now<br>It went away for a while and then it returned. I have the symptom now<br>It did not go away and I still have the symptom now<br>I had it before my infection started. It has returned to normal<br>I had it before my infection started. It is worse than before I was infected
Had hallucinations (saw or heard things that were not there)	Yes<br>No<br>Unsure	I had this symptom before my infection started<br>This was one of the initial symptoms I had<br>The symptom started after my infection started<br>Unsure		Not at all<br>Mild amount<br>Moderate amount<br>Severe amount<br>Worst possible amount	It went away when my infection went away<br>It went away but the symptom lingered on for a while after my other symptoms went away. I do not have the symptom now<br>It went away for a while and then it returned. I do not have the symptom now<br>It went away for a while and then it returned. I have the symptom now<br>It did not go away and I still have the symptom now<br>I had it before my infection started. It has returned to normal<br>I had it before my infection started. It is worse than before I was infected
Difficulty concentrating or remembering things	Yes<br>No<br>Unsure	I had this symptom before my infection started<br>This was one of the initial symptoms I had<br>The symptom started after my infection started<br>[illegible]		Not at all<br>Mild amount<br>Moderate amount<br>Severe amount<br>Worst possible amount	It went away when my infection went away<br>It went away but the symptom lingered on for a while after my other symptoms went away. I do not have the symptom now

		Unsure			It went away for a while and then it returned. I do not have the symptom now<br>It went away for a while and then it returned. I have the symptom now<br>It did not go away and I still have the symptom now<br>I had it before my infection started. It has returned to normal<br>I had it before my infection started. It is worse than before I was infected
**Symptom**	**Did you have this symptom when you were recently infected?**	**When did this symptom begin?**	**How many days after your infection started did the symptom start?**	**When the symptom was at its worst, how much did it bother you?**	**How did this symptom end?**
**Dizziness or lightheadedness**	Yes<br>No<br>Unsure	I had this symptom before my infection started<br>This was one of the initial symptoms I had<br>The symptom started after my infection started<br>Unsure		Not at all<br>Mild amount<br>Moderate amount<br>Severe amount<br>Worst possible amount	It went away when my infection went away<br>It went away but the symptom lingered on for a while after my other symptoms went away. I do not have the symptom now<br>It went away for a while and then it returned. I do not have the symptom now<br>It went away for a while and then it returned. I have the symptom now<br>It did not go away and I still have the symptom now<br>I had it before my infection started. It has returned to normal<br>I had it before my infection started. It is worse than before I was infected
**Difficulty with speaking or finding words**	Yes<br>No<br>Unsure	I had this symptom before my infection started<br>This was one of the initial symptoms I had<br>The symptom started after my infection started<br>Unsure		Not at all<br>Mild amount<br>Moderate amount<br>Severe amount<br>Worst possible amount	It went away when my infection went away<br>It went away but the symptom lingered on for a while after my other symptoms went away. I do not have the symptom now<br>It went away for a while and then it returned. I do not have the symptom now<br>It went away for a while and then it returned. I have the symptom now<br>It did not go away and I still have the symptom now<br>I had it before my infection started. It has returned to normal<br>I had it before my infection started. It is worse than before I was infected
**Difficulty with balance or coordination**	Yes<br>No<br>Unsure	I had this symptom before my infection started<br>This was one of the initial symptoms I had<br>The symptom started after my infection started<br>[illegible]		Not at all<br>Mild amount<br>Moderate amount<br>Severe amount<br>Worst possible amount	It went away when my infection went away<br>It went away but the symptom lingered on for a while after my other symptoms went away. I do not have the symptom now

		Unsure			It went away for a while and then it returned. I do not have the symptom now<br>It went away for a while and then it returned. I have the symptom now<br>It did not go away and I still have the symptom now<br>I had it before my infection started. It has returned to normal<br>I had it before my infection started. It is worse than before I was infected
**Symptom**	**Did you have this symptom when you were recently infected?**	**When did this symptom begin?**	**How many days after your infection started did the symptom start?**	**When the symptom was at its worst, how much did it bother you?**	**How did this symptom end?**
**Difficulty understanding words**	Yes<br>No<br>Unsure	I had this symptom before my infection started<br>This was one of the initial symptoms I had<br>The symptom started after my infection started<br>Unsure		Not at all<br>Mild amount<br>Moderate amount<br>Severe amount<br>Worst possible amount	It went away when my infection went away<br>It went away but the symptom lingered on for a while after my other symptoms went away. I do not have the symptom now<br>It went away for a while and then it returned. I do not have the symptom now<br>It went away for a while and then it returned. I have the symptom now<br>It did not go away and I still have the symptom now<br>I had it before my infection started. It has returned to normal<br>I had it before my infection started. It is worse than before I was infected
**Sensitive to food**	Yes<br>No<br>Unsure	I had this symptom before my infection started<br>This was one of the initial symptoms I had<br>The symptom started after my infection started<br>Unsure		Not at all<br>Mild amount<br>Moderate amount<br>Severe amount<br>Worst possible amount	It went away when my infection went away<br>It went away but the symptom lingered on for a while after my other symptoms went away. I do not have the symptom now<br>It went away for a while and then it returned. I do not have the symptom now<br>It went away for a while and then it returned. I have the symptom now<br>It did not go away and I still have the symptom now<br>I had it before my infection started. It has returned to normal<br>I had it before my infection started. It is worse than before I was infected
**Sensitive to chemicals**	Yes<br>No<br>Unsure	I had this symptom before my infection started<br>This was one of the initial symptoms I had<br>The symptom started after my infection started<br>[illegible]		Not at all<br>Mild amount<br>Moderate amount<br>Severe amount<br>Worst possible amount	It went away when my infection went away<br>It went away but the symptom lingered on for a while after my other symptoms went away. I do not have the symptom now

		Unsure			It went away for a while and then it returned. I do not have the symptom now<br>It went away for a while and then it returned. I have the symptom now<br>It did not go away and I still have the symptom now<br>I had it before my infection started. It has returned to normal<br>I had it before my infection started. It is worse than before I was infected
**Symptom**	**Did you have this symptom when you were recently infected?**	**When did this symptom begin?**	**How many days after your infection started did the symptom start?**	**When the symptom was at its worst, how much did it bother you?**	**How did this symptom end?**
**Sensitive to light**	Yes<br>No<br>Unsure	I had this symptom before my infection started<br>This was one of the initial symptoms I had<br>The symptom started after my infection started<br>Unsure		Not at all<br>Mild amount<br>Moderate amount<br>Severe amount<br>Worst possible amount	It went away when my infection went away<br>It went away but the symptom lingered on for a while after my other symptoms went away. I do not have the symptom now<br>It went away for a while and then it returned. I do not have the symptom now<br>It went away for a while and then it returned. I have the symptom now<br>It did not go away and I still have the symptom now<br>I had it before my infection started. It has returned to normal<br>I had it before my infection started. It is worse than before I was infected
**Sensitive to noise**	Yes<br>No<br>Unsure	I had this symptom before my infection started<br>This was one of the initial symptoms I had<br>The symptom started after my infection started<br>Unsure		Not at all<br>Mild amount<br>Moderate amount<br>Severe amount<br>Worst possible amount	It went away when my infection went away<br>It went away but the symptom lingered on for a while after my other symptoms went away. I do not have the symptom now<br>It went away for a while and then it returned. I do not have the symptom now<br>It went away for a while and then it returned. I have the symptom now<br>It did not go away and I still have the symptom now<br>I had it before my infection started. It has returned to normal<br>I had it before my infection started. It is worse than before I was infected
**Sensitive to touch**	Yes<br>No<br>Unsure	I had this symptom before my infection started<br>This was one of the initial symptoms I had<br>The symptom started after my infection started<br>[illegible]		Not at all<br>Mild amount<br>Moderate amount<br>Severe amount<br>Worst possible amount	It went away when my infection went away<br>It went away but the symptom lingered on for a while after my other symptoms went away. I do not have the symptom now

		Unsure			It went away for a while and then it returned. I do not have the symptom now<br>It went away for a while and then it returned. I have the symptom now<br>It did not go away and I still have the symptom now<br>I had it before my infection started. It has returned to normal<br>I had it before my infection started. It is worse than before I was infected

Symptom	Did you have this symptom when you were recently infected?	When did this symptom begin?	How many days after your infection started did the symptom start?	When the symptom was at its worst, how much did it bother you?	How did this symptom end?
Painful toes that changed color	Yes<br>No<br>Unsure	I had this symptom before my infection started<br>This was one of the initial symptoms I had<br>The symptom started after my infection started<br>Unsure		Not at all<br>Mild amount<br>Moderate amount<br>Severe amount<br>Worst possible amount	It went away when my infection went away<br>It went away but the symptom lingered on for a while after my other symptoms went away. I do not have the symptom now<br>It went away for a while and then it returned. I do not have the symptom now<br>It went away for a while and then it returned. I have the symptom now<br>It did not go away and I still have the symptom now<br>I had it before my infection started. It has returned to normal<br>I had it before my infection started. It is worse than before I was infected
Sores or ulcers in your mouth	Yes<br>No<br>Unsure	I had this symptom before my infection started<br>This was one of the initial symptoms I had<br>The symptom started after my infection started<br>Unsure		Not at all<br>Mild amount<br>Moderate amount<br>Severe amount<br>Worst possible amount	It went away when my infection went away<br>It went away but the symptom lingered on for a while after my other symptoms went away. I do not have the symptom now<br>It went away for a while and then it returned. I do not have the symptom now<br>It went away for a while and then it returned. I have the symptom now<br>It did not go away and I still have the symptom now<br>I had it before my infection started. It has returned to normal<br>I had it before my infection started. It is worse than before I was infected
Skin rash	Yes<br>No<br>Unsure	I had this symptom before my infection started<br>This was one of the initial symptoms I had<br>The symptom started after my infection started<br>[illegible]		Not at all<br>Mild amount<br>Moderate amount<br>Severe amount<br>Worst possible amount	It went away when my infection went away<br>It went away but the symptom lingered on for a while after my other symptoms went away. I do not have the symptom now

		Unsure			It went away for a while and then it returned. I do not have the symptom now<br>It went away for a while and then it returned. I have the symptom now<br>It did not go away and I still have the symptom now<br>I had it before my infection started. It has returned to normal<br>I had it before my infection started. It is worse than before I was infected

Symptom	Did you have this symptom when you were recently infected?	When did this symptom begin?	How many days after your infection started did the symptom start?	When the symptom was at its worst, how much did it bother you?	How did this symptom end?
Hair loss	Yes<br>No<br>Unsure	I had this symptom before my infection started<br>This was one of the initial symptoms I had<br>The symptom started after my infection started<br>Unsure		Not at all<br>Mild amount<br>Moderate amount<br>Severe amount<br>Worst possible amount	It went away when my infection went away<br>It went away but the symptom lingered on for a while after my other symptoms went away. I do not have the symptom now<br>It went away for a while and then it returned. I do not have the symptom now<br>It went away for a while and then it returned. I have the symptom now<br>It did not go away and I still have the symptom now<br>I had it before my infection started. It has returned to normal<br>I had it before my infection started. It is worse than before I was infected
Eye discharge	Yes<br>No<br>Unsure	I had this symptom before my infection started<br>This was one of the initial symptoms I had<br>The symptom started after my infection started<br>Unsure		Not at all<br>Mild amount<br>Moderate amount<br>Severe amount<br>Worst possible amount	It went away when my infection went away<br>It went away but the symptom lingered on for a while after my other symptoms went away. I do not have the symptom now<br>It went away for a while and then it returned. I do not have the symptom now<br>It went away for a while and then it returned. I have the symptom now<br>It did not go away and I still have the symptom now<br>I had it before my infection started. It has returned to normal<br>I had it before my infection started. It is worse than before I was infected
Eye redness	Yes<br>No<br>Unsure	I had this symptom before my infection started<br>This was one of the initial symptoms I had<br>The symptom started after my infection started<br>[illegible]		Not at all<br>Mild amount<br>Moderate amount<br>Severe amount<br>Worst possible amount	It went away when my infection went away<br>It went away but the symptom lingered on for a while after my other symptoms went away. I do not have the symptom now

		Unsure			It went away for a while and then it returned. I do not have the symptom now<br>It went away for a while and then it returned. I have the symptom now<br>It did not go away and I still have the symptom now<br>I had it before my infection started. It has returned to normal<br>I had it before my infection started. It is worse than before I was infected
**Symptom**	**Did you have this symptom when you were recently infected?**	**When did this symptom begin?**	**How many days after your infection started did the symptom start?**	**When the symptom was at its worst, how much did it bother you?**	**How did this symptom end?**
**Feeling of something in your eye**	Yes<br>No<br>Unsure	I had this symptom before my infection started<br>This was one of the initial symptoms I had<br>The symptom started after my infection started<br>Unsure		Not at all<br>Mild amount<br>Moderate amount<br>Severe amount<br>Worst possible amount	It went away when my infection went away<br>It went away but the symptom lingered on for a while after my other symptoms went away. I do not have the symptom now<br>It went away for a while and then it returned. I do not have the symptom now<br>It went away for a while and then it returned. I have the symptom now<br>It did not go away and I still have the symptom now<br>I had it before my infection started. It has returned to normal<br>I had it before my infection started. It is worse than before I was infected
**Itching of the eyes**	Yes<br>No<br>Unsure	I had this symptom before my infection started<br>This was one of the initial symptoms I had<br>The symptom started after my infection started<br>Unsure		Not at all<br>Mild amount<br>Moderate amount<br>Severe amount<br>Worst possible amount	It went away when my infection went away<br>It went away but the symptom lingered on for a while after my other symptoms went away. I do not have the symptom now<br>It went away for a while and then it returned. I do not have the symptom now<br>It went away for a while and then it returned. I have the symptom now<br>It did not go away and I still have the symptom now<br>I had it before my infection started. It has returned to normal<br>I had it before my infection started. It is worse than before I was infected
**Eye pain**	Yes<br>No<br>Unsure	I had this symptom before my infection started<br>This was one of the initial symptoms I had<br>The symptom started after my infection started<br>[illegible]		Not at all<br>Mild amount<br>Moderate amount<br>Severe amount<br>Worst possible amount	It went away when my infection went away<br>It went away but the symptom lingered on for a while after my other symptoms went away. I do not have the symptom now

		Unsure			It went away for a while and then it returned. I do not have the symptom now<br>It went away for a while and then it returned. I have the symptom now<br>It did not go away and I still have the symptom now<br>I had it before my infection started. It has returned to normal<br>I had it before my infection started. It is worse than before I was infected
**Symptom**	**Did you have this symptom when you were recently infected?**	**When did this symptom begin?**	**How many days after your infection started did the symptom start?**	**When the symptom was at its worst, how much did it bother you?**	**How did this symptom end?**
**Blurred vision**	Yes<br>No<br>Unsure	I had this symptom before my infection started<br>This was one of the initial symptoms I had<br>The symptom started after my infection started<br>Unsure		Not at all<br>Mild amount<br>Moderate amount<br>Severe amount<br>Worst possible amount	It went away when my infection went away<br>It went away but the symptom lingered on for a while after my other symptoms went away. I do not have the symptom now<br>It went away for a while and then it returned. I do not have the symptom now<br>It went away for a while and then it returned. I have the symptom now<br>It did not go away and I still have the symptom now<br>I had it before my infection started. It has returned to normal<br>I had it before my infection started. It is worse than before I was infected
**See specks or flashes of light**	Yes<br>No<br>Unsure	I had this symptom before my infection started<br>This was one of the initial symptoms I had<br>The symptom started after my infection started<br>Unsure		Not at all<br>Mild amount<br>Moderate amount<br>Severe amount<br>Worst possible amount	It went away when my infection went away<br>It went away but the symptom lingered on for a while after my other symptoms went away. I do not have the symptom now<br>It went away for a while and then it returned. I do not have the symptom now<br>It went away for a while and then it returned. I have the symptom now<br>It did not go away and I still have the symptom now<br>I had it before my infection started. It has returned to normal<br>I had it before my infection started. It is worse than before I was infected
**Dry mouth**	Yes<br>No<br>Unsure	I had this symptom before my infection started<br>This was one of the initial symptoms I had<br>The symptom started after my infection started<br>Unsure		Not at all<br>Mild amount<br>Moderate amount<br>Severe amount<br>Worst possible amount	It went away when my infection went away<br>It went away but the symptom lingered on for a while after my other symptoms went away. I do not have the symptom now<br>It went away for a while and then it returned. I do

					not have the symptom now<br>○ It went away for a while and then it returned. I have the symptom now<br>○ It did not go away and I still have the symptom now<br>○ I had it before my infection started. It has returned to normal<br>○ I had it before my infection started. It is worse than before I was infected

Overall, when your COVID-19 symptoms were at their worst, how bad or bothersome were they?

○ Not at all ○ Mild ○ Moderate ○ Severe ○ Worst possible amount

Overall, when your COVID-19 symptoms were at their worst, did they interfere with your daily activities?

○ Not at all ○ A little bit ○ Somewhat ○ Quite a bit ○ Complete interferes

Smell and Taste

The next section is focused on your experience of smell, taste, and food flavor during your recent COVID-19 infection.

*The following questions relate to your **sense of smell** (for example, sniffing flowers or soap, or smelling garbage) but **not the flavor of food** in your mouth.*

Rate your ability to smell DURING your recent COVID-19 infection by sliding the bar to the appropriate point:

No sense of smell {0 / 50} Excellent sense of smell

Have you experienced any of the following changes in smell with your recent COVID-19 infection (Select all that apply. If none apply, please leave blank.):

- ☐ I cannot smell at all
- ☐ Smells smell less strong than they did before
- ☐ Smells smell different than they did before
- ☐ I can smell things that are not there. An example would be smelling burning when nothing is on fire
- ☐ My sense of smell comes and goes

Rate how blocked your nose was DURING your recent COVID-19 infection by sliding the bar to the appropriate point:

Not at all blocked {0 / 50} Completely blocked

*The following question relates to your **sense of taste** (for example, sweetness, sourness, saltiness, bitterness) experienced in your mouth.*

Rate your ability to taste DURING your recent COVID-19 infection by sliding the bar to the appropriate point:

No sense of taste {0 / 50} Excellent sense of taste

Have you experienced changes to **specific tastes** with your recent COVID-19 infection (Select all that apply. If none apply, please leave blank.):

☐ Sweet ☐ Salty ☐ Sour ☐ Bitter ☐ Savory (Umami)

*The following question is related to other sensations in your mouth, like **burning, cooling, or tingling**. For example chili peppers, mint gum or candy, or carbonation.*

Rate your ability to feel these other sensations like burning, cooling, and tingling DURING your recent COVID-19 infection by sliding the bar to the appropriate point:

Not sensitive at all {0 / 50} Very sensitive

Nasal Swab, Blood, or Saliva Testing

This next set of questions will ask you about the COVID-19 testing you had during your recent infection.

Did you have a nasal swab, blood, or saliva test for the COVID-19 infection to see if you were infected? Yes / No / Unsure

Nasal Swab, Blood, or Saliva Testing Information

For each nasal swab, blood, or saliva test COVID-19 test you, please list the date, reason, and the result of each test. If you had multiple tests, then click on the "repeat" button to add additional tests.

Date of test	Type of test	Reason for test	Result of test
Format: YYYY-MM-D	Nasal swab<br>Blood<br>Saliva	Testing for work when I did not have symptoms<br>Testing for travel when I did not have symptoms<br>Testing when I did not have symptoms because I was in contact with a person infected with COVID-19<br>Testing because I had symptoms	Positive<br>Negative<br>Inconclusive

Repeat Cancel

Antibody Testing

Did you have antibody testing for the COVID-19 infection (blood test that looks to see if you have developed immunity)? Yes / No / Unsure

Antibody Testing Information

For each antibody test you have had, please list the date and the result of the test. If you had multiple tests, then click on the "repeat" button to add additional tests.

Date of test	Result of test
Format: YYYY-MM-DD HH:MM	Positive<br>Negative<br>Inconclusive

Repeat Cancel

Other Testing

Did you have any other type of COVID-19 testing? Yes / No / Unsure	Please describe any other type of COVID-19 testing you had:
During your recent infection, were you tested for the flu? Yes / No / Unsure	What was the result? Positive / Negative / Unsure

Care and Treatment of your COVID-19 Infection

This next section will ask you about the care and treatments that were used during your recent COVID-19 infection.

Care

What type of care did you have when you were recently ill with COVID-19? Please select all that apply.	☐ I did not seek medical care<br>☐ Telemedicine appointment<br>☐ In-office doctor appointment<br>☐ Urgent Care<br>☐ Emergency Room visit<br>☐ Admitted to Hospital<br>☐ Other (specify)	Describe other medical care:

Care Information

Please provide us with information about any visits to urgent care centers, emergency rooms, and/or hospitals you had because of your recent COVID-19 infection. If you had multiple visits, then click "repeat" to add information on additional visits.

Date of Visit	Format: YYYY-MM-DD HH:MM

Type of Visit	○ Urgent Care<br>○ Emergency Room<br>○ Admitted to Hospital	Did you go to the Intensive Care Unit (ICU)?	○ Yes ○ No ○ Unsure	Did you need a ventilator?	○ Yes ○ No ○ Unsure	Did you need Extracorporeal membrane oxygenation (ECMO)?	○ Yes ○ No ○ Unsure

Date of Discharge	Format: YYYY-MM-DD HH:MM

Repeat Cancel

Treatments

Did you receive any of the following treatments during your recent COVID-19 infection? Please mark all that apply.

Oxygen through a nasal cannula:	○ Yes ○ No ○ Unsure
Oxygen through a face mask:	○ Yes ○ No ○ Unsure
Hydroxychloroquine:	○ Yes ○ No ○ Unsure
Azithromycin:	○ Yes ○ No ○ Unsure
Oral steroids:	○ Yes ○ No ○ Unsure
Inhaled steroids:	○ Yes ○ No ○ Unsure
Intravenous steroids:	○ Yes ○ No ○ Unsure

Inhalers and Nebulizers: ○ Yes ○ No ○ Unsure	Describe what type of inhalers and nebulizers were used:
Remdesivir:	○ Yes ○ No ○ Unsure
Acetaminophen/Tylenol:	○ Yes ○ No ○ Unsure
Non-steroidal anti-inflammatory medications (naproxen, ibuprofen):	○ Yes ○ No ○ Unsure
Cough medication:	○ Yes ○ No ○ Unsure
Cold and flu Medication:	○ Yes ○ No ○ Unsure
Antibiotics: ○ Yes ○ No ○ Unsure	Describe what type of antibiotics were used:
Lopinavir:	○ Yes ○ No ○ Unsure
Ritonavir:	○ Yes ○ No ○ Unsure
Ribavirin:	○ Yes ○ No ○ Unsure
Oseltamivir (tamiflu):	○ Yes ○ No ○ Unsure
Convalescent plasma:	○ Yes ○ No ○ Unsure
Vitamin C:	○ Yes ○ No ○ Unsure
Zinc:	○ Yes ○ No ○ Unsure
Vitamin D:	○ Yes ○ No ○ Unsure
Anticoagulants:	○ Yes ○ No ○ Unsure
Any other treatments? ○ Yes ○ No ○ Unsure	Describe any other treatments used:
Were you part of a research study or clinical trial? ○ Yes ○ No ○ Unsure	Please describe where the study was held, and what it tested:

OPTIONAL DESCRIPTION

Please take some time to describe in the text box how you recall your general health during your recent COVID-19 infection. This is an opportunity to help us better understand how you felt when you were acutely ill with COVID-19 in your own words. Please tell us about your overall well-being and give a few examples of any unique problems you experienced that we have not asked about in the above questions.	

View eForm

Test eForm rules

Show hidden eForm elements

Short Form Health Survey (SF36v2)

Main

Your Health and Well-Being

This survey asks for your views about your health. This information will help keep track of how you feel and how well you are able to do your usual activities. *Thank you for completing this su*

For each of the following questions, please mark an X in the one box that best describes your answer.

1. In general, would you say your health is:

- Excellent
- Very good
- Good
- Fair
- Poor

2. Compared to one year ago, how would you rate your health in general now?

- Much better now than one year ago
- Somewhat better now than one year ago
- About the same as one year ago
- Somewhat worse now than one year ago
- Much worse now than one year ago

3. The following questions are about activities you might do during a typical day. Does your health now limit you in these activities? If so, how much?

a. Vigorous activities, such as running, lifting heavy objects, participating in strenuous sports...................	Yes, limited a lot	Yes, limited a little	No, not limited at all
b. Moderate activities, such as moving a table, pushing a vacuum cleaner, bowling, or playing golf...................	Yes, limited a lot	Yes, limited a little	No, not limited at all
c. Lifting or carrying groceries...................	Yes, limited a lot	Yes, limited a little	No, not limited at all
d. Climbing several flights of stairs...................	Yes, limited a lot	Yes, limited a little	No, not limited at all
e. Climbing one flight of stairs...................	Yes, limited a lot	Yes, limited a little	No, not limited at all
f. Bending, kneeling, or stooping...................	Yes, limited a lot	Yes, limited a little	No, not limited at all
g. Walking more than a mile...................	Yes, limited a lot	Yes, limited a little	No, not limited at all
h. Walking several hundred yards...................	Yes, limited a lot	Yes, limited a little	No, not limited at all
i. Walking one hundred yards...................	Yes, limited a lot	Yes, limited a little	No, not limited at all
j. Bathing or dressing yourself...................	Yes, limited a lot	Yes, limited a little	No, not limited at all

4. During the past 4 weeks, how much of the time have you had any of the following problems with your work or other regular daily activities as a result of your physical he

View eForm

a. Cut down on the amount time time you spent on work or other activities..................	All of the time	Most of the time	Some of the time	A little of the time	None of time
b. Accomplished less than you would like	All of the time	Most of the time	Some of the time	A little of the time	None of time
c. Were limited in the kind of work or other activities	All of the time	Most of the time	Some of the time	A little of the time	None of time
d. Had difficulty performing the work or other activities (for example, it took extra effort)	All of the time	Most of the time	Some of the time	A little of the time	None of time

5. During the past 4 weeks, how much of the time have you had any of the following problems with your work or other regular daily activities as a result of any emotional problems (such as feeling depressed or anxious)?

a. Cut down on the amount of time you spent on work or other activities	All of the time	Most of the time	Some of the time	A little of the time	None of time
b. Accomplished less than you would like	All of the time	Most of the time	Some of the time	A little of the time	None of time
c. Did work or other activities less carefully than usual	All of the time	Most of the time	Some of the time	A little of the time	None of time

**6. During the past 4 weeks, to what extent has your physical health or emotional problems interfered with your normal social activities with family, friends, neighbors, or groups?**	Not at all	Slightly	Moderately	Quite a bit	Extreme

**7. How much bodily pain have you had during the past 4 weeks?**	None	Very mild	Mild	Moderate	Severe	Very severe

**8. During the past 4 weeks, how much did pain interfere with your normal work (including both work outside the home and housework)?**	Not at all	A little bit	Moderately	Quite a bit	Extreme

9. These questions are about how you feel and how things have been with you during the past 4 weeks. For each question, please give the one answer that comes closest t way you have been feeling. How much of the time during the past 4 weeks…

a. Did you feel full of life?	All of the time	Most of the time	Some of the time	A little of the time	None of time
b. Have you been very nervous?	All of the time	Most of the time	Some of the time	A little of the time	None of time
c. Have you felt so down in the dumps that nothing could cheer you up?	All of the time	Most of the time	Some of the time	A little of the time	None of time
d. Have you felt calm and peaceful?	All of the time	Most of the time	Some of the time	A little of the time	None of time
e. Did you have a lot of energy?	All of the time	Most of the time	Some of the time	A little of the time	None of time
f. Have you felt downhearted and depressed?	All of the time	Most of the time	Some of the time	A little of the time	None of time
g. Did you feel worn out?	All of the time	Most of the time	Some of the time	A little of the time	None of time
h. Have you been happy?	All of the time	Most of the time	Some of the time	A little of the time	None of time
i. Did you feel tired?	All of the time	Most of the time	Some of the time	A little of the time	None of time

10. During the <u>past 4 weeks</u>, how much of the time has your <u>physical health or emotional problems</u> interfered with your social activities (like visiting with friends, relatives,

View eForm

- ○ All of the time
- ○ Most of the time
- ○ Some of the time
- ○ A little of the time
- ○ None of the time

11. How TRUE or FALSE is <u>each</u> of the following statements for you?

a. I seem to get sick a little easier than other people	○ Definitely true	○ Mostly true	○ Don't know	○ Mostly false	○ Definitel
b. I am as healthy as anybody I know	○ Definitely true	○ Mostly true	○ Don't know	○ Mostly false	○ Definitel
c. I expect my health to get worse	○ Definitely true	○ Mostly true	○ Don't know	○ Mostly false	○ Definitel
d. My health is excellent	○ Definitely true	○ Mostly true	○ Don't know	○ Mostly false	○ Definitel

Thank you for completing these questions!

After COVID-19 Infection

AFTER COVID-19 INFECTION

The next set of questions are to help us better understand how you felt after your recent COVID-19 infection, also known as "recovery". We know it is not always possible to be sure exactly when a person "recovers" from an acute infection. We also know that you may have recovered in some ways but not others.

If you know the day when you started to feel better from your infection, please use that date as the start date of your recovery. If you do not know when you started to recover, please use the date when your worst symptoms first improved.

If you do not understand a question, please mark "Unsure".

What date do you think you first started to recover from your recent COVID-19 infection?<br><br>*Click on the calendar and click on the date. Use the slide to indicate the time. If you don't know the time, then leave it blank.*	Format: YYYY-MM-DD HH:MM
Since you started to recover from your recent COVID-19 infection, how much have you returned to your usual state of health compared to before the infection?	○ I continue to be as sick with my COVID-19 symptoms as when I was at my sickest<br>○ I feel better than I did when I was at my sickest, but I have not completely recovered to my usual health. I still have symptoms that remain<br>○ Many of my symptoms that I had during my recent infection have gotten better, but I have developed other symptoms and problems and am not back to my usual health<br>○ I am back to my usual state of health
Compared to when your COVID-19 symptoms were at their worst, how bad or bothersome are your current symptoms?	○ No symptoms<br>○ Mild<br>○ Moderate<br>○ Severe<br>○ Worst possible amount
Compared to when your COVID-19 symptoms were at their worst, how much are your current symptoms interfering with your daily activities?	○ Not at all<br>○ A little bit<br>○ Somewhat<br>○ Quite a bit<br>○ Completely interferes
Would you say you have fully recovered from your recent COVID-19 infection?	○ Yes<br>○ No<br>○ Unsure
What date do you think you fully recovered from your recent COVID-19 infection?<br><br>*Click on the calendar and click on the date. Use the slide to indicate the time. If you don't know the time, then leave it blank.*	Format: YYYY-MM-DD HH:MM

GENERAL ISSUES

After your recent COVID-19 infection, are you experiencing any of the following symptoms or problems?

Do you still "feel like I am sick" with COVID-19?	○ Yes<br>○ No<br>○ Unsure
Tell us whether you agree or disagree with the following statements. If you agree with the statement and believe it is true, mark Yes. If you disagree with the statement and believe it is not true, mark No. If you are unsure, mark Unsure.	
"Feeling like I am sick" is getting better over time:	○ Yes<br>○ No<br>○ Unsure

"Feeling like I am sick" is getting worse over time:	Yes<br>No<br>Unsure
"Feeling like I am sick" is concerning to me:	Yes<br>No<br>Unsure

Symptom	Are you currently having this symptom?	Did this symptom first begin after you felt like you were already recovering/after the acute phase?	How much is the symptom currently bothering you?
Fever of >100.4	Yes<br>No<br>Unsure	Yes<br>No<br>Unsure	Not at all<br>Mild amount<br>Moderate amount<br>Severe amount<br>Worst possible amount
Subjective Fever (felt feverish but did not measure a temperature)	Yes<br>No<br>Unsure	Yes<br>No<br>Unsure	Not at all<br>Mild amount<br>Moderate amount<br>Severe amount<br>Worst possible amount
Chills	Yes<br>No<br>Unsure	Yes<br>No<br>Unsure	Not at all<br>Mild amount<br>Moderate amount<br>Severe amount<br>Worst possible amount
Repeated shaking with chills	Yes<br>No<br>Unsure	Yes<br>No<br>Unsure	Not at all<br>Mild amount<br>Moderate amount<br>Severe amount<br>Worst possible amount
Runny nose	Yes<br>No<br>Unsure	Yes<br>No<br>Unsure	Not at all<br>Mild amount<br>Moderate amount<br>Severe amount<br>Worst possible amount
Sore throat	Yes<br>No<br>Unsure	Yes<br>No<br>Unsure	Not at all<br>Mild amount<br>Moderate amount<br>Severe amount<br>Worst possible amount
"Flu-like" symptoms	Yes<br>No<br>Unsure	Yes<br>No<br>Unsure	Not at all<br>Mild amount<br>Moderate amount<br>Severe amount<br>Worst possible amount
Cold hands and feet	Yes<br>No<br>Unsure	Yes<br>No<br>Unsure	Not at all<br>Mild amount<br>Moderate amount<br>Severe amount<br>Worst possible amount

Swollen or tender glands (lymph nodes)	Yes<br>No<br>Unsure	Yes<br>No<br>Unsure	Not at all<br>Mild amount<br>Moderate amount<br>Severe amount<br>Worst possible amount

FATIGUE AND MALAISE ISSUES

Are you having problems with fatigue, general lack of energy, or malaise?	Yes<br>No<br>Unsure

Please tell us whether you agree or disagree with the following statements. If you agree with the statement and believe it is true, mark Yes. If you disagree with the statement and believe it is not true, mark No. If you are unsure, mark Unsure.

"Fatigue" is getting better over time:	Yes<br>No<br>Unsure
"Fatigue" is getting worse over time:	Yes<br>No<br>Unsure
"Fatigue" is concerning to me:	Yes<br>No<br>Unsure
Do you now need a new medication or more medication for fatigue since your COVID-19 infection?	Yes<br>No

Symptom	Are you currently having this symptom?	Did this symptom first begin <u>after</u> you felt like you were already recovering/after the acute phase?	How much is the symptom <u>currently</u> bothering you?
Fatigue	Yes<br>No<br>Unsure	Yes<br>No<br>Unsure	Not at all<br>Mild amount<br>Moderate amount<br>Severe amount<br>Worst possible amount
Fatigue is made worse by physical or mental activity or work	Yes<br>No<br>Unsure	Yes<br>No<br>Unsure	Not at all<br>Mild amount<br>Moderate amount<br>Severe amount<br>Worst possible amount
Fatigue greatly reduces my activity	Yes<br>No<br>Unsure	Yes<br>No<br>Unsure	Not at all<br>Mild amount<br>Moderate amount<br>Severe amount<br>Worst possible amount
Dead, heavy feeling after exercise	Yes<br>No<br>Unsure	Yes<br>No<br>Unsure	Not at all<br>Mild amount<br>Moderate amount<br>Severe amount<br>Worst possible amount
Fatigued the day after mild, everyday activities	Yes<br>No<br>Unsure	Yes<br>No<br>Unsure	Not at all<br>Mild amount<br>Moderate amount

			○ Severe amount<br>○ Worst possible amount
Mentally tired after the slightest effort	○ Yes<br>○ No<br>○ Unsure	○ Yes<br>○ No<br>○ Unsure	○ Not at all<br>○ Mild amount<br>○ Moderate amount<br>○ Severe amount<br>○ Worst possible amount
Minimum exercise makes me physically tired	○ Yes<br>○ No<br>○ Unsure	○ Yes<br>○ No<br>○ Unsure	○ Not at all<br>○ Mild amount<br>○ Moderate amount<br>○ Severe amount<br>○ Worst possible amount
Physically drained or sick after mild activity	○ Yes<br>○ No<br>○ Unsure	○ Yes<br>○ No<br>○ Unsure	○ Not at all<br>○ Mild amount<br>○ Moderate amount<br>○ Severe amount<br>○ Worst possible amount
Takes more than a day to recover from tiring activities	○ Yes<br>○ No<br>○ Unsure	○ Yes<br>○ No<br>○ Unsure	○ Not at all<br>○ Mild amount<br>○ Moderate amount<br>○ Severe amount<br>○ Worst possible amount
Limit my activity to avoid having my fatigue get worse	○ Yes<br>○ No<br>○ Unsure	○ Yes<br>○ No<br>○ Unsure	○ Not at all<br>○ Mild amount<br>○ Moderate amount<br>○ Severe amount<br>○ Worst possible amount

PAIN ISSUES

Are you having problems with aches, pains, or soreness?	○ Yes<br>○ No<br>○ Unsure
Please tell us whether you agree or disagree with the following statements. If you agree with the statement and believe it is true, mark Yes. If you disagree with the statement and believe it is not true, mark No. If you are unsure, mark Unsure.	
Pain, aches, or soreness is getting better over time:	○ Yes<br>○ No<br>○ Unsure
Pain, aches, or soreness getting worse over time:	○ Yes<br>○ No<br>○ Unsure
Pain, aches, or soreness is concerning to me:	○ Yes<br>○ No<br>○ Unsure
Do you now need a new medication or more medication for pain, aches, or soreness since your COVID-19 infection?	○ Yes<br>○ No

Symptom	Are you currently having this symptom?	Did this symptom first begin [illegible]	How much is the symptom <u>currently</u> bothering you?

		after<br>you felt like you were already recovering/after the acute phase?	
Muscle aches or pains	Yes<br>No<br>Unsure	Yes<br>No<br>Unsure	Not at all<br>Mild amount<br>Moderate amount<br>Severe amount<br>Worst possible amount
Joint aches or pains	Yes<br>No<br>Unsure	Yes<br>No<br>Unsure	Not at all<br>Mild amount<br>Moderate amount<br>Severe amount<br>Worst possible amount
Headaches	Yes<br>No<br>Unsure	Yes<br>No<br>Unsure	Not at all<br>Mild amount<br>Moderate amount<br>Severe amount<br>Worst possible amount
"Hurt all over"	Yes<br>No<br>Unsure	Yes<br>No<br>Unsure	Not at all<br>Mild amount<br>Moderate amount<br>Severe amount<br>Worst possible amount
Jaw pain/discomfort	Yes<br>No<br>Unsure	Yes<br>No<br>Unsure	Not at all<br>Mild amount<br>Moderate amount<br>Severe amount<br>Worst possible amount
Pelvic Pain	Yes<br>No<br>Unsure	Yes<br>No<br>Unsure	Not at all<br>Mild amount<br>Moderate amount<br>Severe amount<br>Worst possible amount
Genital Pain	Yes<br>No<br>Unsure	Yes<br>No<br>Unsure	Not at all<br>Mild amount<br>Moderate amount<br>Severe amount<br>Worst possible amount
Chest Pain	Yes<br>No<br>Unsure	Yes<br>No<br>Unsure	Not at all<br>Mild amount<br>Moderate amount<br>Severe amount<br>Worst possible amount
Pain to Light Touch	Yes<br>No<br>Unsure	Yes<br>No<br>Unsure	Not at all<br>Mild amount<br>Moderate amount<br>Severe amount<br>Worst possible amount
Sore the day after non-strenuous everyday activities	Yes<br>No<br>Unsure	Yes<br>No<br>Unsure	Not at all<br>Mild amount<br>Moderate amount

			◯ Severe amount<br>◯ Worst possible amount

BREATHING AND COUGHING ISSUES

Are you having problems with breathing?	◯ Yes<br>◯ No<br>◯ Unsure
Please tell us whether you agree or disagree with the following statements. If you agree with the statement and believe it is true, mark Yes. If you disagree with the statement and believe it is not true, mark No. If you are unsure, mark Unsure.	
Breathing is getting better over time:	◯ Yes<br>◯ No<br>◯ Unsure
Breathing is getting worse over time:	◯ Yes<br>◯ No<br>◯ Unsure
Breathing is concerning to me:	◯ Yes<br>◯ No<br>◯ Unsure
Do you now need a new medication or more medication for breathing since your COVID-19 infection?	◯ Yes<br>◯ No
Are you having problems with coughing?	◯ Yes<br>◯ No<br>◯ Unsure
Please tell us whether you agree or disagree with the following statements. If you agree with the statement and believe it is true, mark Yes. If you disagree with the statement and believe it is not true, mark No. If you are unsure, mark Unsure.	
Coughing is getting better over time:	◯ Yes<br>◯ No<br>◯ Unsure
Coughing is getting worse over time:	◯ Yes<br>◯ No<br>◯ Unsure
Coughing is concerning to me:	◯ Yes<br>◯ No<br>◯ Unsure
Do you now need a new medication or more medication for coughing since your COVID-19 infection?	◯ Yes<br>◯ No

Symptom	Are you currently having this symptom?	Did this symptom first begin <u>after</u> you felt like you were already recovering/after the acute phase?	How much is the symptom <u>currently</u> bothering you?
Dry Cough	◯ Yes<br>◯ No<br>◯ Unsure	◯ Yes<br>◯ No<br>◯ Unsure	◯ Not at all<br>◯ Mild amount<br>◯ Moderate amount<br>◯ Severe amount<br>◯ Worst possible amount
Wet Cough	◯ Yes<br>◯ No<br>◯ Unsure	◯ Yes<br>◯ No<br>◯ Unsure	◯ Not at all<br>◯ Mild amount<br>◯ Moderate amount<br>◯ Severe amount<br>◯ Worst possible amount

Shortness of breath at rest	Yes<br>No<br>Unsure	Yes<br>No<br>Unsure	Not at all<br>Mild amount<br>Moderate amount<br>Severe amount<br>Worst possible amount
Shortness of breath when moving	Yes<br>No<br>Unsure	Yes<br>No<br>Unsure	Not at all<br>Mild amount<br>Moderate amount<br>Severe amount<br>Worst possible amount
Wheezing (makes noise when breathing out)	Yes<br>No<br>Unsure	Yes<br>No<br>Unsure	Not at all<br>Mild amount<br>Moderate amount<br>Severe amount<br>Worst possible amount
Chest pain when taking a deep breath	Yes<br>No<br>Unsure	Yes<br>No<br>Unsure	Not at all<br>Mild amount<br>Moderate amount<br>Severe amount<br>Worst possible amount
Effort to keep breathing	Yes<br>No<br>Unsure	Yes<br>No<br>Unsure	Not at all<br>Mild amount<br>Moderate amount<br>Severe amount<br>Worst possible amount
Breathing catches or stutters	Yes<br>No<br>Unsure	Yes<br>No<br>Unsure	Not at all<br>Mild amount<br>Moderate amount<br>Severe amount<br>Worst possible amount
Breathing being erratic	Yes<br>No<br>Unsure	Yes<br>No<br>Unsure	Not at all<br>Mild amount<br>Moderate amount<br>Severe amount<br>Worst possible amount

DIGESTIVE AND STOMACH ISSUES

Are you having digestive or stomach problems?	Yes<br>No<br>Unsure
Please tell us whether you agree or disagree with the following statements. If you agree with the statement and believe it is true, mark Yes. If you disagree with the statement and believe it is not true, mark No. If you are unsure, mark Unsure.	
Digestive or stomach problems are getting better over time:	Yes<br>No<br>Unsure
Digestive or stomach problems are getting worse over time:	Yes<br>No<br>Unsure

Digestive or stomach problems are concerning to me:	◯ Yes<br>◯ No<br>◯ Unsure
Do you now need a new medication or more medication to help digestive or stomach problems since your COVID-19 infection?	◯ Yes<br>◯ No

Symptom	Are you currently having this symptom?	Did this symptom first begin <u>after</u> you felt like you were already recovering/after the acute phase?	How much is the symptom <u>currently</u> bothering you?
Dry mouth	◯ Yes<br>◯ No<br>◯ Unsure	◯ Yes<br>◯ No<br>◯ Unsure	◯ Not at all<br>◯ Mild amount<br>◯ Moderate amount<br>◯ Severe amount<br>◯ Worst possible amount
Nausea	◯ Yes<br>◯ No<br>◯ Unsure	◯ Yes<br>◯ No<br>◯ Unsure	◯ Not at all<br>◯ Mild amount<br>◯ Moderate amount<br>◯ Severe amount<br>◯ Worst possible amount
Vomiting	◯ Yes<br>◯ No<br>◯ Unsure	◯ Yes<br>◯ No<br>◯ Unsure	◯ Not at all<br>◯ Mild amount<br>◯ Moderate amount<br>◯ Severe amount<br>◯ Worst possible amount
Loss of appetite	◯ Yes<br>◯ No<br>◯ Unsure	◯ Yes<br>◯ No<br>◯ Unsure	◯ Not at all<br>◯ Mild amount<br>◯ Moderate amount<br>◯ Severe amount<br>◯ Worst possible amount
Bloating or Gas	◯ Yes<br>◯ No<br>◯ Unsure	◯ Yes<br>◯ No<br>◯ Unsure	◯ Not at all<br>◯ Mild amount<br>◯ Moderate amount<br>◯ Severe amount<br>◯ Worst possible amount
Heartburn	◯ Yes<br>◯ No<br>◯ Unsure	◯ Yes<br>◯ No<br>◯ Unsure	◯ Not at all<br>◯ Mild amount<br>◯ Moderate amount<br>◯ Severe amount<br>◯ Worst possible amount
Stomach or abdominal pain	◯ Yes<br>◯ No<br>◯ Unsure	◯ Yes<br>◯ No<br>◯ Unsure	◯ Not at all<br>◯ Mild amount<br>◯ Moderate amount<br>◯ Severe amount<br>◯ Worst possible amount
Diarrhea (3 or more looser than normal stools in a 24 hour period)	◯ Yes<br>◯ No<br>◯ Unsure	◯ Yes<br>◯ No<br>◯ Unsure	◯ Not at all<br>◯ Mild amount<br>◯ Moderate amount<br>◯ Severe amount<br>◯ Worst possible amount

Constipation	Yes<br>No<br>Unsure	Yes<br>No<br>Unsure	Not at all<br>Mild amount<br>Moderate amount<br>Severe amount<br>Worst possible amount

SLEEPING ISSUES

Are you having problems with sleeping?	Yes<br>No<br>Unsure

Please tell us whether you agree or disagree with the following statements. If you agree with the statement and believe it is true, mark Yes. If you disagree with the statement and believe it is not true, mark No. If you are unsure, mark Unsure.

Problems with sleep are getting better over time:	Yes<br>No<br>Unsure
Problems with sleep are getting worse over time:	Yes<br>No<br>Unsure
Problems with sleep are concerning to me:	Yes<br>No<br>Unsure
Do you now need a new medication, more medication, or devices for sleep since your COVID-19 infection?	Yes<br>No

Symptom	Are you currently having this symptom?	Did this symptom first begin <u>after</u> you felt like you were already recovering/after the acute phase?	How much is the symptom <u>currently</u> bothering you?
Trouble sleeping	Yes<br>No<br>Unsure	Yes<br>No<br>Unsure	Not at all<br>Mild amount<br>Moderate amount<br>Severe amount<br>Worst possible amount
Unrefreshed sleep or not feeling well rested after sleeping	Yes<br>No<br>Unsure	Yes<br>No<br>Unsure	Not at all<br>Mild amount<br>Moderate amount<br>Severe amount<br>Worst possible amount
Need to nap daily	Yes<br>No<br>Unsure	Yes<br>No<br>Unsure	Not at all<br>Mild amount<br>Moderate amount<br>Severe amount<br>Worst possible amount
Problems falling asleep	Yes<br>No<br>Unsure	Yes<br>No<br>Unsure	Not at all<br>Mild amount<br>Moderate amount<br>Severe amount<br>Worst possible amount
Problems staying asleep	Yes<br>No<br>Unsure	Yes<br>No<br>Unsure	Not at all<br>Mild amount<br>Moderate amount

			Severe amount<br>Worst possible amount
Waking up early in the morning	Yes<br>No<br>Unsure	Yes<br>No<br>Unsure	Not at all<br>Mild amount<br>Moderate amount<br>Severe amount<br>Worst possible amount
Sleep all day and stay awake all night	Yes<br>No<br>Unsure	Yes<br>No<br>Unsure	Not at all<br>Mild amount<br>Moderate amount<br>Severe amount<br>Worst possible amount
Feeling like it was difficult to stay awake	Yes<br>No<br>Unsure	Yes<br>No<br>Unsure	Not at all<br>Mild amount<br>Moderate amount<br>Severe amount<br>Worst possible amount

COGNITIVE ISSUES

Are you having problems with thinking, concentrating, remembering, or communicating?	Yes<br>No<br>Unsure

Please tell us whether you agree or disagree with the following statements. If you agree with the statement and believe it is true, mark Yes. If you disagree with the statement and believe it is not true, mark No. If you are unsure, mark Unsure.

Problems with thinking, concentrating, remembering, or communicating are getting better over time:	Yes<br>No<br>Unsure
Problems with thinking, concentrating, remembering, or communicating are getting worse over time:	Yes<br>No<br>Unsure
Problems with thinking, concentrating, remembering, or communicating are concerning to me:	Yes<br>No<br>Unsure
Do you now need a new medication or more medication for thinking, concentrating, remembering, or communicating since your COVID-19 infection?	Yes<br>No

Symptom	Are you currently having this symptom?	Did this symptom first begin <u>after</u> you felt like you were already recovering/after the acute phase?	How much is the symptom <u>currently</u> bothering you?
Difficulty concentrating or paying attention	Yes<br>No<br>Unsure	Yes<br>No<br>Unsure	Not at all<br>Mild amount<br>Moderate amount<br>Severe amount<br>Worst possible amount
Difficulty with remembering	Yes<br>No<br>Unsure	Yes<br>No<br>Unsure	Not at all<br>Mild amount<br>Moderate amount<br>Severe amount<br>Worst possible amount

Difficulty with speaking or finding words	Yes<br>No<br>Unsure	Yes<br>No<br>Unsure	Not at all<br>Mild amount<br>Moderate amount<br>Severe amount<br>Worst possible amount
Feel delirious (not in your right mind)	Yes<br>No<br>Unsure	Yes<br>No<br>Unsure	Not at all<br>Mild amount<br>Moderate amount<br>Severe amount<br>Worst possible amount
Have hallucinations (seeing or hearing things that are not there)	Yes<br>No<br>Unsure	Yes<br>No<br>Unsure	Not at all<br>Mild amount<br>Moderate amount<br>Severe amount<br>Worst possible amount
Trouble finding my way around	Yes<br>No<br>Unsure	Yes<br>No<br>Unsure	Not at all<br>Mild amount<br>Moderate amount<br>Severe amount<br>Worst possible amount
Trouble with doing simple math	Yes<br>No<br>Unsure	Yes<br>No<br>Unsure	Not at all<br>Mild amount<br>Moderate amount<br>Severe amount<br>Worst possible amount
Being confused	Yes<br>No<br>Unsure	Yes<br>No<br>Unsure	Not at all<br>Mild amount<br>Moderate amount<br>Severe amount<br>Worst possible amount

BALANCE ISSUES

Are you having problems with feeling dizzy, lightheaded, or feeling as if you are going to collapse when standing?	Yes<br>No<br>Unsure

Please tell us whether you agree or disagree with the following statements. If you agree with the statement and believe it is true, mark Yes. If you disagree with the statement and believe it is not true, mark No. If you are unsure, mark Unsure.

Problems with dizzy, lightheaded, or feeling as if you are going to collapse when standing are getting better over time:	Yes<br>No<br>Unsure
Problems with dizzy, lightheaded, or feeling as if you are going to collapse when standing are getting worse over time:	Yes<br>No<br>Unsure
Problems with dizzy, lightheaded, or feeling as if you are going to collapse when standing are concerning to me:	Yes<br>No<br>Unsure
Do you now need a new medication or more medication for dizzy, lightheaded, or feeling as if you are going to collapse when standing since your COVID-19 infection?	Yes<br>No

Symptom	Are you currently having this symptom?	Did this symptom first begin	How much is the symptom currently bothering you?

		after you felt like you were already recovering/after the acute phase?	
Dizziness	Yes<br>No<br>Unsure	Yes<br>No<br>Unsure	Not at all<br>Mild amount<br>Moderate amount<br>Severe amount<br>Worst possible amount
Lightheadedness	Yes<br>No<br>Unsure	Yes<br>No<br>Unsure	Not at all<br>Mild amount<br>Moderate amount<br>Severe amount<br>Worst possible amount
Feel dizzy or lightheaded when moving to sit up	Yes<br>No<br>Unsure	Yes<br>No<br>Unsure	Not at all<br>Mild amount<br>Moderate amount<br>Severe amount<br>Worst possible amount
Feel dizzy or lightheaded when moving to stand up	Yes<br>No<br>Unsure	Yes<br>No<br>Unsure	Not at all<br>Mild amount<br>Moderate amount<br>Severe amount<br>Worst possible amount
Feel weak when standing	Yes<br>No<br>Unsure	Yes<br>No<br>Unsure	Not at all<br>Mild amount<br>Moderate amount<br>Severe amount<br>Worst possible amount
Change in sweating	Yes<br>No<br>Unsure	Yes<br>No<br>Unsure	Not at all<br>Mild amount<br>Moderate amount<br>Severe amount<br>Worst possible amount
Fainting	Yes<br>No<br>Unsure	Yes<br>No<br>Unsure	Not at all<br>Mild amount<br>Moderate amount<br>Severe amount<br>Worst possible amount

ENVIRONMENTAL SENSITIVITY ISSUES

Are you having problems with body sensitivity to the things around you in the environment?	Yes<br>No<br>Unsure
Please tell us whether you agree or disagree with the following statements. If you agree with the statement and believe it is true, mark Yes. If you disagree with the statement and believe it is not true, mark No. If you are unsure, mark Unsure.	
Problems with body sensitivity are getting better over time:	Yes<br>No<br>Unsure
Problems with body sensitivity are getting worse over time:	Yes<br>No<br>Unsure

Problems with body sensitivity are concerning to me:	◯ Yes<br>◯ No<br>◯ Unsure
Do you now need a new medication or more medication for body sensitivity since your COVID-19 infection?	◯ Yes<br>◯ No

Symptom	Are you currently having this symptom?	Did this symptom first begin <u>after</u> you felt like you were already recovering/after the acute phase?	How much is the symptom <u>currently</u> bothering you?
Sensitive to Food	◯ Yes<br>◯ No<br>◯ Unsure	◯ Yes<br>◯ No<br>◯ Unsure	◯ Not at all<br>◯ Mild amount<br>◯ Moderate amount<br>◯ Severe amount<br>◯ Worst possible amount
Sensitive to Chemicals	◯ Yes<br>◯ No<br>◯ Unsure	◯ Yes<br>◯ No<br>◯ Unsure	◯ Not at all<br>◯ Mild amount<br>◯ Moderate amount<br>◯ Severe amount<br>◯ Worst possible amount
Sensitive to Light	◯ Yes<br>◯ No<br>◯ Unsure	◯ Yes<br>◯ No<br>◯ Unsure	◯ Not at all<br>◯ Mild amount<br>◯ Moderate amount<br>◯ Severe amount<br>◯ Worst possible amount
Sensitive to Noise	◯ Yes<br>◯ No<br>◯ Unsure	◯ Yes<br>◯ No<br>◯ Unsure	◯ Not at all<br>◯ Mild amount<br>◯ Moderate amount<br>◯ Severe amount<br>◯ Worst possible amount
Sensitive to Touch	◯ Yes<br>◯ No<br>◯ Unsure	◯ Yes<br>◯ No<br>◯ Unsure	◯ Not at all<br>◯ Mild amount<br>◯ Moderate amount<br>◯ Severe amount<br>◯ Worst possible amount
Sensitive to Smell	◯ Yes<br>◯ No<br>◯ Unsure	◯ Yes<br>◯ No<br>◯ Unsure	◯ Not at all<br>◯ Mild amount<br>◯ Moderate amount<br>◯ Severe amount<br>◯ Worst possible amount

SENSORY ISSUES

Are you having any body feelings or sensations that are unusual?	◯ Yes<br>◯ No<br>◯ Unsure

Please tell us whether you agree or disagree with the following statements. If you agree with the statement and believe it is true, mark Yes. If you disagree with the statement and believe it is not true, mark No. If you are unsure, mark Unsure.

Problems with body feelings or sensations are getting better over time:	◯ Yes<br>◯ No<br>◯ Unsure

Problems with body feelings or sensations are getting worse over time:	◯ Yes<br>◯ No<br>◯ Unsure
Problems with body feelings or sensations are concerning to me:	◯ Yes<br>◯ No<br>◯ Unsure
Do you now need a new medication or more medication for body feelings or sensations since your COVID-19 infection?	◯ Yes<br>◯ No

Symptom	Are you currently having this symptom?	Did this symptom first begin after you felt like you were already recovering/after the acute phase?	How much is the symptom currently bothering you?
Tingling sensations	◯ Yes<br>◯ No<br>◯ Unsure	◯ Yes<br>◯ No<br>◯ Unsure	◯ Not at all<br>◯ Mild amount<br>◯ Moderate amount<br>◯ Severe amount<br>◯ Worst possible amount
Burning Sensation	◯ Yes<br>◯ No<br>◯ Unsure	◯ Yes<br>◯ No<br>◯ Unsure	◯ Not at all<br>◯ Mild amount<br>◯ Moderate amount<br>◯ Severe amount<br>◯ Worst possible amount
Numbness	◯ Yes<br>◯ No<br>◯ Unsure	◯ Yes<br>◯ No<br>◯ Unsure	◯ Not at all<br>◯ Mild amount<br>◯ Moderate amount<br>◯ Severe amount<br>◯ Worst possible amount
Ringing in the ears/tinnitus	◯ Yes<br>◯ No<br>◯ Unsure	◯ Yes<br>◯ No<br>◯ Unsure	◯ Not at all<br>◯ Mild amount<br>◯ Moderate amount<br>◯ Severe amount<br>◯ Worst possible amount
Body stiffness	◯ Yes<br>◯ No<br>◯ Unsure	◯ Yes<br>◯ No<br>◯ Unsure	◯ Not at all<br>◯ Mild amount<br>◯ Moderate amount<br>◯ Severe amount<br>◯ Worst possible amount
Feeling swollen	◯ Yes<br>◯ No<br>◯ Unsure	◯ Yes<br>◯ No<br>◯ Unsure	◯ Not at all<br>◯ Mild amount<br>◯ Moderate amount<br>◯ Severe amount<br>◯ Worst possible amount

MOVEMENT AND STRENGTH ISSUES

Are you having any problems with controlling movements?	◯ Yes<br>◯ No<br>◯ Unsure

Please tell us whether you agree or disagree with the following statements. If you agree with the statement and believe it is true, mark Yes. If you disagree with the statement and believe it is not true, mark No. If you are unsure, mark Unsure.

Problems with controlling movements are getting better over time:	Yes<br>No<br>Unsure
Problems with controlling movements are getting worse over time:	Yes<br>No<br>Unsure
Problems with controlling movements are concerning to me:	Yes<br>No<br>Unsure
Do you now need a new medication or more medication to control movements since your COVID-19 infection?	Yes<br>No

Symptom	Are you currently having this symptom?	Did this symptom first begin after you felt like you were already recovering/after the acute phase?	How much is the symptom currently bothering you?
Feeling weak	Yes<br>No<br>Unsure	Yes<br>No<br>Unsure	Not at all<br>Mild amount<br>Moderate amount<br>Severe amount<br>Worst possible amount
Weakness in the arms and/or legs	Yes<br>No<br>Unsure	Yes<br>No<br>Unsure	Not at all<br>Mild amount<br>Moderate amount<br>Severe amount<br>Worst possible amount
Weakness in the face	Yes<br>No<br>Unsure	Yes<br>No<br>Unsure	Not at all<br>Mild amount<br>Moderate amount<br>Severe amount<br>Worst possible amount
Restless legs at night	Yes<br>No<br>Unsure	Yes<br>No<br>Unsure	Not at all<br>Mild amount<br>Moderate amount<br>Severe amount<br>Worst possible amount
Trouble starting to move	Yes<br>No<br>Unsure	Yes<br>No<br>Unsure	Not at all<br>Mild amount<br>Moderate amount<br>Severe amount<br>Worst possible amount
Movements that cannot be controlled	Yes<br>No<br>Unsure	Yes<br>No<br>Unsure	Not at all<br>Mild amount<br>Moderate amount<br>Severe amount<br>Worst possible amount
Tremors or shakes	Yes<br>No<br>Unsure	Yes<br>No<br>Unsure	Not at all<br>Mild amount<br>Moderate amount<br>Severe amount<br>Worst possible amount

Seizures	Yes<br>No<br>Unsure	Yes<br>No<br>Unsure	Not at all<br>Mild amount<br>Moderate amount<br>Severe amount<br>Worst possible amount
Body twists on itself	Yes<br>No<br>Unsure	Yes<br>No<br>Unsure	Not at all<br>Mild amount<br>Moderate amount<br>Severe amount<br>Worst possible amount
Trouble making sounds when talking	Yes<br>No<br>Unsure	Yes<br>No<br>Unsure	Not at all<br>Mild amount<br>Moderate amount<br>Severe amount<br>Worst possible amount
Feeling off-balance	Yes<br>No<br>Unsure	Yes<br>No<br>Unsure	Not at all<br>Mild amount<br>Moderate amount<br>Severe amount<br>Worst possible amount
Falls	Yes<br>No<br>Unsure	Yes<br>No<br>Unsure	Not at all<br>Mild amount<br>Moderate amount<br>Severe amount<br>Worst possible amount
Trouble keeping balance when walking	Yes<br>No<br>Unsure	Yes<br>No<br>Unsure	Not at all<br>Mild amount<br>Moderate amount<br>Severe amount<br>Worst possible amount

MOOD ISSUES

Are you having problems with mood, feelings, or emotions?	Yes<br>No<br>Unsure
Please tell us whether you agree or disagree with the following statements. If you agree with the statement and believe it is true, mark Yes. If you disagree with the statement and believe it is not true, mark No. If you are unsure, mark Unsure.	
Problems with mood, feelings, and emotions are getting better over time:	Yes<br>No<br>Unsure
Problems with mood, feelings, and emotions are getting worse over time:	Yes<br>No<br>Unsure
Problems with mood, feelings, and emotions are concerning to me:	Yes<br>No<br>Unsure
Do you now need a new medication or more medication to help mood, feelings, and emotions since your COVID-19 infection?	Yes<br>No

Symptom	Are you currently having this symptom?	Did this symptom first begin	How much is the symptom currently bothering you?

		after you felt like you were already recovering/after the acute phase?	
Anxious	Yes<br>No<br>Unsure	Yes<br>No<br>Unsure	Not at all<br>Mild amount<br>Moderate amount<br>Severe amount<br>Worst possible amount
Depressed	Yes<br>No<br>Unsure	Yes<br>No<br>Unsure	Not at all<br>Mild amount<br>Moderate amount<br>Severe amount<br>Worst possible amount
Easily irritated	Yes<br>No<br>Unsure	Yes<br>No<br>Unsure	Not at all<br>Mild amount<br>Moderate amount<br>Severe amount<br>Worst possible amount
Loss of interest in sex	Yes<br>No<br>Unsure	Yes<br>No<br>Unsure	Not at all<br>Mild amount<br>Moderate amount<br>Severe amount<br>Worst possible amount
Mood swings	Yes<br>No<br>Unsure	Yes<br>No<br>Unsure	Not at all<br>Mild amount<br>Moderate amount<br>Severe amount<br>Worst possible amount
Personality change	Yes<br>No<br>Unsure	Yes<br>No<br>Unsure	Not at all<br>Mild amount<br>Moderate amount<br>Severe amount<br>Worst possible amount

EYE AND VISION ISSUES

Are you having problems with eyes or vision?	Yes<br>No<br>Unsure
Please tell us whether you agree or disagree with the following statements. If you agree with the statement and believe it is true, mark Yes. If you disagree with the statement and believe it is not true, mark No. If you are unsure, mark Unsure.	
Problems with eyes or vision are getting better over time:	Yes<br>No<br>Unsure
Problems with eyes or vision are getting worse over time:	Yes<br>No<br>Unsure
Problems with eyes or vision are concerning to me:	Yes<br>No<br>Unsure
Do you now need a new medication, more medication, or new glasses prescription to help eyes or vision since your COVID-19 infection?	Yes<br>No

Symptom	Are you currently having this symptom?	Did this symptom first begin after you felt like you were already recovering/after the acute phase?	How much is the symptom currently bothering you?
Eye discharge	Yes<br>No<br>Unsure	Yes<br>No<br>Unsure	Not at all<br>Mild amount<br>Moderate amount<br>Severe amount<br>Worst possible amount
Eye redness	Yes<br>No<br>Unsure	Yes<br>No<br>Unsure	Not at all<br>Mild amount<br>Moderate amount<br>Severe amount<br>Worst possible amount
Feeling of something in your eye	Yes<br>No<br>Unsure	Yes<br>No<br>Unsure	Not at all<br>Mild amount<br>Moderate amount<br>Severe amount<br>Worst possible amount
Itching of the eyes	Yes<br>No<br>Unsure	Yes<br>No<br>Unsure	Not at all<br>Mild amount<br>Moderate amount<br>Severe amount<br>Worst possible amount
Eye pain	Yes<br>No<br>Unsure	Yes<br>No<br>Unsure	Not at all<br>Mild amount<br>Moderate amount<br>Severe amount<br>Worst possible amount
Blurred vision	Yes<br>No<br>Unsure	Yes<br>No<br>Unsure	Not at all<br>Mild amount<br>Moderate amount<br>Severe amount<br>Worst possible amount
See specks or flashes of light	Yes<br>No<br>Unsure	Yes<br>No<br>Unsure	Not at all<br>Mild amount<br>Moderate amount<br>Severe amount<br>Worst possible amount

SKIN AND HAIR ISSUES

Are you having problems with skin or hair?	Yes<br>No<br>Unsure

Please tell us whether you agree or disagree with the following statements. If you agree with the statement and believe it is true, mark Yes. If you disagree with the statement and believe it is not true, mark No. If you are unsure, mark Unsure.

Problems with skin or hair are getting better over time:	Yes<br>No<br>Unsure

Problems with skin or hair are getting worse over time:	◯ Yes<br>◯ No<br>◯ Unsure
Problems with skin or hair are concerning to me:	◯ Yes<br>◯ No<br>◯ Unsure
Do you now need a new medication or more medication to help skin or hair since your COVID-19 infection?	◯ Yes<br>◯ No

Symptom	Are you currently having this symptom?	Did this symptom first begin <u>after</u> you felt like you were already recovering/after the acute phase?	How much is the symptom <u>currently</u> bothering you?
Painful toes that changed color	◯ Yes<br>◯ No<br>◯ Unsure	◯ Yes<br>◯ No<br>◯ Unsure	◯ Not at all<br>◯ Mild amount<br>◯ Moderate amount<br>◯ Severe amount<br>◯ Worst possible amount
Sores or ulcers in your mouth	◯ Yes<br>◯ No<br>◯ Unsure	◯ Yes<br>◯ No<br>◯ Unsure	◯ Not at all<br>◯ Mild amount<br>◯ Moderate amount<br>◯ Severe amount<br>◯ Worst possible amount
Skin rash	◯ Yes<br>◯ No<br>◯ Unsure	◯ Yes<br>◯ No<br>◯ Unsure	◯ Not at all<br>◯ Mild amount<br>◯ Moderate amount<br>◯ Severe amount<br>◯ Worst possible amount
Hair loss	◯ Yes<br>◯ No<br>◯ Unsure	◯ Yes<br>◯ No<br>◯ Unsure	◯ Not at all<br>◯ Mild amount<br>◯ Moderate amount<br>◯ Severe amount<br>◯ Worst possible amount

OPTIONAL DESCRIPTION:

Please take some time to describe in the textbox how you recall your experience of recovery after your recent COVID-19 infection. This is an opportunity to help us better understand the health problems you are experiencing during your recovery in your own words. Please tell us about the things that happened and the things that surprised you most during your recovery that we have not asked about above.	

Test eForm rules

Show hidden eForm elements

PROMIS SF v1.1 - Global Health

PROMIS SF v1.1 - Global Health CAT

Main

Visit date: Format: YYYY-MM-DD HH:MM

Additional information (if any):

Final Results

Total T-score

Total Standard Error

Q: Global01

In general, would you say your health is:

- Excellent
- Very good
- Good
- Fair
- Poor

Q: Global02

In general, would you say your quality of life is:

- Excellent
- Very good
- Good
- Fair
- Poor

Q: Global03

In general, how would you rate your physical health?

- Excellent
- Very good
- Good
- Fair
- Poor

Q: Global04

In general, how would you rate your mental health, including your mood and your ability to think?

- Excellent
- Very good
- Good
- Fair
- Poor

Q: Global05

In general, how would you rate your satisfaction with your social activities and relationships?

- Excellent
- Very good
- Good
- Fair
- Poor

Q: Global06

To what extent are you able to carry out your everyday physical activities such as walking, climbing stairs, carrying groceries, or moving a chair?

- Completely
- Mostly
- Moderately
- A little
- Not at all

Q: Global07

In the past 7 days, how would you rate your pain on average? [0 - No Pain, 10 - Worst possible pain]

- 0
- 1
- 2
- 3
- 4
- 5
- 6
- 7
- 8
- 9
- 10

Q: Global08

In the past 7 days, how would you rate your fatigue on average?

- None
- Mild
- Moderate
- Severe
- Very severe

Q: Global09r

In general, please rate how well you carry out your usual social activities and roles. (This includes activities at home, at work, and in your community, and responsibilities as a parent, ch spouse, employee, friend, etc.)

- Excellent

- Very good
- Good
- Fair
- Poor

Q: Global10

In the past 7 days, how often have you been bothered by emotional problems such as feeling anxious, depressed, or irritable?

- Never
- Rarely
- Sometimes
- Often
- Always

Test eForm rules

Show hidden eForm elements

POLYSYMPTOMATIC DISTRESS SCALE

Widespread Pain Index (WPI)

Please mark yes or no to indicate if you have had pain in each of the following areas over the last week.

*Shoulder Girdle, Left	Yes / No	*Shoulder Girdle, Right	Yes / No
*Upper Arm, Left	Yes / No	*Upper Arm, Right	Yes / No
*Lower Arm, Left	Yes / No	*Lower Arm, Right	Yes / No
*Hip (buttock, trochanter), Left	Yes / No	*Hip (buttock, trochanter), Right	Yes / No
*Upper Leg, Left	Yes / No	*Upper Leg, Right	Yes / No
*Lower Leg, Left	Yes / No	*Lower Leg, Right	Yes / No
*Jaw, Left	Yes / No	*Jaw, Right	Yes / No
*Chest	Yes / No	*Abdomen	Yes / No
*Upper Back	Yes / No		
*Lower Back	Yes / No		
*Neck	Yes / No		
WPI Score (1-19):			

Symptoms Severity Score (SS)

2a) For the each of the three symptoms below, indicate the level of severity over the past week using the following scale:

*Fatigue
- 0 = No problem
- 1 = Slight or mild problems; generally mild or intermittent

2 = Moderate; considerable problems; often present and/or at a moderate level
3 = Severe: pervasive, continuous, life-disturbing problems

*Waking unrefreshed	0 = No problem<br>1 = Slight or mild problems; generally mild or intermittent<br>2 = Moderate; considerable problems; often present and/or at a moderate level<br>3 = Severe: pervasive, continuous, life-disturbing problems
*Cognitive symptoms	0 = No problem<br>1 = Slight or mild problems; generally mild or intermittent<br>2 = Moderate; considerable problems; often present and/or at a moderate level<br>3 = Severe: pervasive, continuous, life-disturbing problems

2b) For each of the three symptoms below, indicate if you have had the symptom during the **previous six months**

*Headaches	Yes<br>No
*Pain or Cramps in lower abdomen	Yes<br>No
*Depression	Yes<br>No
Symptom Severity Score:	

Total Score

Total Score:	

Test eForm rules

Show hidden eForm elements

PROMIS SF v1.0 - Depression 8b

PROMIS SF v1.0 - Depression 8b auto scoring

Main

Visit date:	Format: YYYY-MM-DD HH:MM
Additional information (if any):	

Final Results

Total T-score	
Total Standard Error	

Q: EDDEP04

In the past 7 days, I felt worthless.

- Never
- Rarely
- Sometimes
- Often
- Always

Q: EDDEP05

In the past 7 days, I felt that I had nothing to look forward to.

- Never
- Rarely
- Sometimes
- Often
- Always

Q: EDDEP06

In the past 7 days, I felt helpless.

- Never
- Rarely
- Sometimes
- Often
- Always

Q: EDDEP17

View eForm

In the past 7 days, I felt sad.

- Never
- Rarely
- Sometimes
- Often
- Always

Q: EDDEP22

In the past 7 days, I felt like a failure.

- Never
- Rarely
- Sometimes
- Often
- Always

Q: EDDEP29

In the past 7 days, I felt depressed.

- Never
- Rarely
- Sometimes
- Often
- Always

Q: EDDEP36

In the past 7 days, I felt unhappy.

- Never
- Rarely
- Sometimes
- Often
- Always

Q: EDDEP41

In the past 7 days, I felt hopeless.

- Never
- Rarely
- Sometimes
- Often
- Always

View eForm

Test eForm rules

Show hidden eForm elements

PROMIS SF v1.0-Anxiety 8a

PROMIS SF v1.0-Anxiety 8a Auto Scoring

Main

Visit date:	Format: YYYY-MM-DD HH:MM
Additional information (if any):	

Q: EDANX01

In the past 7 days, I felt fearful.

- Never
- Rarely
- Sometimes
- Often
- Always

Q: EDANX40

In the past 7 days, I found it hard to focus on anything other than my anxiety.

- Never
- Rarely
- Sometimes
- Often
- Always

Q: EDANX41

In the past 7 days, my worries overwhelmed me.

- Never
- Rarely
- Sometimes
- Often
- Always

Q: EDANX53

In the past 7 days, I felt uneasy.

- Never
- Rarely
- Sometimes
- Often
- Always

Q: EDANX46

In the past 7 days, I felt nervous.

- Never

View eForm

- Rarely
- Sometimes
- Often
- Always

Q: EDANX07

In the past 7 days, I felt like I needed help for my anxiety.	◯ Never<br>◯ Rarely<br>◯ Sometimes<br>◯ Often<br>◯ Always

Q: EDANX05

In the past 7 days, I felt anxious.	◯ Never<br>◯ Rarely<br>◯ Sometimes<br>◯ Often<br>◯ Always

Q: EDANX54

In the past 7 days, I felt tense.	◯ Never<br>◯ Rarely<br>◯ Sometimes<br>◯ Often<br>◯ Always

Final Results

Total T-score	
Total Standard Error	
