# Supplementary material for: Prevalence and severity of neurologic symptoms in Long-COVID and the role of pre-existing conditions, hospitalization, and mental health
Source: Front Neurol. 2025 Jun 25;16:1562084. doi: 10.3389/fneur.2025.1562084 (PMC12237652; doi:10.3389/fneur.2025.1562084)
Supplement: Supplementary file 3 [file Table_1.docx]

**Supplemental Table 1: Assessment of Symptom Prevalence, Severity, Course, Pre-existing Conditions, and Test Status**

| **Symptom Prevalence** | |
| --- | --- |
| Questionnaire Item | Response Options |
| Pre COVID: “Did you have this symptom during the three months before your recent COVID-19 infection?”  During COVID: “Did you have this symptom when you were recently infected?”  Post COVID: “Are you currently having this symptom?” | Yes (go to severity item)  No  Unsure |
| **Symptom Severity** | |
| Questionnaire Item | Response Options |
| Pre COVID: “How much did [symptom] bother you in the three months before your recent COVID-19 infection?”  During COVID: “When the symptom was at its worst, how much did it bother you?”  Post COVID: “How much is the symptom currently bothering you?” | *1=Not at All  2=Mild Amount  3=Moderate Amount  4=Severe Amount  5=Worst Possible Amount |
| **Neurologic Symptoms** | |
| Infectious: cough, fever, flu-like symptoms, chills  General: loss of appetite, dizziness/lightheadedness, fatigue, general weakness, hair loss, nausea/vomiting, rash  Sleep: difficulty staying awake, trouble sleeping, unrefreshed sleep  Neurocognitive: concentration/memory difficulties, dysarthria/word finding difficulty  Focal: balance/coordination difficulty, focal weakness  Sensory: blurred vision, see specks or flashes of light, light sensitivity, noise sensitivity, numbness/tingling, touch sensitivity  Pain Syndromes: arthralgia, headache, myalgia  Psych: delirium, hallucinations | |
| **Pre-existing Conditions** | |
| Questionnaire Item | Response Options |
| “Before your recent COVID-19 infection, have you ever been told by a doctor or other health professional that you have [condition]? | Yes  No  Unsure |
| **Pre-existing Conditions Groupings Based on Past Medical History** | |
| Neurological*:* stroke/transient ischemic attack, seizure/epilepsy, multiple sclerosis, Parkinson’s disease, Guillain-Barre syndrome, Myasthenia Gravis, dementia/Alzheimer’s, migraine/headaches, concussion, peripheral neuropathy, POTS/exercise-induced hypotension  Psychiatric: anxiety, depression, ADHD | |
| **Test Status** | |
| Test Positive participants: positive results on either a SARS-CoV-2 Polymerase Chain Reaction (PCR), home lateral flow/Nucleic Acid Amplification (NAA) test (photo sent for confirmation), or laboratory antibody test. For laboratory antibody tests, if patient was unvaccinated at time of infection, surface or nucleocapsid antibody presence was accepted for confirmation, if patient was vaccinated at time of infection, nucleocapsid antibody presence was required for confirmation.  Test Negative participants: had medical record documentation of a SARS CoV-2 infection based on CDC clinical criteria^8^ with either an absent or negative SARS-CoV-2 test and a negative antibody test. | |
| **PROMIS Depression/Anxiety** | |
| <55: none to slight  55-55.9: mild  60-69.9: moderate  >/=70: severe | |

* Participants who chose “not at all” were then recoded as not having the symptom. For qualifying a symptom as severe in analysis of risk in preexisting conditions, “severe amount” and “worst possible amount” were grouped together as “severe symptom.”
