# Supplementary material for: Prevalence and severity of neurologic symptoms in Long-COVID and the role of pre-existing conditions, hospitalization, and mental health
Source: Front Neurol. 2025 Jun 25;16:1562084. doi: 10.3389/fneur.2025.1562084 (PMC12237652; doi:10.3389/fneur.2025.1562084)
Supplement: Supplementary file 4 [file Table_2.docx]

Supplemental Table 2: Demographics and Infection Information

|  | **Cohort (N=213)** | |
| --- | --- | --- |
|  | **Long-COVID** | **NHIS 2020^e^** |
| **Age group** | **N (%)** | **%** |
| 18-29 | 18 (8.5) | 20.5 |
| 30-39 | 61 (28.6) | 17.0 |
| 40-49 | 47 (22.1) | 15.9 |
| 50-59 | 50 (23.5) | 16.4 |
| 60-69 | 28 (13.1) | 15.3 |
| 70+ | 9 (4.2) | 14.8 |
| **Sex** |  |  |
| Female | 153 (71.8) | 48.3 |
| Male | 60 (28.2) | 51.7 |
| **Ethnicity** |  |  |
| Hispanic | 11 (5.2) | 16.8 |
| Non-Hispanic | 194 (91.1) | 83.3 |
| Unknown | 8 (3.7) | - |
| **Race** |  |  |
| American Indian/Alaskan Native | 3 (1.4) | 1 |
| Asian | 9 (4.2) | 6.0 |
| Black/African American | 3 (1.4) | 12.1 |
| Other racial groups that are not listed and/or Multiracial | 14 (6.6) | 2.4 |
| White | 184 (86.4) | 71.6 |
| Unknown | - | 6.9 |
| **Highest education level completed** |  |  |
| Less than high school graduate | 0 (0) | 11.8 |
| High school/GED | 6 (2.8) | 28.2 |
| Some college/Associate degree | 36 (16.9) | 30.2 |
| Bachelor’s degree or higher | 170 (79.8) | 29.2 |
| Unknown | 1 (0.47) | 0.7 |
| **Health Insurance**^a^ |  |  |
| Private Health Insurance | 160 (75.1) | 61.8 |
| Public health insurance | 52 (24.4) | 38.0 |
| Uninsured | 1 (0.47) | 9.7 |
| Unknown | - | - |
| **2019 Annual Household Income** |  |  |
| Less than 35,000 | 14 (6.6) | 23 .2 |
| $35, 000 to $49, 999 | 9 (4.2) | 12.8 |
| $50, 000 to $74, 999 | 23 (10.8) | 18.4 |
| $75,000 or more | 146 (68.5) | 45.6 |
| Unknown and/or refuse to answer | 21 (9.9) | - |
| **Elapsed time since infection; min,max** | min:1 month, max:30 months |  |
| **Elapsed Time Since Infection^b^** |  |  |
| 1 to 3 months | 31 (14.5) |  |
| 4 to 6 months | 52 (24.4) |  |
| 7 to 9 months | 38 (17.8) |  |
| 10 to 12 months | 47 (22.1) |  |
| 12< months | 45 (21.1) |  |
| **Variants Dates^c^** |  |  |
| Alpha (1/20 – 12/2020): | 149 (70.0) |  |
| Beta/Gamma (1/2021 – 4/2021): | 24 (11.3) |  |
| Delta (5/2021 – 10/2021) | 9 (4.2) |  |
| Omicron (11/2021- Present): | 31 (14.5) |  |
| **WHO Severity Category^d^** |  |  |
| 2- symptomatic; independent | 44 (20.7) |  |
| 3- symptomatic; assistance needed | 136 (63.8) |  |
| 4- hospitalized; no oxygen therapy | 9 (4.2) |  |
| 5- hospitalized, oxygen by mask or nasal prongs | 22 (10.3) |  |
| 6- hospitalized; oxygen by NIV or high flow | 2 (1.0) |  |

^a^ Cohen RA, Terlizzi EP, Cha AE, Martinez ME. Health insurance coverage: Early release of estimates from the National Health Interview Survey, 2020. National Center for Health Statistics. August 2021. DOI: <https://dx.doi.org/10.15620/cdc:108816>.

^b^ Elapsed time since infection= time between positive test and date of questionnaire completion

^c^ CDC.CDC Museum COVID-19 Timeline.2023

^d^ Marshall, J. C., Murthy, S., Diaz, J., Adhikari, N. K., Angus, D. C., Arabi, Y. M., ... & Zhang, J. (2020). A minimal common outcome measure set for COVID-19 clinical research. The Lancet Infectious Diseases, 20(8), e192-e197.

^e^ NHIS data analyses were conducted using SAS version 9.4 and were weighted using the sample adult weight.
