# Supplementary material for: Prevalence and severity of neurologic symptoms in Long-COVID and the role of pre-existing conditions, hospitalization, and mental health
Source: Front Neurol. 2025 Jun 25;16:1562084. doi: 10.3389/fneur.2025.1562084 (PMC12237652; doi:10.3389/fneur.2025.1562084)
Supplement: Supplementary file 5 [file Table_3.docx]

**Supplemental Table 3: Odds of patients living with a pre-existing psychiatric or neurologic condition**

| **Current Psychiatric Status** | | **N** | **Pre-existing Psychiatric Condition** | | **Pre-existing Neurologic Condition** | |
| --- | --- | --- | --- | --- | --- | --- |
|  |  |  | **Yes** | **No** | **Yes** | **No** |
| Anxiety | Moderate to Severe | 68 | 44 | 24 | 30 | 38 |
|  | Mild | 34 | 15 | 19 | 8 | 26 |
|  | None | 99 | 37 | 62 | 28 | 71 |
| Depression | Moderate to Severe | 54 | 35 | 19 | 22 | 32 |
|  | Mild | 44 | 24 | 20 | 12 | 32 |
|  | None | 103 | 37 | 66 | 32 | 71 |
| Odds of Moderate-Severe | Anxiety Only | 28 | 2.6 (1.1-6.1); p=0.02 | Reference | 2.0 (0.9-4.8); p=0.1 | Reference |
|  | Depression Only | 14 | 2.3 (0.7-7.0); p=0.15 | Reference | 1.1 (0.3-3.7); p=0.89 | Reference |
|  | Anxiety and Depression | 40 | 3.5 (1.7-7.6); p=0.001 | Reference | 2.2 (1.1-4.7); p=0.04 | Reference |
